# Protein 3D Structure Computed from Evolutionary Sequence Variation

**DOI:** 10.1371/journal.pone.0028766

**Published:** 2011-12-07

**Authors:** Debora S. Marks, Lucy J. Colwell, Robert Sheridan, Thomas A. Hopf, Andrea Pagnani, Riccardo Zecchina, Chris Sander

**Affiliations:** 1 Department of Systems Biology, Harvard Medical School, Boston, Massachusetts, United States of America; 2 MRC Laboratory of Molecular Biology, Hills Road, Cambridge, United Kingdom; 3 Computational Biology Center, Memorial Sloan-Kettering Cancer Center, New York, New York, United States of America; 4 Human Genetics Foundation, Torino, Italy; 5 Politecnico di Torino, Torino, Italy; University of California San Francisco, United States of America

## Abstract

The evolutionary trajectory of a protein through sequence space is constrained by its function. Collections of sequence homologs record the outcomes of millions of evolutionary experiments in which the protein evolves according to these constraints. Deciphering the evolutionary record held in these sequences and exploiting it for predictive and engineering purposes presents a formidable challenge. The potential benefit of solving this challenge is amplified by the advent of inexpensive high-throughput genomic sequencing.

In this paper we ask whether we can infer evolutionary constraints from a set of sequence homologs of a protein. The challenge is to distinguish true co-evolution couplings from the noisy set of observed correlations. We address this challenge using a maximum entropy model of the protein sequence, constrained by the statistics of the multiple sequence alignment, to infer residue pair couplings. Surprisingly, we find that the strength of these inferred couplings is an excellent predictor of residue-residue proximity in folded structures. Indeed, the top-scoring residue couplings are sufficiently accurate and well-distributed to define the 3D protein fold with remarkable accuracy.

We quantify this observation by computing, from sequence alone, all-atom 3D structures of fifteen test proteins from different fold classes, ranging in size from 50 to 260 residues., including a G-protein coupled receptor. These blinded inferences are *de novo*, i.e., they do not use homology modeling or sequence-similar fragments from known structures. The co-evolution signals provide sufficient information to determine accurate 3D protein structure to 2.7–4.8 Å C_α_-RMSD error relative to the observed structure, over at least two-thirds of the protein (method called EVfold, details at http://EVfold.org). This discovery provides insight into essential interactions constraining protein evolution and will facilitate a comprehensive survey of the universe of protein structures, new strategies in protein and drug design, and the identification of functional genetic variants in normal and disease genomes.

## Introduction

### Exploiting the evolutionary record in protein families

**Figure 1:**
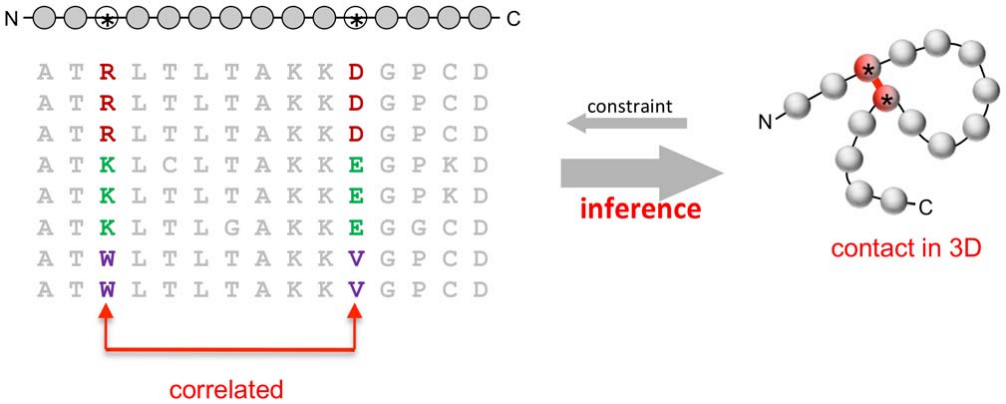
Correlated mutations carry information about distance relationships in protein structure. The sequence of the protein for which the 3D structure is to be predicted (each circle is an amino acid residue, typical sequence length is 50–250 residues) is part of an evolutionarily related family of sequences (amino acid residue types in standard one-letter code) that are presumed to have essentially the same fold (iso-structural family). Evolutionary variation in the sequences is constrained by a number of requirements, including the maintenance of favorable interactions in direct residue-residue contacts (red line, right). The inverse problem of protein fold prediction from sequence addressed here exploits pair correlations in the multiple sequence alignment (left) to deduce which residue pairs are likely to be close to each other in the three-dimensional structure (right). A subset of the predicted residue contact pairs is subsequently used to fold up any protein in the family into an approximate predicted 3D shape (‘fold’) which is then refined using standard molecular physics techniques, yielding a predicted all-atom 3D structure of the protein of interest.

The evolutionary process constantly samples the space of possible sequences and, by implication, structures consistent with a functional protein in the context of a replicating organism. Homologous proteins from diverse organisms can be recognized by sequence comparison because strong selective constraints prevent amino acid substitutions in particular positions from being accepted. The beauty of this evolutionary record, reported in protein family databases such as PFAM [1], is the balance between sequence exploration and constraints: conservation of function within a protein family imposes strong boundaries on sequence variation and generally ensures similarity of 3D structure among all family members [2] (Figure 1).

In particular, to maintain energetically favorable interactions, residues in spatial proximity may co-evolve across a protein family [2], [3]. This suggests that residue correlations could provide information about amino acid residues that are close in structure [4], [5], [6], [7], [8], [9], [10], [11]. However, correlated residue pairs within a protein are not necessarily close in 3D space. Confounding residue correlations may reflect constraints that are not due to residue proximity but are nevertheless true biological evolutionary constraints or, they could simply reflect correlations arising from the limitations of our insight and technical noise. Evolutionary constraints on residues involved in oligomerization, protein-protein, or protein-substrate interactions or other spatially indirect or spatially distributed interactions can result in co-variation between residues not in close spatial proximity within a protein monomer. In addition, the principal technical causes of confounding residue correlations are transitivity of correlations, statistical noise due to small numbers and phylogenetic sampling bias in the set of sequences assembled in the protein family [12], [13], [14], [15]. One does not know *a priori* the relative contributions of these possible causes of co-variation effects and is thus faced with the complicated inverse problem of using observed correlations to infer contacts between residues (Figure 1). Given alternative causes of true evolutionary co-variation, even if confounding correlations caused by technical reasons can be identified, there is no guarantee that the remaining correlated residue pairs will be dominated by residues in three dimensional proximity.

The initial challenge is thus to solve the inverse sequence-to-structure problem by reducing the influence of confounding factors. Only then is it possible to judge whether the evolutionary process reveals enough residue contacts, which are sufficiently evenly distributed (spread) throughout the protein sequence and structure, to predict the protein fold. The ultimate criterion of performance is the accuracy of 3D structure prediction using the inferred contacts. Previous work combined a small number of evolutionarily inferred residue contacts with other, structural, sources of information to successfully predict the structure of some smaller proteins, [16], [17], [18], [19]. However, three crucial open questions remain with respect to using evolutionarily inferred residue-residue couplings for protein fold prediction. The first is whether one can develop a sufficiently robust method to identify causative correlations that reflect evolutionary constraints. The second is whether the inferred, plausibly evolutionary, correlations primarily reflect residue-residue proximity. The third is whether these inferred residue-residue proximities provide sufficient information to predict a protein fold, without the use of known three-dimensional structures.

### The *de novo* protein structure prediction problem in the era of genome sequencing

Solving this inverse problem would enable novel insight into the evolutionary dynamics of sequence variation, and the role of evolutionarily constrained interactions in protein folding. Determination of protein structure, by experiment or theory, provides one essential window into protein function, evolution and design. However, our knowledge of protein structure remains incomplete and is far from saturation. In spite of significant progress in the field of structural genomics over the last decade [20], only about half of all well-characterized protein families (PFAM-A, 12,000 families), have a 3D structure for any of their members [1]. At the same time, the current upper limit on the total number of protein families (∼200,000; PFAM-B) is an order of magnitude larger, and continues to grow with no clear limit in sight. Therefore, as massive genomic sequencing projects rapidly increase the number and size of protein families, in particular those without structural homologs [21], accurate *de novo* prediction of 3D structure from sequence would rapidly expand our overall knowledge of protein structures in a way difficult to achieve by experiment.

### Limited ability of current de novo 3D structure prediction methods

Although the challenge of the computational sequence-to-structure problem remains unsolved, methods that use fragment libraries [22], [23] or other strategies to search conformational space [24], [25], followed by sophisticated energy optimization or molecular dynamics refinement, have been successful at predicting the 3D structures of smaller proteins (<80 residues) [22], [24], [25], [26] [25], [27], [28]. In addition, custom-designed supercomputers have allowed insight not only into molecular dynamics of protein function, but also into the folding pathways of smaller proteins such as BPTI and WW domains [29], [30]. However, none of these computational approaches have yet achieved *de novo* folding from a disordered or extended polypeptide to the native folded state for larger proteins and it is generally appreciated that the primary obstacle to 3D protein structure prediction is conformational sampling, i.e., successful search of the vast space of protein conformations for the correct fold [26], [31]. Using current methods, it is computationally infeasible to adequately sample the enormous set of all 3D configurations a protein might explore in the process of folding to the native state. In this paper we explore the idea that information gleaned from statistical analysis of multiple sequence alignments can be used to solve this problem [2], [5], [6], [32], [33]. The goal is use residue-residue contacts inferred from the evolutionary record (EICs) to identify the tiny region in the space of all possible 3D configurations of a given protein that contains the correctly folded or ‘native’ structure.

### Extracting essential information from the evolutionary sequence record using global statistical models

Statistical physics and computer science have developed a number of methods that address the problem of inferring a statistical model for a given set of empirically measured observables. A partial analogy can be drawn to the inverse Ising or Potts problem, in which heterogeneous local couplings between discrete state variables are derived from measurements of two-point correlation functions [34], [35], [36], [37], [38]. Similar maximum entropy methods have been applied to problems in neurobiology, e.g., for the engineering of stable and fast-folding proteins [39], for the analysis of correlated network states in neural populations [40], regulatory gene network modeling from transcript profiles [41], to extract residue-residue interactions from nucleotide sequences [42]–[43]), as well as derivation of protein signaling networks from phospho-proteomics data [44]. The maximum entropy principle, which requires maximally even probabilities subject to optimal agreement between model-generated and empirical observables, turns out to be a very useful device for approaching the problem of extracting essential pair couplings from multiple sequence alignments of families of homologous proteins. The use of a maximum entropy approach to derive essential residue correlations in proteins was introduced in 1999 by Lapedes et al. [14], [15] and then implemented algorithmically using belief propagation to infer residue-residue interactions in protein-protein interfaces by Weigt et al. [11] and using Monte Carlo optimization to study sequence diversity in antibodies by Mora et al. [45]. An alternative method developed by van Nimwegen et al. [46], similar in intent but different in statistical approach, uses a Bayesian network framework to disentangle direct from indirect statistical dependencies between residue positions and also reports a dramatic improvement in the accuracy of contact prediction from multiple sequence alignments of proteins [13].

### Solving the problem of conformational complexity

On this background, we asked if there is sufficient contact information in pairwise correlations from the evolutionary sequence record to fold a protein into a correct three-dimensional structure. Our approach builds on an efficient algorithm to compute the pair couplings in a maximum entropy model, called mean field direct coupling analysis [47] and translates the resulting residue couplings to a set of distance constraints for effective use in distance geometry generation of 3D structures and in their refinement by energy minimization and molecular dynamics methods [48]. The essential data requirement for success of this process is the availability of rich evolutionary sequence data that is sufficiently diverse to reveal co-evolution patterns in amino acid residues covering most structural elements of the protein. The practical goal is to use this rich evolutionary sequence information together with a global statistical model to massively reduce the huge search space of possible protein conformations.

### Testing the information content in residue co-variation about 3D structure

**Figure 2:**
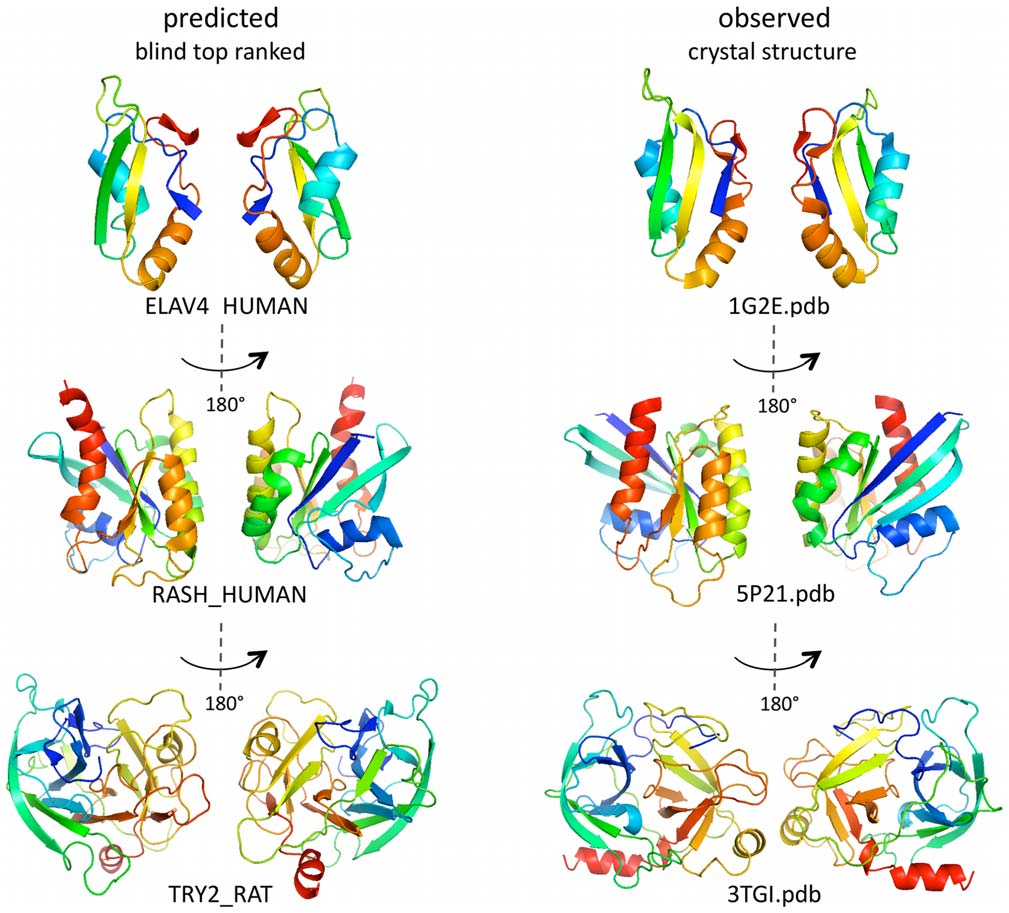
Predicted 3D structures for three representative proteins. Visual comparison of 3 of the 15 test proteins (others in Figure S3) reveals the remarkable agreement of the predicted top ranked 3D structure (left) and the experimentally observed structure (right). Center: C_α_-RMSD error and, in parentheses, number of residues used for C_α_-RMSD error calculation, e.g., 2.9 Å C_α_-RMSD (67). The ribbon representation was chosen to highlight the overall topographical progression of the polypeptide chain, rather than atomic details such as hydrogen bonding (colored blue to red in rainbow colors along the chain, N-term to C-term; helical ribbons are α-helices, straight ribbons are β-strands, arrow in the direction of the chain; each structure in front and back view, related by 180 degree rotation). The predicted proteins can be viewed in full atomic detail in deposited graphics sessions for the Pymol program (Web Appendix A4) or from their coordinates (Web Appendix A).

We test the predictive power of this approach by generating a set of candidate structures for proteins over a range of protein sizes and different folds, including a trans-membrane protein, from sequence information alone, i.e., without the use of templates or fragment libraries. We quantitatively assess the extent to which predicted 3D structures have the correct spatial arrangement of α-helices and β-strands, as compared to the experimentally determined structures. We report the details of these blinded predictions, for 15 protein structures ranging from 48 to 258 amino acids in size and indicate how the method can be used to effectively generate rich protein structural information from sufficiently large and diverse protein family alignments (Figure 2, Table 1). We conclude, based on our results and on the ability of high-throughput sequencing to radically augment evolutionary sequence information for different protein families, that prediction of 3D protein structures from evolutionary co-variation is entirely achievable and applicable to a rapidly increasing number of protein families of unknown structure.

**Table 1:** Accuracy of predicted proteins.

Target Protein Uniprot ID	Fold	L*	Pfam ID	No. seqs	Blind top Ca-rmsd**	TM***	Best Ca-rmsd**	TM***	TP****	Ref. PDB
RASH_HUMAN	a/b	161	Ras	10K	3.5 (161)	0.7	2.8 (155)	0.76	0.8	5p21
CHEY_ECOLI	a/b	114	Response_reg	72K	2.98 (107)	0.65	2.96 (107)	0.67	0.67	1e6k
THIO_ALIAC	a/b	103	Thioredoxin	13K	3.86 (94)	0.55	3.5 (97)	0.59	0.68	1rqm
RNH_ECOLI	a/b	141	RNase_H	11K	4.0 (110)	0.54	3.5 (114)	0.57	0.68	1f21
TRY2_RAT	b	223	Trypsin	16K	4.27 (186)	0.6	4.27 (186)	0.54	0.81	3tgi
CADH1_HUMAN	b	100	Cadherin	12K	3.8 (88)	0.55	3.86 (96)	0.57	0.86	2o72
YES_HUMAN	b	48	SH3_1	6K	3.6 (47)	0.37	3.35 (43)	0.41	0.52	2hda
O45418_CAEEL	a+b	100	FKBP_C	8K	4.1 (88)	0.48	3.4 (79)	0.53	0.77	1r9h
ELAV4_HUMAN	a+b	71	RRM_1	28K	2.9 (67)	0.57	3.16 (71)	0.59	0.71	1g2e
A8MVQ9_HUMAN	a+b	107	Lectin_C	5K	4.8 (85)	0.39	4.0 (100)	0.53	0.8	2it6
PCBP1_HUMAN	a+b	63	KH_1	9K	4.69 (46)	0.25	4.61 (61)	0.35	0.47	1wvn
OPSD_BOVIN	a tm	258	7tm_1	27K	4.84 (171)	0.5	4.29 (180)	0.55	0.38	1hzx
BPT1_BOVIN	a+b	52	Kunitz_BPTI	2K	2.73 (53)	0.49	2.75 (53)	0.49	0.71	5pti
OMPR_ECOLI	a	77	Trans_reg_C	24K	4.7 (64)	0.35	3.9 (62)	0.45	0.38	1odd
SPTB2_HUMAN	a	108	CH(calp hom)	4K	4.0 (47)	0.37	3.88 (88)	0.5	0.5	1bkr

*Protein length.

**[Å (#residues)].

***Template Modeling score [0.0–1.0].

****True positives for Nc = 50 [0.0–1.0].

## Results

### Global better than local model for residue couplings

#### Mutual information does not sufficiently correlate with residue proximity

**Figure 3:**
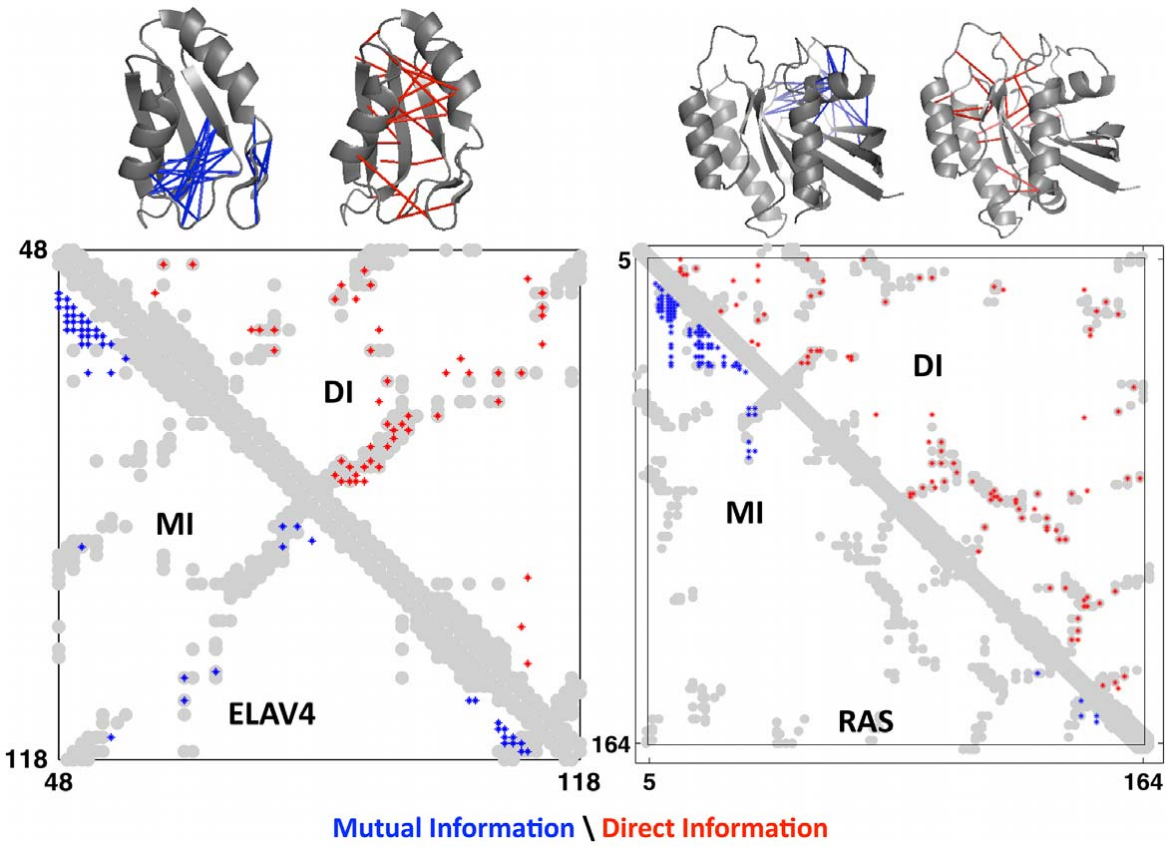
Progress in contact prediction using the maximum entropy method. Extraction of evolutionary information about residue coupling and predicted contacts from multiple sequence alignments works much better using the global statistical model (right, Direct Information, DI, Equation 3) than the local statistical model (left, Mutual Information, MI, Equation 1). Predicted contacts for DI (red lines connecting the residues predicted to be coupled from sequence information) are better positioned in the experimentally observed structure (grey ribbon diagram), than those for MI (left, blue lines), shown here for the RAS protein (upper) and ELAV4 protein (lower). The DI residue pairs are also more evenly distributed along the chain and overlap more accurately with the contacts in the observed structure (red stars [predicted, grey circles [observed] in contact map; center, upper right triangle) than those using MI (blue [predicted], grey circles [observed]; center, lower left triangle). Details of contact maps for all proteins comparing predicted and observed contacts are in Figures S1 and S2, Text S1.

We first attempted the prediction of residue-residue proximity relationships using the straightforward local mutual information (MI) measure. *MI(i,j)* for each residue pair *i*, *j* is a difference entropy which compares the experimentally observed co-occurrence frequencies *f_ij_(A_i_,A_j_)* of amino-acid pairs *A_i_*, *A_j_* in positions *i*, *j* of the alignment to the distribution *f_i_(A_i_)f_j_(A_j_)* that has no residue pair couplings (details in Text S1):
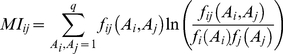
(1)Contact maps constructed from residue pairs assigned high *MI* values, and thus interpreted as predicted contacts, differ substantially from the correct contact maps deduced from native structures, consistent with the work of Fodor et al. [9] (Figure S1). Visual inspection of *MI*-predicted contacts as lines connecting residue pairs superimposed on the observed crystal structure confirms that the contacts predicted from *MI* are often incorrect and/or unevenly distributed (Figure 3, left, blue lines). Presumably this arises due to the local nature of *MI*, which is independently calculated for each residue pair *i*,*j*. Plausibly, the key confounding factor is the transitivity of pair correlations, where the simplest case involves residue triplets; for example, if residue B co-varies with both A and C, because B is spatially close to both A and C, then A and C may co-vary even without physical proximity (A–C is a transitive pair correlation). Any local measure of correlation, not just mutual information, is limited by this transitivity effect.

#### Effective residue couplings from a global maximum entropy model

To disentangle such direct and indirect correlation effects, we use a global statistical model to compute a set of direct residue couplings that best explains all pair correlations observed in the multiple sequence alignment (see Methods and Text S1) [15], [47]. More precisely, we seek a general model, *P(A_1…_A_L_)*, for the probability of a particular amino acid sequence *A_1…_A_L_* of length *L* to be a member of the iso-structural family under consideration, such that the implied probabilities *P_ij_(A_i_,A_j_)* for pair occurrences (marginals) are consistent with the data. In other words, we require *P_ij_(A_i_,A_j_)∼f_ij_(A_i_,A_j_)*, where *f_ij_(A_i_,A_j_)* are the observed pair frequencies of amino acids at positions *i* and *j* in the known sequences in the family and the marginals *P_ij_(A_i_,A_j_)* are calculated by summing *P(A_1…_A_L_)* over all amino acid types at all sequence positions other than *i* and *j*. As specification of residue pair properties (ignoring higher order terms) leaves the amino acid sequence underdetermined, there are many probability models that would be consistent with the observed pair frequencies. One can therefore impose an additional condition, the maximum entropy condition, which requires a maximally even distribution of the probabilities - while still requiring consistency with data. Probability distributions that are solutions of this constrained optimization problem are of the form [11], [45], [49]:
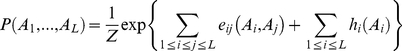
(2)Here *A_i_* and *A_j_* are particular amino acids at sequence positions *i* and *j*, and *Z* is the normalization constant. The Lagrange multipliers *e_ij_(A_i_,A_j_)* and *h_i_(A_i_)* constrain the agreement of the probability model with pair and single residue occurrences, respectively. This global statistical model is analogous to statistical physics expressions for the probability of the configuration of a multiple particle system, such as in the Ising or Potts models. In this analogy, a sequence position *i* corresponds to a particle, such as a spin, and can be in one of 21 states (*A_i_ = 1..21*); and, the Hamiltonian (the expression in curly brackets) consists of a sum of particle-particle coupling energies *e_ij_(A_i_,A_j_)* and single particle coupling energies to external fields *h_i_(A_i_)*.

For our protein sequence problem, the *e_ij_(A_i_,A_j_)* in equation 2 are essential residue couplings that are used in the prediction of folding constraints and the *h_i_(A_i_)* are single residue terms that reflect consistency with observed single residue frequencies. These parameters are thus optimal with respect to the two key conditions, (1) consistency with observed data (pair and single residue frequencies) and (2) maximum entropy of the global probability over the set of all possible sequences. In practice, once these parameters are determined by matrix inversion (Equations M4, M5), one can directly compute the effective pair probabilities *P_ij_^Dir^(A_i_,A_j_)* (Equation M6), and from these the effective residue couplings (‘direct information’, in analogy to the term ‘mutual information’) *DI_ij_* by summing over all possible amino acid pairs *A_i_*,*A_j_* at positions *i*,*j*:
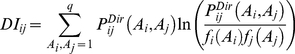
(3)The crucial difference between this expression for direct information *DI_ij_* (Equation 3) and the equation for mutual information *MI_ij_* (Equation 1) is to replace pair probabilities estimated based on local frequency counts *f_ij_(A_i_,A_j_)*, by the doubly constrained pair probabilities *P_ij_^Dir^(A_i_,A_j_)*, which are globally consistent over all pairs *i*,*j*.

#### Global maximum entropy statistical model reveals residue proximity

We now examine whether the residue coupling scores *DI_ij_* (Equation 3; Equation 22, Text S1) from the maximum entropy model provide information about spatial proximity. Are residue pairs with higher *DI_ij_* scores more likely to be close to each other in 3D structure? Examination of contact maps displaying residue pairs with highly ranked *DI_ij_* values, overlaid onto contact maps for an observed (crystal) structure, reveals a surprisingly accurate match. The high-scoring residue pairs are often close in the observed structure, and these pairs are well distributed throughout the protein sequence and structure, in contrast to pairs with high-scoring *MI_ij_* values, (Figure 3, Figure S2). This remarkable level of correct contact prediction holds for all of our test cases (Table 1, Table S1) in the four main fold classes.

Others have shown that given sufficient correct (true positive) contacts combined with a lack of incorrect (false positive) contacts, predicted contacts can be implemented as residue-residue distance restraints to fold proteins from the main four fold categories with up to ∼200 residues to under 3 Å C_α_-RMSD error from the crystal structure [50] and, in later work, up to 365 residues with accuracy under 3 Å C_α_-RMSD error [50], [51]. We were therefore encouraged to use our blindly predicted proximity relations as residue-residue distance restraints to fold proteins *de novo* from extended polypeptide chains.

### Protein all-atom structures inferred from evolutionary constraints

In spite of elegant analyses using subsets of real contacts [50], [51], it is not *a priori* obvious to what extent accuracy of contact prediction translates to accuracy of 3D structure prediction and, in particular, how robust such prediction is to the presence of false positives. We therefore decided to assess the accuracy of contact prediction by the very stringent criterion of accuracy of predicted 3D structures.

#### Generating model structures

Starting from an extended polypeptide chain with the amino acid sequence of a protein from the family (Table S1) we used well-established distance geometry algorithms, as used for structure determination by nuclear magnetic resonance (NMR) spectroscopy [52] (Text S1). The distance constraints were constructed using residue pairs with high DI scores pairs and secondary structure constraints predicted from sequence (Text S1, Appendix A1, Table S2). The protocol generates initial 3D conformations and then applies simulated annealing [48] (steps outlined in Text S1 and Appendix A2). We reasoned that the number of distance constraints (*N_C_*) needed should scale monotonically with the protein length *L*, as seen in fold reconstruction from observed contact maps [50], [51]. To explore the variability of predicted structure using a given set of distance restraints, we generated 20 candidate structures for a range of *N_C_* values which started at *N_C_* = 30 and incremented in steps of 10 to the nearest multiple of 10 to *L*, e.g., from *N_C_ = 30* to *N_C_ = 160* for the Hras proteins which has 160 core residues in the PFAM alignment. Thus, in total we generate on the order of 2*L candidate three-dimensional structures for each protein family as prediction candidates, more precisely, between 400 and 560, depending on the size of the protein (Table 1, Appendix A3). In practice, a smaller number of candidate structures may be sufficient. Each candidate is an all-atom structure prediction for a particular reference protein of interest chosen from the family. The model structures satisfy a maximal fraction of the predicted distance constraints and meet the conditions of good stereochemistry and consistency with non-bonded intermolecular potentials. The top predicted structure for each protein is selected by blind ranking of these candidate structures using objective, primarily geometric, criteria (Figure 2, Figure S2, Appendix A3).

### 3D structure inference for small and larger proteins of diverse fold types

To evaluate the information content of residue pair correlations with respect to protein fold prediction, we apply the method to increasingly difficult cases. We start with small single-domain proteins and move on to larger, more difficult targets, eventually covering a set of well-studied protein domains of wide-ranging biological interest, from different fold classes. We report detailed results for four example families, and summary results for 11 further test families, and provide detailed 3D views of all 15 test protein families in Figure S3 and detailed 3D coordinates and Pymol session files for interactive inspection in Appendices A3 and A4, http://cbio.mskcc.org/foldingproteins.

#### Small: an RNA binding domain (RRM)

The blind prediction of the 71-residue RRM domain of the human Elav4 protein (Uniprot ID: Elav4_human) is a typical example of a smaller protein. The distance constraints are derived from a rich corpus of 25K example proteins in the PFAM family. The highest ranking predicted structure has a (excellent) low 2.9 Å C_α_ -RMSD deviation from the crystal structure over 67 out of 71 residues, a TM score of 0.57 and GDT_TS 54.6, indicating overall good structural similarity to the observed crystal structure, [53], [54], (Figure 2 top, Table 1). It has correct topography of the five β-strands and two α-helices, marred only by a missing H-bond pattern between strands 1 and 3, at least partly due to the truncation of the strand 1, a consequence of the short length of the sequence in the PFAM alignment. Strands 2 and 3 align with only 1.6 Å C_α_-RMSD deviation over the length of the predicted strands and are positioned well enough for hydrogen bonding, with some correct registration. Interestingly, the 4^th^ β-strand (penultimate) missed by the secondary structure prediction method is placed in the correct region in 3D: this is one of several examples in which residue coupling information overrides incorrect local prediction. The predicted top-ranked domain of Elav4 very likely lies within the refinement basin of the native structure.

#### Medium size: Ras oncogene (G-domain), an α/β domain with an GTPase active site

The G-domain family in PFAM, with Human Ras proto-oncogene protein (Uniprot-ID: hras_human) chosen as the protein of interest, has a core multiple sequence alignment (MSA) of 161 residues. The structure has an α/β fold with a 6-stranded β-sheet, surrounded by 5 α-helices, one of which (α-2) is involved in the GTPase switch transition after GTP hydrolysis. The highest ranked, blindly predicted structure is 3.6 Å C_α_-RMSD from the crystal structure, over 161 residues (Figure 2 middle) and has a high TM score of 0.7 (range 0.0–1.0, with 1.0 implying 100% of residues are within a set distance from the correct position [53]). The six β-strands and five α-helices are placed in the correct spatial positions and are correctly threaded (Appendices A3 and A4). The 6 β-strands, which make 5 β-strand pairs are not within hydrogen boding distance for all backbone bonding, but the correct register can be easily predicted for 26/30 of the residue pairs, Text S1. The accuracy of overall topography of the highest-ranked structures is remarkable (Table 1) and, as far as we know, currently not achievable for proteins of this size by any *de novo* structure prediction method [27].

#### Larger: trypsin, an enzyme with a two-domain β-barrel structure

The largest (non-membrane) protein family tested in the blind test is the trypsin-fold serine protease family, with rat trypsin chosen as a representative protein. Its size, at 223 amino acids, is significantly larger than proteins that can be predicted by other de novo computational methods. Trypsin consists of β-strands in two structurally isomorphous β-barrel domains. The highest-ranked predicted structure has 4.3 Å C_α_-RMSD error over 186 out of 223 residues (Figure 2 bottom, Table 1, Appendices A3 and A4). The overall distribution of secondary structure elements in space is approximately correct and our method correctly predicts 5 disulfide bonded cysteine pairs, which lie within our alignment, Text S1. The topography of the first β-barrel (domain 1) is good and plausibly within refinement range of the observed structure. Five correct pairs of β-strands are identified (one absent) and 70% of hydrogen bonding paired residues are predicted with correct register, Text S1. However, domain 2 has a number of incorrect loop progressions (see Pymol session in Appendix A3), and possibly (by inspection) is not within refinement range of the correct structure. Predicting the structure of proteins in the trypsin family is particularly challenging, as the structure is known to undergo a conformational change after cleavage of the activation peptide [55] and, as the N-terminal and C-terminal peptide cross from one domain to the other.

#### Inferring the residue configuration in the active site of trypsin

In spite of the limited quality of structure prediction in domain 2 of trypsin, it is interesting that the top-ranked structures place the C_α_ atoms of the highly conserved active site triad residues Ser-His-Asp in correct *relative* spatial proximity, i.e., within 0.64 3 Å C_α_-RMSD (and 1.3 Å all atom-RMSD) error, after superimposition of the three residues of the catalytic site with the same three residues of the experimental structure (Figure S4). This may reflect strong evolutionary constraints near functional sites and may imply that the configuration of resides around an active site can be predicted more accurately than other detailed aspects of the 3D structure. The ability to predict active site constellations at this level of accuracy would be particularly interesting for the design of drugs on predicted structural templates.

#### Exploration: rhodopsin, an α-helical transmembrane protein

Rhodopsin is the first membrane protein predicted using this method. This important class of membrane proteins has 7 helices and the PFAM family from which the distance restraints are inferred contains many subfamilies of class A G-protein coupled receptors [56]. For the highest ranked predicted rhodopsin structure (4.84 Å C_α_-RMSD error from a representative crystal structure over 171 residues), the overall topography of the helices is accurate (TM score 0.5), with most of the positional deviation arising for helices 1 and 7, which are misaligned relative to the direction perpendicular to the membrane surface, (Table 1, Figure S3). The predicted structure with the highest TM score (0.55), and 4.29 Å C_α_-RMSD over 180 residues, also misaligns the terminal helices but does recapitulate a network of close distances (<4.5 Å) between the side chains of Arg135 (helix III) and Glu247, Thr251 (helix VI) as well as other well-known inter-helical proximities such as Asn78 (helix II) to Trp161 (helix IV) and Ser127 (helix III) [57]. Given that the current version of the method has no information about membrane orientation for membrane proteins, this constitutes an excellent starting point for future application of the method to 3D structure prediction for membrane proteins.

#### Ranking inferred structures

To arrive at useful and objective blind predictions, the set of inferred structures for each family is ranked by objective criteria based on physical principles and a priori knowledge of general principles of protein structure. In the current implementation, we use consistency with the well-established empirical observation of right-handed chain twist in α-helices and right-handed inter-strand twist for β-strand pairs [58] (Text S1). The virtual dihedrals of the α-helices and the predicted β-twists in the candidate structures were combined together as a score, weighted by the relative numbers of residues in β-strands and α-helices for each protein, see scores for all structures in Appendix A5. We found these geometric criteria effective in eliminating artifacts that appear to arise from the fact that distance constraints do not have any chiral information, such that the starting structures prior to refinement using molecular dynamics, while consistent with distance constraints, may have incorrect chirality, either globally or locally. We also eliminated candidate structures with knots (as with the top ranked trypsin prediction) according to the method of Mirny et al. [59].

**Figure 4:**
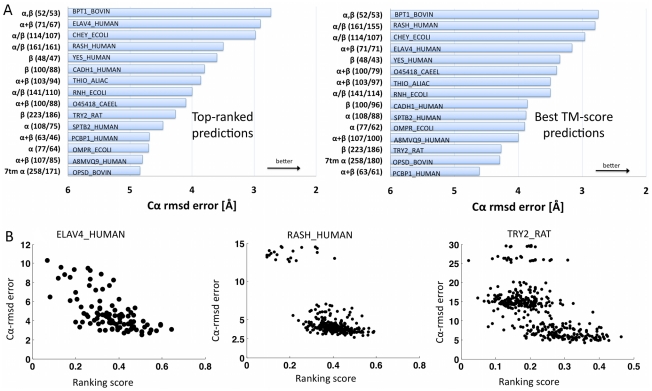
Accuracy of blinded 3D structure inference. **A.** The overall performance of the de novo structure prediction reported here based on contacts inferred from evolutionary information (EICs), ranges from good to excellent for the 15 test proteins (on left: 3D structure type [α = α-helix-containing, β = β-strand-containing, 7tm-α = containing seven trans-membrane helices]; in parentheses: size of protein domain/number of residues used for C_α_-RMSD error calculation; on bar: Uniprot database ID). Larger bars mean better performance, i.e., lower C_α_-RMSD co-ordinate error. Left: performance for the top ranked structure for each target protein out of 400–560 (depending on the size of the protein, 20 structures per *N_C_* bin, *N_C_* in steps of 10, details in Appendix A3 and A6) candidate structures in blind prediction mode; right: performance of the best structure, in hindsight, out of 20 candidate structures generated, for 20 sets of constraints ranging from 10∶200, in steps of 10. This reflects what would be achievable with better ranking criteria or independent post-prediction validation of structure quality (Table 1; details of blind ranking scores in Web Appendix A5). Other well-accepted methods for error assessment, such as GDT-TS and TM score are useful for comparison purposes (Table S1, Web Appendix A6). **B.** Ranking score of each candidate structure (quantifying expected structure quality) versus C_α_-RMSD error. Ideally, higher-ranking scores correspond to lower error. The distribution of the candidate structures (black dots) for Elav4, Ras and Trypsin shows, in retrospect, that the ranking criteria used here are relatively useful and help in anticipating which structures are likely to be best (plots for all tested proteins in Figure S5). In blind prediction mode, a list of predicted candidate 3D structure has to be ranked by objective and automated criteria, with a single top ranked structure or a set of top ranked structures nominated as preferred predictions.

The highest-ranked all-atom model structure is taken as the top blindly predicted structure (Table 1, Table S1). Lower ranked structures are expected to have lower accuracy of 3D structure, but this has to be tested after blind prediction by comparison with known structures. As a test of the entire procedure and the ranking criteria, we assessed our blind predictions by comparing the ranking score of the predicted structures with the experimentally observed structure, from X-ray crystallography, of the chosen reference protein, (Text S1, Figure 4A, Figure S5 and Appendix A5). For proteins such as RAS and Trypsin (Figure 4B), the objective criteria successfully ranks those predicted structures with the lowest C_α_-RMSD error to a crystal structure as highest scoring. As we remove obviously knotted proteins [59] we would miss genuinely knotted proteins [60] which are, however, rarely observed.

### Assessment of prediction accuracy: 3D structures

#### Summary of blinded 3D accuracy for 15 test proteins of known structure

We were surprised at the extent and high value of the information in the derived distance constraints about the 3D fold of examples from all major fold classes containing various proportions of α-helices and β-sheets. This high information content in residue couplings, derived from the maximum entropy statistical model, extends, so far, to proteins as large as G-domains, like H-ras, with 161 residues, and serine proteases, like trypsin, with 223 residues, as well as the rhodopsin family, a trans-membrane protein, with 258 aligned residues. This size has so far been out of range for state-of-the-art *de novo* prediction methods even when three-dimensional fragments are used [22], [61]. In general we find that predicted α/β folds, among the 15 proteins investigated in detail, produce the most accurate overall topography (Table 1, Table S1, Figure S5.). We anticipate that these results will likely extend to many protein families and that accurate structures can be generated for many of these using distance constraints derived from evolutionary information and predicted secondary structure alone, followed by energy refinement. For 12 out of the set of 15 protein families (Table 1), the top blindly ranked structures have coordinate errors from 2.7 Å–4.8 Å for at least 75% of the residues, using the accepted practice of omitting a moderate fraction of badly fitting residues in order to avoid exaggerated influence from outliers resulting from the square in the definition of C_α_-RMSD (using the MaxCluster suite [62]). For most practical purposes, one might consider these to be within the basin of attraction within which one is highly likely to be able to identify the particular correct fold, which we estimate roughly to have a radius of about 5 Å C_α_-RMSD. The partial exceptions are rhodopsin (OPSD) for which the relatively low 4.8 Å C_α_-RMSD error is limited to 171 out of 258 residues (66%); and PCBP1 at 4.7 Å for 46/63 residues (73%). For these proteins, the agreement is limited to a smaller, though still sizable, fraction of the protein and it is less likely that the correct overall fold would be recognized. The major exception is SPTB2 at 4.0 Å for 47/108 residues (44%), which we consider not satisfactory. The TM scores customary in CASP reflect these differences and it is plausible that the top-ranked predictions for 11 out of the 15 test proteins would be considered excellent for de novo modeled structures of this size (Table S1) [27], [61], [63].

**Figure 5:**
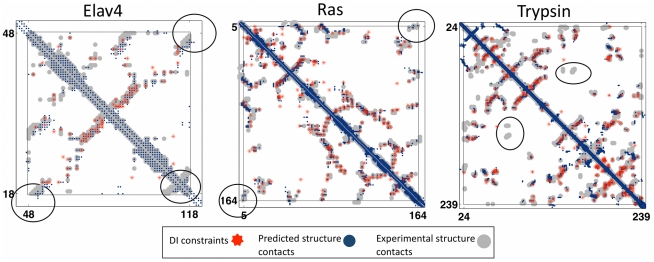
Top-ranked predicted structures can make correct contacts in the absence of constraints and avoid incorrect contacts in spite of false positive constraints. The top blindly ranked structures are evaluated in terms of quality of contact prediction (N_C_ = 40 for Elav4, N_C_ = 130 for Ras, N_C_ = 160 for Trypsin). The predicted constraints (red stars) are correct when they coincide with contacts derived from the observed structure (grey circles) and otherwise incorrect (false positives, red on white). The contacts derived from the predicted 3D structure (dark blue) are in good general agreement with those from the observed structure (grey). The cooperative nature of the folding prediction process permits favorable situations, in which contacts regions not touched by a predicted constraint (red) are still predicted correctly (black circle for RAS, dark blue on grey, no red) and false positive constraints are not strong enough to lead to incorrect contacts (left black circle Elav4, red star, no dark blue or grey). However, in unfavorable situations missing constraints may imply that contact regions are fully or partially missed (black circle, trypsin) or mostly missed (right black circle for Elav4, grey adjacent to and wider than dark blue).

Detailed examination of the close contacts of top ranked predicted structures reveals interesting violations, (Figure 5). For Ras and Trypsin false positive DI constraints (between Ser145 and Asp57 for Ras, and Ser127 and Ala37 for trypsin) are not satisfied in the top predicted structures thereby improving the accuracy. Conversely, a contact is made the N-terminal β-strand and the C-terminal helix in RAS and C-terminal β-strand in ELAV4, despite the fact that no constraints are used in the vicinity of these contacts (grey circles, Figure 5).

#### Best 3D prediction accuracy in top 400 candidate structures

To assess the potential of the method and with a view toward future improvements of ranking criteria for sets of candidate structures, one can ask the question, from hindsight, which of, say, 400 candidate structure has the highest accuracy. This question is analogous to protein structure prediction reports that discuss the relationship (scatter plots) of, e.g., model energy against model error. Here, the best candidate structures by TM score, selected from among 400 candidate structures for each protein (*N_C_* = 10–200), have TM scores from 0.5 to 0.76 and typically a lower error than the blindly top ranked structure, ranging from 2.8 Å to 4.6 Å C_α_-RMSD for all 15 families, covering at least 80% of the residues, with the exception of OPSD where we achieve 4.3 Å for 180/258 residues (66%), (Figure 4B, Table1, Table S1). The fact that in most cases better 3D structures are found in the top 400 candidates is a non-trivial positive indication, as the conformational search space of protein folds is so large, that random methods, or moderately effective methods, would have an exceedingly low probability of achieving errors in this low range in as few as 400 structures. However, some of the structures generated here among the top 400 appear topologically incorrect, with the polypeptide chain passing through loops in a way that is, according to visual intuition, atypical of fully correct structures. Such topologically incorrectly structures would not be within a basin of attraction of conventional energy refinement, e.g., by simulated annealing. This indicates that neither low C_α_-RMSD as a measure of overall accuracy, nor the more recently developed template modeling (TM) score, nor the global distance test - total score (GDT-TS), is fully informative indicators of structure quality. These classic structure comparison metrics need to be supplemented by more sophisticated measures, which quantify topographical differences in chain progression in 3D space, a direction for future work [64], [65], together with an analysis of violations of constraints in the spirit of Miller et al. [3]. In any case, the encouragingly high accuracy of the folds we generate amongst a relatively small number of candidates imply that improved ranking criteria may lead to a better set of top-ranked, fully blinded predictions.

#### Current technical limits of 3D prediction accuracy

As an estimate of the accuracy maximally achievable by this method and its particular implementation, we performed reference calculations using artificial, fully correct, distance constraints derived from the experimentally observed structure. With this ideal set of constraints, we can construct protein structure models at an error of not lower than about 2.0 Å C_α_-RMSD (Text S1, Table S3, larger values for some of the larger proteins). This places a lower bound on the expected error, inherent in the distance geometry and refinement part of the method and this error will scale to some extent with the length of the protein as others have noted [50]. That we achieve candidate structures close to these bounds with predicted distance constraints is consistent with the notion that the inferred residue couplings contain almost all the information required to find the native protein structure, at least for the 15 protein families examined here. This technical lower limit also represents a challenge for generic methods improvement for computation of accurate all-atom structures from distance constraints.

### Assessment of prediction accuracy

#### Accuracy of contact prediction

The accuracy of prediction of 3D structures crucially depends on the accuracy of contact prediction and the choice of distance constraints from a set of predicted contacts. Note that residue-residue proximity is a different requirement than residue-residue contact, as residues may be near each other in space without any of their atoms, being in inter-atomic contact (defined as inter-atomic distance near the minimum of non-bonded inter-atomic potentials (‘van der Waals’), say, about 3.5 Å). Here, we use the term inter-residue contact interchangeably with inter-residue proximity, i.e. minimum atom distance of less than 5 Angstroms. We assess the accuracy of contact prediction in terms of the number of true positives and false positives among predicted contacts, i.e., those that agree and those that disagree with the contacts observed in known 3D protein structures.

**Figure 6:**
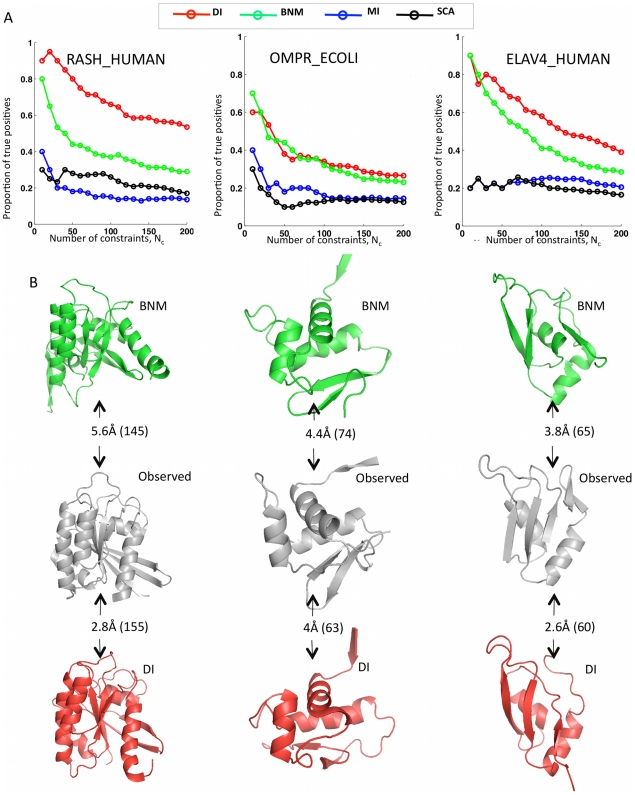
Key requirement of global statistical model for correct prediction. Evaluation of accuracy in terms of predicted contacts (A) and predicted 3D structures (B). (A) The two global models, the Bayesian network model (BNM, green [13]) and direct information model (DI, red, this work and [47]) have a consistently high rate of correctly predicted contacts (true positives) among the top NC ranked residue pairs; two local models, mutual information (MI, green, equation 1) and SCA (black, [66]) have a consistently lower rate of true positives. Here, local refers to statistical independence of each pair i,j, while global refers to statistical consistency of all pairs. In (B), only the predicted 3D structures (green, BNM; red, EIC) for the global models agree well with the observed structure (grey); Cα-RMSDs is calculated over the number or residues in parentheses (Pymol sessions for all structures in Web Appendix A4). Attempts to generate 3D structures for the two local methods MI and SCA failed (not shown). Comparing (A) and (B) confirms that a higher rate of true positives for contact prediction leads to better 3D structures and that for DI one needs at least a true positive rate of about 0.5 for about 100 predicted contacts, depending on size and other details of particular protein families. Interestingly, a false positive rate as high as about 0.3–0.5 can still be consistent with good 3D structure prediction.

We find that the highest scoring pairs provide remarkably accurate information about residue-residue proximity (Figure 6A, Figures S6 and S7). For example, the rate of true positives is above 0.8 for the first 50 pairs for HRAS and still above 0.5 for the first 200 pairs; for other proteins, it is lower but still relatively high, e.g., above 0.7 and 0.4 for the first 50 and 200 for ELAV4. These results are consistent with our parallel evaluation of contact prediction accuracy for a large number of bacterial protein domains [47] and represent a significant improvement over local methods of contact prediction from correlated mutations or co-evolution. Not surprisingly, there is a general trend for a higher rate of true positive contact prediction to results in better predicted 3D structures, The predicted structures of proteins such as Ras and CheY with a high proportion of true positive predicted contacts tend to be more accurate than those with lower rates, for example the KH domain of PCBP1 and the calponin homology domain of SPTB2. However, this relationship between the proportion of true positives and the accuracy of the best-predicted structures is not as simple as one might have expected, Figures S6, S8 and S9. For instance the thioredoxin predicted structures are on the whole more accurate than the predicted the lectin domain (A8MVQ9_HUMAN) structures despite the fact that thioredoxin has a lower true positive rate than lectin domain for its predicted contacts. Since the quality of 3D structures could depend also on the distribution of the contacts through the chain, for each protein we also calculated the distance of a experimental contact to the nearest predicted contact and this ‘spread’ showed a good correlation with the C_α_-RMSD accuracy achieved, (Figure S10 and Text S1).

#### Comparison of contact prediction accuracy between global and local models

How well do other contact prediction methods work? The two global models, the Bayesian Network Model (BNM, [13], [46]) and the DI model (this work and [15] have a consistently high rate of correctly predicted contacts (true positive rate) among the top *N_C_* ranked residue pairs; in comparison two local models, MI (Equation 1) and statistical coupling analysis (SCA, [66]), both have a lower rate of true positives (Figure 6A, Figures S6, S7, S11, S12, S13, S14, and S15). The relatively high accuracy of contact prediction in the BNM model encouraged us to generate predicted 3D structures based on the BNM ranked residue pairs as the basis for inferred distance constraints, following the protocol developed for the DI model. For ten test proteins, folded all-atom 3D structures for BNM agree well with the observed structure (green structures in Figure 6B and data not shown). On the whole, the C_α_-RMSD errors are somewhat higher for the structures from the BNM model than those for the DI model (red structures in Figure 6B). In particular, using the notation [protein identifier/error for BNM/error for DI], we have: [RASH/5.6 Å/2.8 Å], [ELAV4/3.8 Å/2.6 Å], [YES/4.6 Å/3.6 Å] [CADH/4.7 Å/3.9 Å] and trypsin did not reach an accuracy lower than 12 Å C_α_-RMSD with the BNM constraints (Figure 6B and data not shown). On the other hand, the BNM and the DI predictions for OMPR were in the same accuracy range when compared to the experimental structure, as the BNM result was over 74 atoms as opposed to 63 atoms for the DI method [OMPR/4.4 Å/4.0 Å].

These results confirm that in general a higher rate of true positives for contact prediction leads to better 3D structure prediction; and, that for the global methods one needs at least a true positive rate of about 0.5 and on the order of about 100 predicted contacts, depending on size and other details of particular protein families. Interestingly, a false positive rate as high as about 0.3–0.5 can still be consistent with good 3D structure prediction. Clearly, the global statistical models provide a substantial increase in the accuracy of prediction of residue contacts and of 3D structures.

### Information requirements for improved prediction of 3D structures

#### Requirement of sufficient sequence range coverage by the multiple sequence alignment

Among the test set of twelve protein families, the lowest accuracy was obtained for the SPBT2 and rhodopsin proteins, (see Table 1, Table S1, Figure S3). In these cases a significant number of key residues are not included in the PFAM hidden Markov model (HMM) and thus were excluded from our analysis. If the alignment covers only part of the structure, the statistical model of the sequence is restricted to this part of the structure and does not provide information for non-covered regions. Since regions not covered by the PFAM alignments are often at the N-terminus or C-terminus of the protein and these are in contact in many protein structures, this will significantly harm the accuracy of prediction that is possible. Our analysis also shows that prediction is less likely to be accurate even within the covered region when ends of the alignment are absent. How much additional sequence information is required to build an alignment for the entire protein sequence in each case? This question is non-trivial as the diversity sampled at each sequence position by evolution varies greatly. Indeed the strength of structural evolutionary constraints may diminish towards the protein termini, analogous to the ‘frayed ends’ observed in many NMR-determined structures.

#### Correct folding with a surprisingly small number of distance constraints

**Figure 7:**
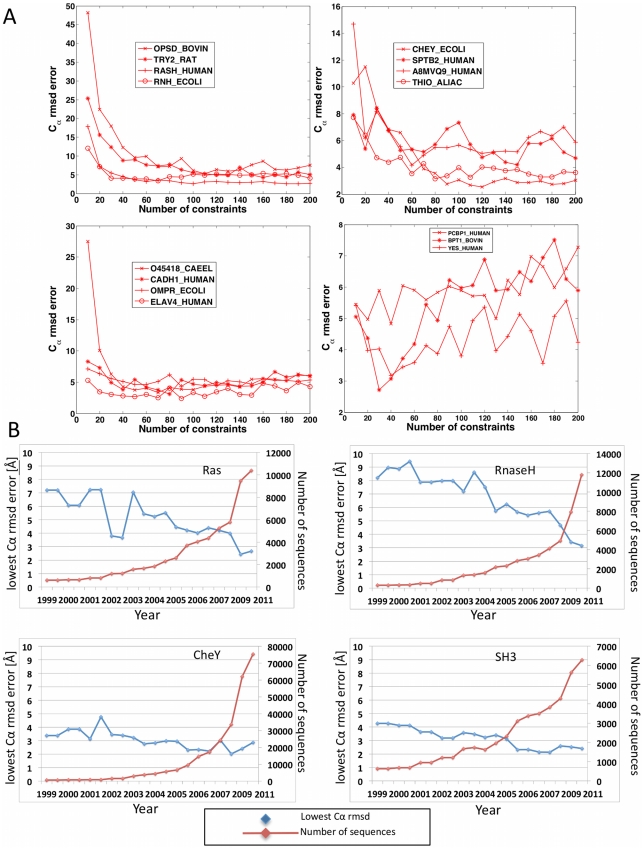
Moderate number of distance constraints and varying number of sequences required for correct 3D structure prediction. **A. How many distance constraints are needed for fold prediction?** What fraction of false positives can be tolerated? With increasing number of predicted essential distance constraints (N_C_, horizontal axis), 3D prediction error decreases rapidly, as assessed by C_α_-RMSD between the best of 20 (in each N_C_ bin) predicted structures and the observed structure (here, for the 15 test proteins, using Pymol). Remarkably, as few as ∼N_RES_/2 (∼L/2) distance constraints *d_ij_* (with chain distance |*i−j*|>5) suffice for good quality predictions below 5 Å C_α_-RMSD, where *N_RES_* is the number of amino acid residues in the protein multiple sequence alignment. We therefore routinely generated candidate protein structures for up to N_C_ = N_RES_ distance constraints for blinded ranking (and for up to N_C_ = 200 for other tests). Eventually the number of false positives does degrade prediction quality, e.g., for the 58 residue protein BPTI once N_C_ is about 80 (1.5 NRES) the prediction quality is lost. In practice, we do not recommend using N_C_>N_RES_, i.e, more than about one constraint *d_ij_* with |*i−j*|>5, per residue. **B. When would it have been possible to fold from sequence?** The increase in the number of sequences available in public databases (here, from successive archival releases of the PFAM collection of protein family alignments) is one of two key elements in the ability to predict protein folds from correlated mutations. Nevertheless plotting the numbers of sequences and dates shows that it would have been possible to calculate the structures up to 10 years ago for some proteins and that amazingly few sequences are sufficient. For example, although the retrospective prediction error (vertical axis, C_α_-RMSD, using Pymol) for the best 3D structure (of 400 candidates each) in four protein families (Ras, SH3 domain (YES_human) and RnaseH from Ecoli) has decreased over time, the decrease is not strictly monotonic, as the result of non-systematic growth of the database. The point at which a predicted protein structure from a particular family reaches below 4 Å Cα-RMSD varies considerably. For example, while RnaseH required about 6000 sequence to dip below 4 Å error, reached around 2008, the structure of CheY could have been predicted to 3.3 Å C_α_-RMSD, with only the 600 sequences available in 1999.

What is the minimum number of predicted distance constraints needed to generate an approximate 3D fold? An important parameter of our folding protocol is the number of inferred distance constraints, N_C_, used to generate candidate structures. While residues with the highest ranked pair correlations are usually close in 3D structure (Figures S6 and S7) the reliability decreases with decreasing value of *DI_ij_*. We assessed the accuracy of the predicted protein folds for 15 evaluation families as a function of N_C_ (Figures 7A and S16, Table S1).

Going from 10 to typically 200 distance constraints, we find that the prediction error drops sharply as EIC constraints are added, until false positives gradually start to degrade the prediction quality. We conclude that one needs about 0.5 to 0.75 predicted constraints per residue, or about 25–35% of the total number of contacts, to achieve reasonable 3D structure prediction. This number is close to those reported by other groups, who used fully correct close residue pairs to impose inexact distances as constraints [50], [51], [67]. For instance, Elav4 (length 71) folds to below 5 Å C_α_-RMSD with only 20 constraints, whilst Trypsin (length 223) takes 130 constraints. However, the number of constraints per residue to reach below 5 Å C_α_-RMSD is not constant (column 15 Table S1), and proteins such as OMPR at 0.66 constraints per residue, and Ras at 0.25 constraints per residue show that this will depend on other factors, such as type of fold and false positive rates. While the accuracy of structure prediction for some proteins clearly decreases as the number of false positives, for example Cadh1, Elav4 and Yes, other proteins, such as Ras and CheY stay the same or even improve in accuracy as the false positive proportion increases, (Figure S8). This result underlines the necessity of using the constraints to attempt to fold the proteins, in order to assay the quality of predicted contacts, rather than relying on true positive rates of contact prediction alone.

#### Increasing prediction accuracy over time, but lower than expected numbers of sequences needed

Since we not require today's standard of high performance computing, we wondered how long ago it would have been possible to make good structural predictions. How does the accuracy of predicted folds depend on the number of sequences in the multiple sequence alignment and their evolutionary diversity? To start to explore these questions we computed the accuracy of folding using distance constraints for four representative proteins, using alignments from 20 different releases of PFAM [1] covering the last 13 years. For each multiple sequence alignment we calculated 20 structures for a range of constraints from 30–200, (Figure 7B). During this period the available sequence information has increased dramatically as the result of new sequencing technology and large-scale genome projects, so we examined the best structure attained as a function of the number of sequences. Although there is a clear overall trend for the C_α_-RMSD of predicted structures to drop monotonically as the number of sequences in the family increases (for example, RnaseH, 4 Å C_α_-RMSD threshold was reached in 2009 when the number of sequences reached 5000), not all protein families behave the same way. The predicted Ras structures reached under a 4 Å C_α_-RMSD in 2002 with as few as 1200 sequences, then, surprisingly, rose again as more sequences were included, to finally dip to 2.5 Å C_α_-RMSD in 2009. Similarly, although the predicted structures of CheY and the SH3 domain from the Yes protein improve with the number of sequences available, predicted structures had C_α_-RMSD in errors as low as 3.3 Å and 4.7 Å respectively in 1999, with ∼600 sequences for both. (Figure 7B). Most surprisingly, a predicted OMPR structure with an error under 5 Å C_α_-RMSD would have been possibly using as few as 170 sequences (1999 PFAM release).

Hence our results highlight the overall relationship of accuracy of the predicted fold to the number of sequences available. However, this relationship is not straightforward. The distribution of sequences in the sequence space of a particular family will doubtless have an effect. In our current implementation of the algorithm, sequences with over 70% residue identity to family neighbors are down-weighted (Text S1). Therefore the effective number of sequences used for the DI coupling calculation is far less than the size of the family. Approximately only 12–40% of sequences available in the family are actually used for the calculation (Table S1). This reduction in the effective number of sequences varies substantially between families, highlighting the different distributions over sequence space covered by individual families (column 18 in Table S1). We speculate that future work will improve our understanding of *which*, as well as *how many* sequences are optimal for contact inference from evolutionary information.

## Discussion

### Evolutionary constraints are determinants of 3D structure

Protein folding algorithms tend to focus on finding the global minimum of the free energy of the polypeptide chain by physical simulations or by a guided search in conformational space using empirical molecular potentials. In this work we test the ability of a set of evolutionarily derived distance constraints between pairs of residues to guide the search towards the correct structure. As found in the study on the collective behavior of neurons, described quantitatively by models that capture the observed pairwise correlations but assume no higher-order interactions [40], our results suggest that pairwise amino-acid co-evolution statistics contain sufficient information to find the native fold. In both cases, success is contingent on the fact that indirect correlations are, at least to some extent, removed from consideration, this is achieved through the maximum entropy methodology. In the case considered here it was not necessary to explicitly consider higher order couplings, which greatly reduced the complexity of the analysis. The fact that this simplification works at all may be as much a starting point for an exploration of our understanding of the evolution of proteins as it is a route to structure prediction.

### Advantage of global statistical models

**Figure 8:**
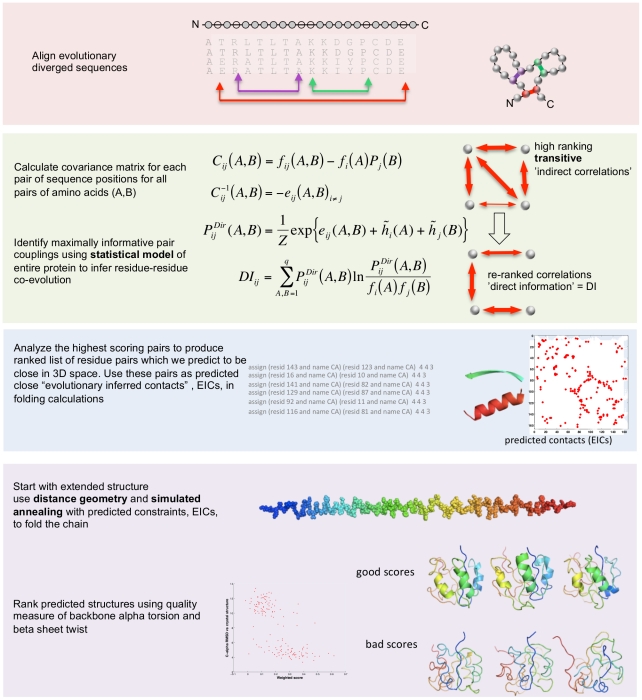
Computational pipeline for protein folding. The MSA for the protein family is typically generated by a sequence similarity search in a large database of protein sequences to collect related sequences that are likely to have similar 3D structures. Correlations between sequence positions *i* and *j* are calculated from observed frequencies of amino acids in single MSA columns and column pairs. By inferring a minimal statistical model of full length-sequences, which is consistent with these correlations (Text S1), direct coupling strengths *e_ij_(A,B)* between any pairs of residues are deduced. They help to derive distance constraints, which in turn are used to produce folded structures using the following steps: distance geometry generation of approximate folds, molecular dynamics simulated annealing using standard force fields, and chirality filtering. Here, we use MSAs from the PFAM collection of pre-aligned sequence families [1].

Our calculations show that the maximum entropy approach is very effective at taking into account the interdependencies of locally calculated mutual pair information. In contrast, MI high-ranking correlated residue pairs tend to be highly clustered in the contact map and have lower chain coverage, with substantial redundancy of information and a high rate of false positives from chain transitivity. In the maximum entropy calculation used to calculate the DI residue couplings, computation of the *C_ij_(Ai,Aj)* matrix is straightforward, given a multiple sequence alignment, however it is the matrix inversion (Equation 18a and b, Figure 8 and Text S1) that provides the global nature of the probability model. The application of this text-book approach from statistical physics to the problem of extracting essential pair couplings from alignments of protein sequences, with a 21-state model, leads to major progress in the problem of predicting protein-protein interactions from sequence data [11], and their use in protein folding (this work). Interestingly, an alternative approach to finding direct couplings using a Bayesian network model [BNM] also leads to improved accuracy of fold prediction using our folding protocol, compared to MI, but less so than DI couplings. A preliminary inspection showed that the overlap between the high-ranking couplings of the DI and BNM constraints is only about 40% yet the overlap contains an enhanced proportion of true positives. Understanding the theoretical connections between the two approaches may help combine the algorithms to improve the accuracy of the inferred contacts for deriving correct protein folds.

### Extracting proximity information for very conserved residues

Completely conserved residues provide no information about pair correlations, by definition. However, the ability to predict distance constraints between highly conserved residues is a valuable feature of the DI algorithm presented here, and, in contrast to other homology-free protocols, allows direct deduction of structural information about disulfide bonds and binding sites [24]. As described above the active site residues Ser, His, and Asp in Trypsin are accurate within 1.3 Å all atom RMSD of the crystal structure, (Figure S4). Even the four different loops that form the tri-nucleotide (GTP/GDP) binding site of HRAS protein, which contain well-known highly conserved amino acids boxes (GKS, DTAGQ, NKCD, SA in one-letter amino acid notation) separated by up to 100 residues in the sequence, appear in approximately the correct spatial location around the binding pocket in the highest ranking predicted structures. The striking accuracy of prediction of which loops participate in substrate sites formed by sequence-distant residues is consistent with strong evolutionary constraint in functional areas of the protein fold. The statistical model ranks co-variation signals from nearly conserved residues sufficiently highly to contribute to the correct prediction of such sites (Text S1).

### Limitations in prediction accuracy

Clearly some protein folds are predicted more accurately than others and this may be due to a number of different factors. One clear limitation in overall accuracy is structure generation from distance constraints, using any particular protocol, as demonstrated by the folds achieved from a control set of completely correct constraints. However, use of improved molecular dynamics approaches may lower the accuracy limits of our current pipeline and we anticipate refinement of the predicted structures using iterative approaches. Among our test set, some protein folds are predicted more accurately than others due to the quality of the predicted constraints – in particular the proportion of harmful false positives. As discussed earlier, possible reasons for false positive predictions of residue couplings include: (i) statistical background noise (e.g. low statistical resolution in the empirical correlations due to an insufficient number of proteins in the family or due to global correlations from phylogenetic bias in the frequency counts), (ii) the presence of functional constraints not involving spatially close residues, such as functional constraints imposed by protein-protein or protein-ligand interactions. In this work, we reduce the noise factor by requiring at least 1000 sequences in the protein family alignment, although one may be able to reduce this limit in the future with more refined methods for taking into account the density distribution of family members in protein sequence space, as well as the organization into protein subfamilies [68]. Functional constraints, for example resulting from interactions with external partners of the protein, or alternative conformations of the same protein as in allostery, are particularly interesting and will be the subject of future analysis.

### Contribution to the current art of 3D structure prediction

The challenge of 3D protein structure prediction depends on the extent of sequence similarity of the sequence of interest to other protein sequences whose structure is known. The difficulty of the prediction task ranges from fairly easy, if homologs of known structure are available, to very hard, when no detectable significant sequence similarity to a protein of known structure or to a known structural motif is available. Progress in this field has been expertly assessed by the pioneering community effort, the Critical Assessment of Techniques for Protein Structure Prediction (CASP), founded by Krzysztof Fidelis, John Moult and their colleagues in 1994 [69], [70], [71], www.predictioncenter.org). A series of ingenious methods have led to significant progress as reported in CASP since then, including threading, molecular dynamics, fragment-based assembly, contact prediction, machine learning, as well as methods combining several techniques [16], [63], [72], [73]. Internet servers have also facilitated the use of these new methods and allowed ongoing critical assessment of prediction accuracy [74], [75]. On this background, the goal of this work is to assess the contribution of one primary source of information, evolutionarily inferred residue couplings, to 3D structure, rather than optimizing prediction accuracy in the field of all other methods, as is done in CASP. We anticipate that in future objective assessment exercises others may want to adopt a derivative or variant of the method presented here for use in combination methods, e.g., improved contact energy in the I-Tasser simulation method [76] [19] or addition of EIC distance restraints into the Rosetta server. Here, the significant information content in inferred contacts is apparent both in the assessment of prediction accuracy both for contacts (2D) as well as for all-atom structures (3D).

### Contribution to solving biological problems

We anticipate that our method, alone or in combination with other techniques, may soon allow 3D structures with correct overall fold to be predicted for biologically interesting members of protein families of unknown structure, with potential applications in diverse areas of molecular biology. These include (1) more efficient experimental solution of protein structures by X-ray crystallography and NMR spectroscopy, e.g., by eliminating the need for heavy atom derivatives, by guiding the interpretation of electron density maps or by reducing the required number of experimental distance restraints, as elegantly demonstrated by the Baker and Montelione groups [77]. Additional interesting potential applications include (2) a survey of the arrangements of trans-membrane segments in membrane proteins; (3) discovery of remote evolutionary homologies by comparison of 3D structures beyond the power of sequence profiles [78] (4) prediction of the assembly of domain structures and protein complexes [79] (5) plausible structures for alternative splice forms of proteins; (6) functional alternative conformers in cases where our approach generates several distinct sets of solutions consistent with the entire set of derived constraints; and (7) generation of hypotheses of protein folding pathways if the DI predictions involve residue pairs strategically used along a set of folding trajectories. We also anticipate that structural genomics consortia would benefit greatly from reasonably accurate predictive methods for larger proteins, for example, to (8) prioritize protein targets and define domains of interest for both crystallography and NMR pipelines.

### The need to accelerate structure determination

Large investments continue in structural genomics, the global effort to solve at least one structure for each distinct protein family and to derive biological insight from these structures. While tremendous strides have been made in the last decade and experimental structure determination has been greatly accelerated, much less than 50% of the overall goal has been achieved to date. At the same time, the number of known protein families has increased as the result of massively parallel sequencing. Among the 12,000 well-organized protein domain families (PFAM-A collection of multiple sequence alignments), fewer than 6000 domain families have one member with a known 3D structure (from which plausible models can be built for all family members using the technique of model building by homology to structural templates). Beyond these, there are currently about 200,000 additional protein families with sequences that do not map to domains of known structure. The ability to calculate reasonably accurate structures for many of these families *de novo* from sequence information would enormously accelerate completion of the goal of structural genomics to cover the entire naturally occurring protein universe with known 3D structures. The speed advantage of the method under investigation here compared to experimental structure determination, derives from the increase of sequencing capacity by several orders of magnitude in the last decade. As we are about to reach a truly explosive phase of massively parallel sequencing, we anticipate increased coverage of sequence space for protein families by several orders of magnitude, well above the level of 1000–10000 non-redundant sequences for protein family and with rich evolutionary information about protein structure directly from sequence. We speculate that the utility of methods such as the one here has therefore not saturated, that predictions will become more accurate, and that applications will become broadly applicable to biological problems that can benefit from knowledge of protein structures.

### Protein folding in practice

Our *de novo* folding protocol for a medium-size protein using evolutionarily derived constraints does not require high-performance computing and can be done in well under an hour on a standard laptop computer. One starts with a multiple sequence alignment, uses the maximum entropy model to predict a set of residue couplings from the protein family alignment, adds predicted secondary structures, derives a set of distance constraints, generates initial structures using distance geometry, refines these using molecular dynamics with simulated annealing and ranks predicted structures according to a set of empirical criteria. This first detailed report for 15 proteins in different fold classes suggests that one can predict reasonably accurate protein structures “on the fly” and that one will be able to pre-compute and make publically available arguably useful predicted structures for thousands of protein families in diverse fold classes in the near future.

## Materials and Methods

The main steps (Figure 8) in the blind prediction (1) and subsequent evaluation (2) of accuracy are: (i) computation of effective direct coupling analysis (DCA) coupling strengths in the maximum entropy model, secondary structure prediction, definition of distance constraints inferred from evolutionary information (EICs), the number of constraints used and their relative weight, computation of a relatively small number of candidate structures, and development and application of automated criteria to rank predicted structures; and, (ii) evaluation of prediction accuracy by computation of structural error of predicted contacts and predicted 3D structures relative to the reference crystal structure. We call the overall method EVfold and additional details are available in Text S1, Table S4, S5 and http://EVfold.org.

### (1) Computation of DCA residue pair coupling parameters in the maximum entropy model

We identified a set of PFAM protein family sequence alignments with known crystal structure for at least one family member and more than 1000 sequences in each family. Sequences in the family alignments were weighted to reduce potential spurious correlations due to sampling bias from redundant sequence information in dense regions of sequence space. A maximum entropy model was applied to identify a maximally informative subset of correlated pairs of columns across the family alignment. The statistical model describes the expected behavior of all residues up to pair terms as a joint probability distribution.

To compute the effective pair couplings and single residue terms in the maximum entropy model two conditions must be satisfied. The first condition is maximal agreement between the expectation values of pair frequencies (marginals) from the probability model with the actually observed frequencies:
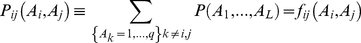
(M1)where *A_i_* and *A_j_* are particular amino acids sequence positions *i* and *j*. The second condition is maximum entropy of the global probability distribution, which ensures a maximally evenly distributed probability model and can be satisfied without violating the first condition:
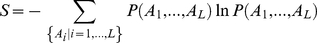
(M2)The solution of the constrained optimization problem defined by these conditions, using the formalism of Lagrange multipliers, is of the form:
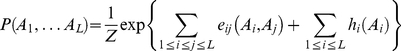
(M3)This global statistical model is formally similar to the statistical physics expression for the probability of the configuration of a multiple particle system, which is approximated in terms of a Hamiltonian that is a sum of pair interaction energies and single particle couplings to an external field. In this analogy, a sequence position *i* corresponds to a particle and can be in one of 21 states, and a pair of sequence positions *i*,*j* corresponds to a pair of interacting particles. The global probability for a particular member sequence in the iso-structural protein family under consideration is thus expressed in terms of residue couplings *e_ij_(A_i_,A_j_)* and single residue terms *h_i_(A_i_)*, where *Z* is a normalization constant.

Computationally, determination of the large number of parameters *e_ij_(A_i_, A_j_)* and *h_i_(A_i_)* that satisfy the given conditions is a complex task, which can be elegantly solved in a mean field approximation (Text S1 and [47]) or, alternatively, in a Gaussian approximation [80]. In either approximation the effective residue coupling are the result of a straightforward matrix inversion
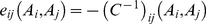
(M4)of the pair excess matrix restricted to (q−1) states (1≤*A*_i_,*B_j_*≤*q−*1) and
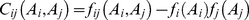
(M5)which contains the residue counts *f_ij_(A_i_,A_j_)* for pairs and *f_i_(A_i_)* for singlets in the multiple sequence alignment The parameters *h_i_(A_i_)* are computed from single residue compatibility conditions. Given the formulation of the probability model (Equation 1), the effective pair probabilities (with 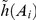 as defined in the Text S1) are
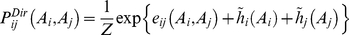
(M6)These pair probabilities refer to the full specification of particular residues *A_i_*, *A_j_* at positions *i* and *j*. For the quantification of effective correlation between two sequence positions *i* and *j*, one has to sum over all particular residue pairs *A_i_*,*A_j_* to arrive at a single number that assesses the extent of co-evolution for a pair of positions. In analogy to mutual information,
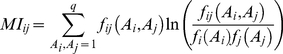
(M7)such that the DCA coupling terms between columns *i* and *j* are given by
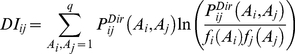
(M8)As there are *L^2^* values *DI_ij_*, and one expects residue contacts of the order of magnitude of *L*, only a relatively small number top-ranked *DI_ij_* values (ordered in decreasing order of numerical value) are useful predictors of residue contacts in the folded protein. Given the analogy to statistical physics, the residue couplings *e_ij_(A_i_, A_j_)*, on which the *DI_ij_* are based, can be thought of as pair interaction energies. The hypothesis, that only a fairly small subset of these pair terms are needed to determine the protein fold, is consistent with the very interesting physical notion that only subset of residue-residue interactions essentially determine the protein folding pathway. In practice, the validity of the probability formalism does not depend on the validity of this physical interpretation. We therefore proceed to use the ranked set of *DI_ij_* values as raw valuable material for the derivation of distance restraints for 3D structure prediction. The most computationally intensive step being inversion of a large matrix of pair terms, the *C_ij_(A,B)* matrix (over sequence positions *i = 1,L and j = 1,L*; and amino acid residue types *A = 1,20* and *B = 1,20*, of dimension L^2^ * 20^2^, with *L* the length of the sequence of order 50–250 residues in the current application.

### (2) Selection of EIC distance constraints for use in the generation of all-atom structures

The top-ranked set of *DI_ij_* are then translated to inferred contacts (EIC pairs) using four uniformly applied automated rules: consistency with predicted secondary structure, removal of predicted pairs close in sequence, exclusivity of SS bridge pairs and a conservation filter. These rules were derived from general plausibility arguments, and do not carry any information about the topography of particular folds or fold types (Methods). The first *N_C_* inferred EIC pairs, ranked according to their DCA coupling scores, are then translated to distance constraints, i.e., bounds on the distances between C_α_ and C_β_ residue and side chain centers between paired residues; and, as weighted distance restraints for structure refinement by simulated annealing using molecular dynamics, resulting in candidate all-atom protein domain structures.

### (3) Blinded structure prediction

The protein polymers are folded from a fully extended amino acid sequence of the protein of interest using standard distance geometry techniques and simulated annealing with standard bonded and non-bonded intra-molecular potentials (in vacuum) using the CNS molecular dynamics software suite, with a simulated annealing protocol similar to those used in structure determination from NMR [48]. The elimination of mirror topologies and ranking of candidate structures is achieved by computing virtual dihedral angles using four appropriate C_α_ atoms, reflecting standard α-helical and β-strand pair handedness, and then adding the scores normalized to the predicted secondary structure content (Text S1 and Figure S5). Candidate structures are also filtered to remove knotted structures as defined by computation of an Alexander polynomial by the KNOT server [59].

### (4) Evaluation of prediction accuracy

Accuracy of prediction of *residue-residue contacts* is quantified in 4 ways: (i) comparison of the EIC rank versus the minimum inter-residue distance in the crystal structure (Figure S7); (ii) comparison of the true positive rate of contact prediction versus the number of constraints (Figure S6); (iii) quantification of the severity of the false positives in a set of predicted constraints by measuring the mean of the distance in chain space to the nearest contact in the experimental structure (Figure S9); and (iv) quantification of the distribution (spread) of the contacts along the chain and over the structure of the protein, by measuring the mean of the distance from every experimental (crystal structure) contact to the nearest predicted contact (Figure S10).

Accuracy of prediction of *3D structure* is quantified in 3 ways: (i) using the TM score [53]; (ii) using GDT-TS [54]; and (iii) using the Pymol [81] ‘align’ routine, which reports the C_α_-RMSD for a moderately trimmed set of residues after iteratively removing the worst residue pairs from consideration as it finds an optimal superimposition of the residues in the predicted and the reference structure.

### (5) Comparison to other contact prediction methods

We calculated the four measures of contact prediction accuracy as in (4) above, for MI, BNM [13], [46] and SCA [66] [82]. We tested all three methods for their ability to generate protein folds for a number of families, using exactly the same pipeline as for the DI constraints of this work. Folding with constraints derived from MI or SCA did not achieve reasonable accuracy with any of the tested families (data not shown). However, constraints derived from BNM were successful in generating de novo predicted structures at less than 5 Å C_α_-RMSD for 6 of the 10 tested proteins.

Additional method details are in Text S1.

## Supporting Information

Figure S1 **Mutual Information (MI) contact maps.** (4 pages). Predicted contacts (blue dots) from high-ranking MI scores (excluding clashes with secondary structure prediction (see Text S1) and residues pairs 5 or less apart in the polypeptide chain). MI predicted contacts are overlaid onto contacts made in the corresponding crystal structure (grey circles), names as in Table 1. Contacts defined as 5 Å or less from any atom between the paired residues. Number of top-ranked MI contacts shown sorted into 4 groups: page 1, 150 (larger proteins); pages 2 and 3, 100 (medium size proteins); page 4 (smaller proteins), 50. MI ranked scores of residue couplings are available in Web Appendix A8.
(PDF)

Figure S2 **Evolutionary Inferred Contacts (EICs) (from Direct Information (DI)) contact maps.** (4 pages). Predicted contacts (red stars) from high-ranking DI scores (excluding clashes with secondary structure prediction (see Text S1), and residues pairs 5 or less apart in the polypeptide chain). EIC predicted contacts overlaid onto contacts made in the corresponding crystal structure (grey circles), names as in Table 1. Contacts defined as 5 Å or less from any atom between the paired residues. Number of top-ranked EIC contacts shown sorted into 4 groups: page 1, 150 (larger proteins); pages 2 and 3, 100 (medium size proteins); page 4 (smaller proteins), 50. EIC ranked scores of residue couplings are available in Web Appendix A1.
(PDF)

Figure S3 **Ribbon representations of top ranked predicted structures.** (4 pages). All 15 proteins showing on left, two views of top ranked predicted structure (turned 180°), and on the right, the same two views of representative crystal structure. Cartoon representation calculated in Pymol using predicted secondary structure (in predicted structures) and shown with rainbow coloring, blue N terminal, red C-terminal. All structure coordinates and Pymol sessions for top-ranking structures available in Web Appendices A3 and A4. Predicted structure IDs in order are: OPSD_BOVIN: PF00001_P02699_180_20, TRY2_RAT:PF00089_P00763_160_20, RASH_HUMAN:PF00071_P01112_130_17 RNH_ECOLI:PF00075_P0A7Y4_70_16, CHEY_ECOLI:PF00072_P0AE67_110_1, SPTB2_HUMAN:PF00307_Q01082_60_20 A8MVQ9_HUMAN:PF00059_Q9NNX6_110_20, THIO_ALIAC:PF00085_P80579_80_8, CADH1_HUMAN:PF00028_P12830_70_4, O45418_CAEEL:PF00254_O45418_50_9, OMPR_ECOLI:PF00486_P0AA16_40_18, ELAV4_HUMAN:PF00076_P26378_40_12, PCBP1_HUMAN:PF00013_Q15365_40_15, BPT1_BOVIN:PF00014_P00974_30_5, YES_HUMAN:PF00018_P07947_40_2.
(PDF)

Figure S4 **Active sites of top-ranked predicted Trypsin and Ras structures.** **A.** Overlay of 3 catalytic residues from top-ranked predicted trypsin structure and 3tgi **B.** Overlay of 4 residues involved in the GTP binding site from the top-ranked predicted Ras structure and 5p21. Pymol session available in Web Appendix A4.
(PDF)

Figure S5 **Discrimination scores of predicted structures.** (2 pages) Scores are calculated for every predicted structure using quality of virtual torsion between predicted β strands and within α helices (Web Appendix A5, Text S1). Here, scoring of candidate structures is assessed by comparing the ranking score of the predicted structures with the experimentally observed structure of the chosen reference protein (PDB), see Table 1 for PDB names.
(PDF)

Figure S6 **True positive rate of predicted contacts for 4 methods.** For each of the 15 proteins, plots show the proportion of true positives over a range of top ranking constraint numbers (10–200) for 4 different contact prediction methods. EIC (DI), this work, shown in red, BNM [13] in green, SCA [61] in black and MI ( our calculation) in blue. True positive is defined as within 5 Å minimum atom distance. Contact predictions from all methods were treated equivalently, with predicted secondary structure clashes, more than one cysteine pairing per cysteine, and >90% conserved residues removed, see Text S1 for pipeline. Although the DI/EIC contacts almost always have the best true positive proportion, the BNM method is favorable in some cases.
(PDF)

Figure S7 **The minimum atom distance of top 200 ranked DI pairs.** (4 pages). For each for the 15 proteins, plots show the minimum distance between each DI ranked residue pair. In red are the EICs and in purple the Dis which are filtered by our algorithm. Note that for many proteins, especially A8MVQ9_HUMAN (lectin C ) and Trypsin, high ranking DIs which are false positives are removed from the EICs used for folding, whereas others, for example Ras and Chey are hardly affected, Text S1 and all scores available in Web Appendix A1. Note that the scale changes for each protein.
(PDF)

Figure S8 **Relationship between proportion of false positives and 3D structure prediction accuracy.** (4 pages). For all 15 proteins, comparison of the proportion of false positives in 20 sets of constraint numbers ranging from 10–200, compared to the best C_α_-RMSD accuracy for a structure predicted using the same number of EIC constraints. Some proteins such as the SH3 domain of YES and trypsin inhibitor, how a clear decline in best predicted structure accuracy with increasing proportion of false positive contact and others such as CheY show the inverse relationship. However, those that show an inverse relationship tends to have a lower rate of FPs overall and all proteins show best accuracy at FP proportion below 0.4.
(PDF)

Figure S9 **Quantitative false positive assessment.** (4 pages). Since a false positive calculation is typically is limited to a binary count (it is counted as either a false positive or not), we developed a metric to compare how far ‘wrong’ the FPs are. For each predicted EIC constraint, in N-scoring residue pairs (10–200) we calculate the Euclidean 2D distance to the nearest contact in the crystal structure and report the mean of this distance for each Nc, over all 15 proteins. This is repeated for each of the other contact predicted methods, MI, BNM and SCA. Red, DI: blue, MI; green, BNM: black SCA.
(PDF)

Figure S10 **Quantitative assessment of spread of predicted contacts (4 pages).** True positive counts alone do not reflect how well-distributed the top N-scoring pairs are across the protein. Therefore we developed a metric to measure how well the top N-scoring residue pairs ‘cover’ the contact map of a corresponding crystal structure. We compute the Euclidean 2D distance between the contact map of the corresponding crystal structure, and the contact map consisting of the top N-scoring residue pairs. For each residue pair, separated by more than five residues in sequence, we compute the distance to the nearest high-scoring residue pair (for instance, the nearest ‘red star’ in the contact map, in the case of EIC pairs). For each set of N-scoring residue pairs we calculate the mean of the distances for all contacts to the nearest contact in the crystal structure. Plotted is the mean spread for each Nc for 4 methods, across all 15 proteins. Red, DI: blue, MI; green, BNM: black SCA.
(PDF)

Figure S11 **Bayesian Network Model (BNM) contact maps.** (4 pages). Predicted contacts (blue dots) from high-ranking BNM scores excluding clashes with secondary structure prediction (see Text S1) and residues pairs 5 or less apart in the polypeptide chain. BNM predicted contacts overlaid onto contacts made in the corresponding crystal structure (grey circles), names as in Table 1. Contacts defined as 5 Å or less from any atom between the paired residues. Number of top-ranked BNM contacts shown sorted into 4 groups: page 1, 150 (larger proteins); pages 2 and 3, 100 (medium size proteins); page 4 (smaller proteins), 50. BNM ranked scores of residue couplings are available in Web Appendix A8.
(PDF)

Figure S12 **Statistical Coupling Analysis (SCA) contact maps.** (4 pages). Predicted contacts (blue dots) from high-ranking SCA scores excluding clashes with secondary structure prediction (see Text S1) and residues pairs 5 or less apart in the polypeptide chain. SCA predicted contacts overlaid onto contacts made in the corresponding crystal structure (grey circles), names as in Table 1. Contacts defined as 5 Å or less from any atom between the paired residues. Number of top-ranked SCA contacts shown sorted into 4 groups: page 1, 150 (larger proteins); pages 2 and 3, 100 (medium size proteins); page 4 (smaller proteins), 50. SCA ranked scores of residue couplings are available in Web Appendix A8.
(PDF)

Figure S13 **The minimum atom distance of top 200 ranked MI pairs.** (4 pages). For each for the 15 proteins, plots show the minimum distance between each MI ranked residue pair. In purple the MIs which are filtered out by our algorithm, Text S1 and all scores are available in Web Appendix A8.
(PDF)

Figure S14 **The minimum atom distance of top 200 ranked BNM pairs.** (4 pages). For each for the 15 proteins, plots show the minimum distance between each DI ranked residue pair. In purple the BNMs that are filtered by our algorithm, Text S1 and all scores available in Web Appendix A9.
(PDF)

Figure S15 **The minimum atom distance of top 200 ranked SCA pairs.** (4 pages). For each for the 15 proteins, plots show the minimum distance between each DI ranked residue pair. In purple the SCAs which are filtered by our algorithm, Text S1 and all scores available in Web Appendix A10.
(PDF)

Figure S16 **Number of distance constraints required for correct 3D structure prediction.** With increasing number of predicted essential distance constraints (*N_C_*, horizontal axis), 3D prediction error decreases rapidly, as assessed by C_α_-RMSD between the best of 20 (in each *N_C_* bin) predicted structures and the observed structure (here, for the 15 test proteins, using Pymol) shown separately. Remarkably, as few as ∼*N_RES_/2* (∼*L/2*) distance constraints *d_ij_* (with chain distance *|i−j|*>5) suffice for good quality predictions below 5 Å C_α_-RMSD, where *N_RES_* is the number of amino acid residues in the protein multiple sequence alignment.
(PDF)

Text S1 **Supplementary Methods and Analysis.**
(PDF)

Table S1 **Protein 3D structure computed from evolutionary sequence variation.**
(XLS)

Table S2 **Conflict resolution between DIs and predicted secondary structure constraints.**
(DOC)

Table S3 **Distance ranges used for predicted secondary structural elements in folding calculations.**
(DOC)

Table S4 **β sheet detection in predicted structures.**
(DOC)

Table S5 **Control calculations testing real distances.**
(DOC)
