# Supplementary material for: Protein 3D Structure Computed from Evolutionary Sequence Variation
Source: PLoS One. 2011 Dec 7;6(12):e28766. doi: 10.1371/journal.pone.0028766 (PMC3233603; doi:10.1371/journal.pone.0028766)

Figure S1. Mutual Information (MI) contact maps.

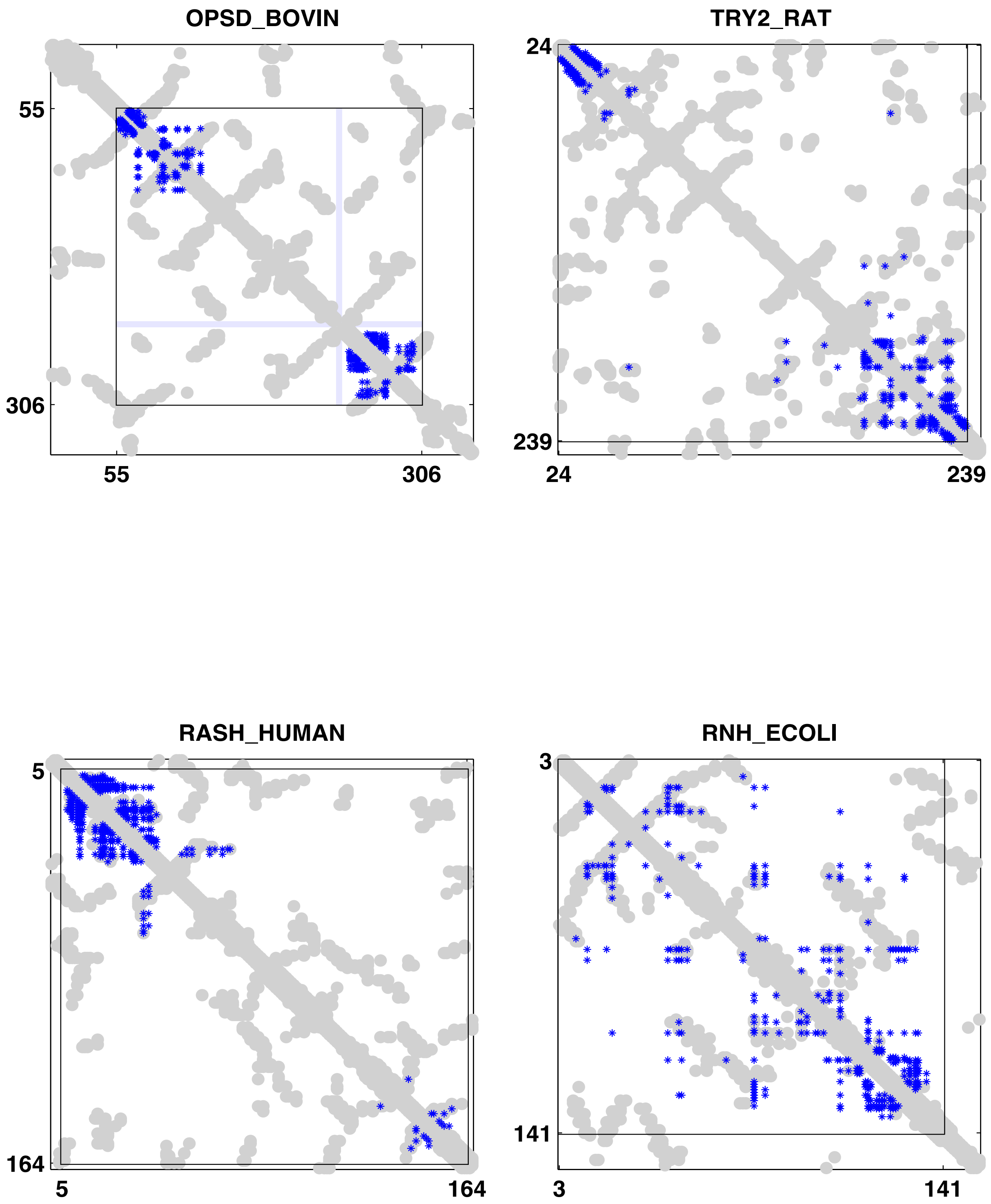

Figure S1. Mutual Information (MI) contact maps.

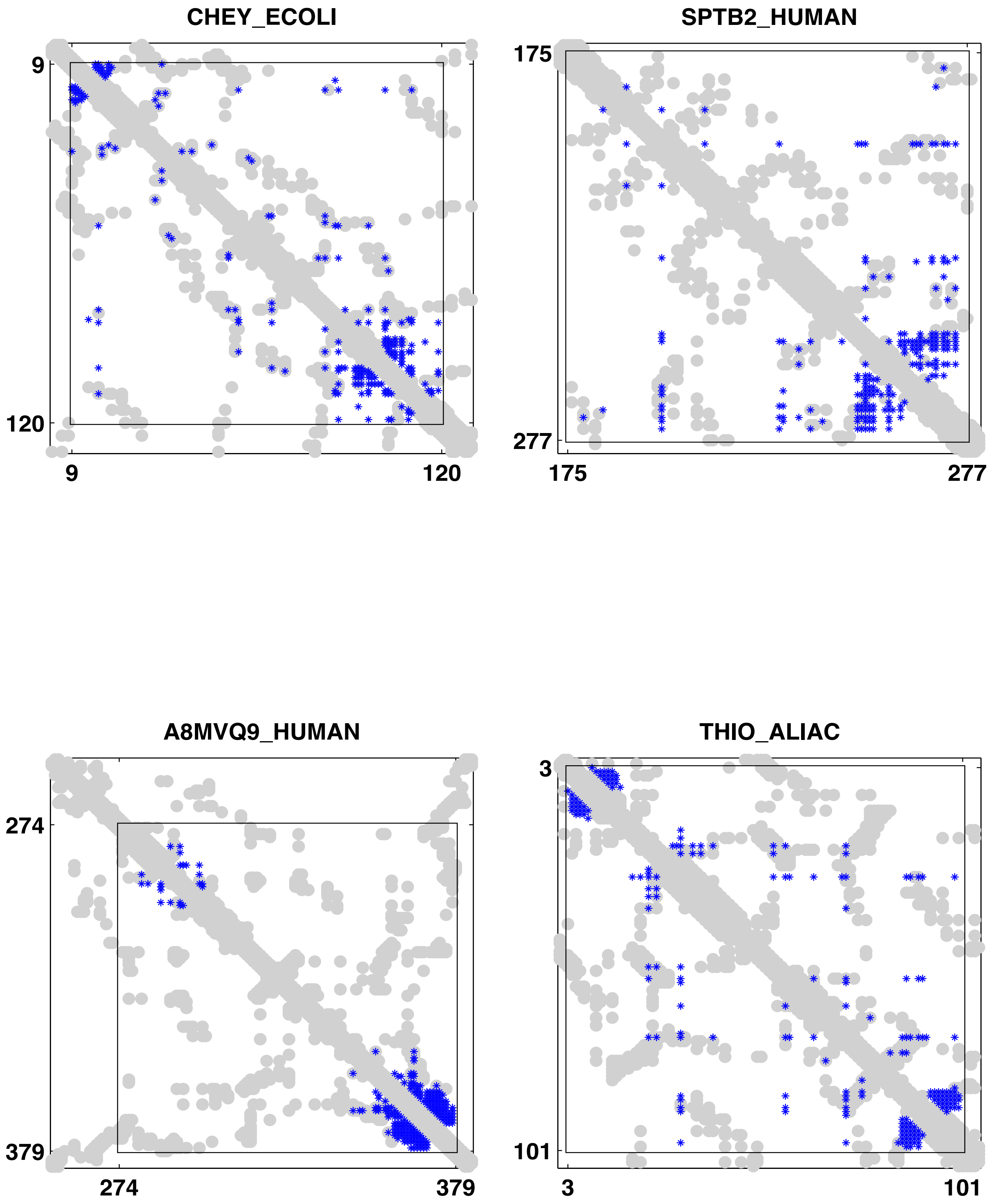

Figure S1. Mutual Information (MI) contact maps.

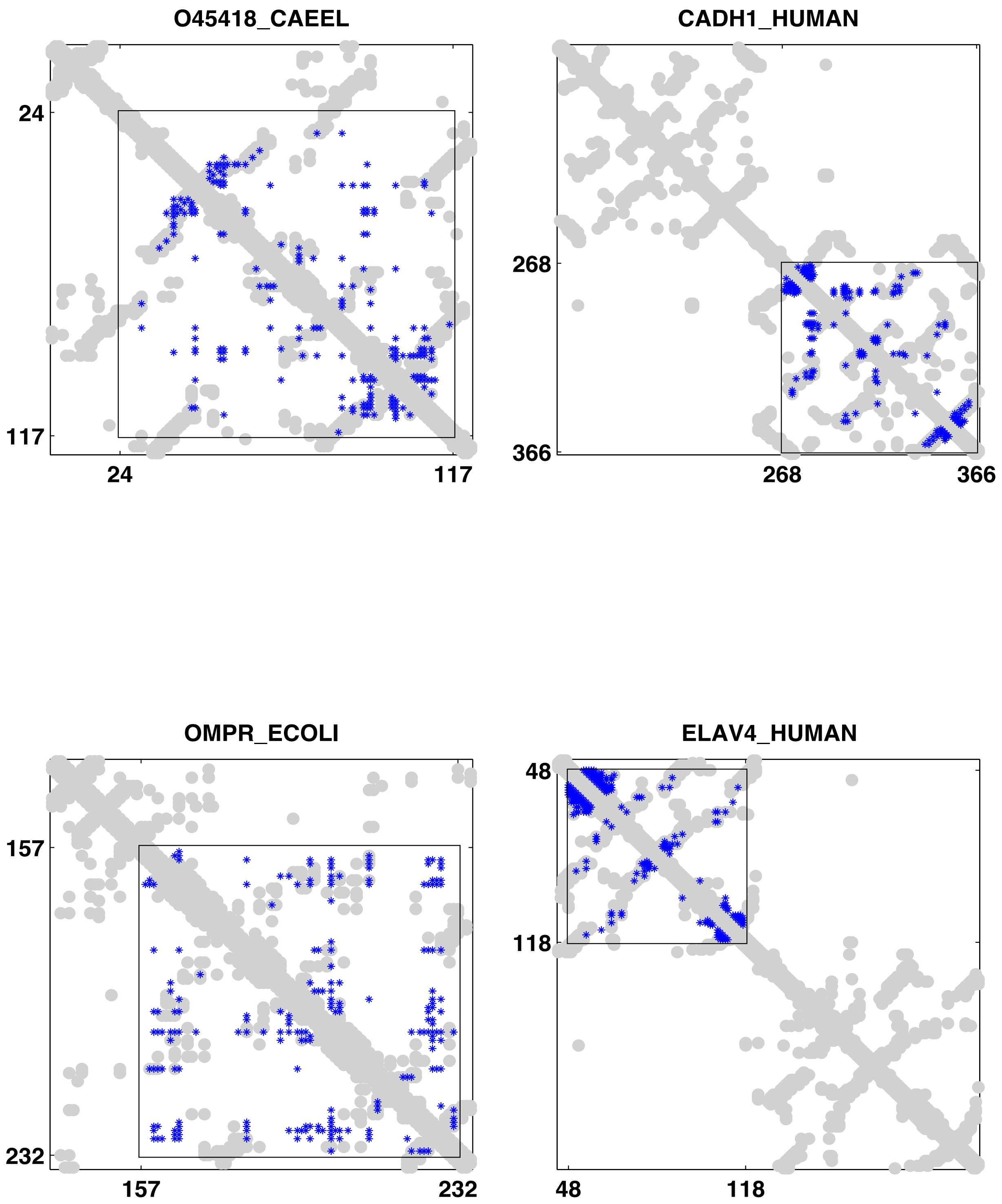

Figure S1. Mutual Information (MI) contact maps.

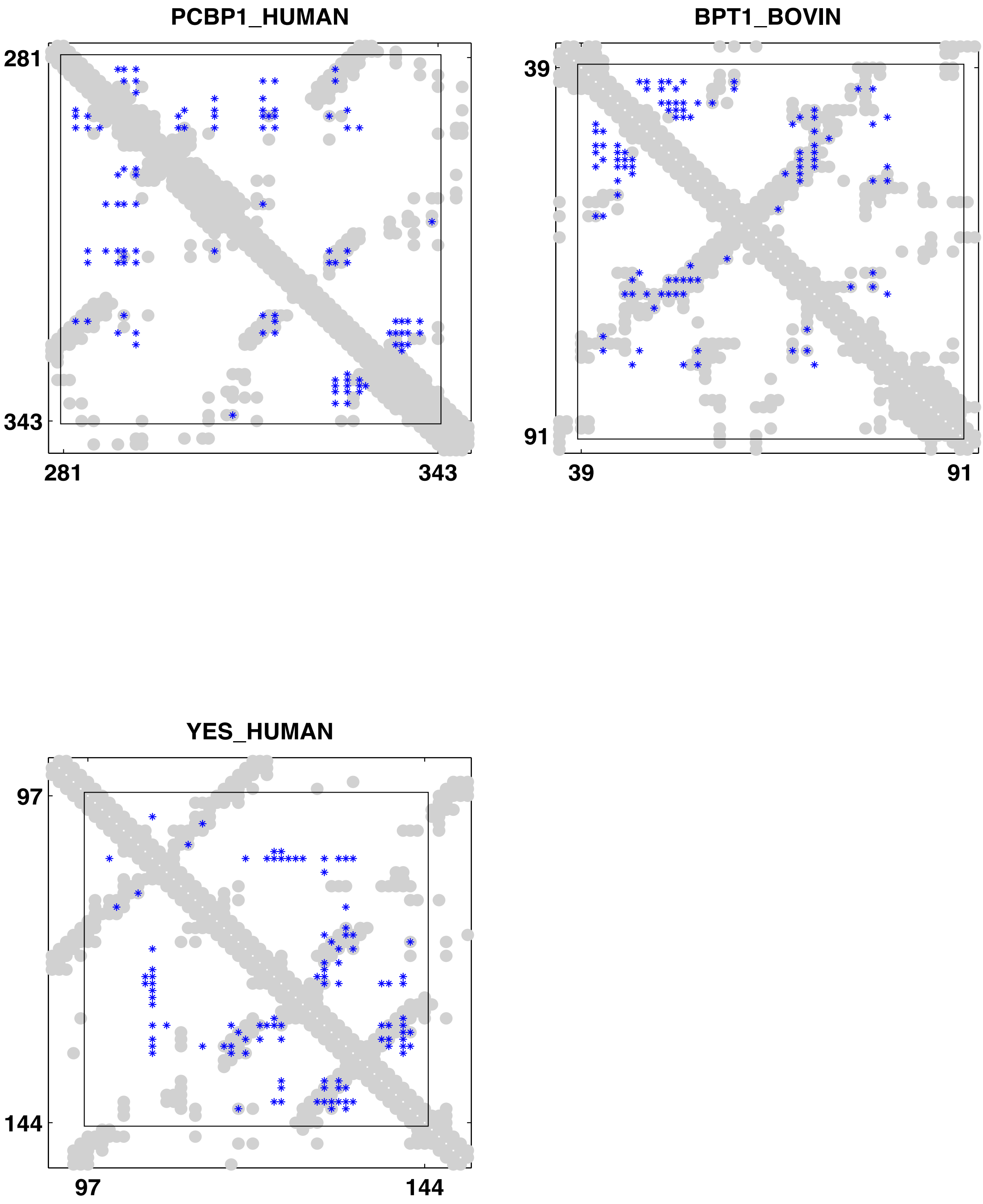

Supplement: Figure S1 — Mutual Information (MI) contact maps. (4 pages). Predicted contacts (blue dots) from high-ranking MI scores (excluding clashes with secondary structure prediction (see Text S1) and residues pairs 5 or less apart in the polypeptide chain). MI predicted contacts are overlaid onto contacts made in the corresponding crystal structure (grey circles), names as in Table 1. Contacts defined as 5 Å or less from any atom between the paired residues. Number of top-ranked MI contacts shown sorted into 4 groups: page 1, 150 (larger proteins); pages 2 and 3, 100 (medium size proteins); page 4 (smaller proteins), 50. MI ranked scores of residue couplings are available in Web Appendix A8. (PDF) [file pone.0028766.s001.pdf]
