# Supplementary material for: Protein 3D Structure Computed from Evolutionary Sequence Variation
Source: PLoS One. 2011 Dec 7;6(12):e28766. doi: 10.1371/journal.pone.0028766 (PMC3233603; doi:10.1371/journal.pone.0028766)

Figure S2. Evolutionary Inferred Contacts (EICs) (from Direct Information (DI)) contact maps.

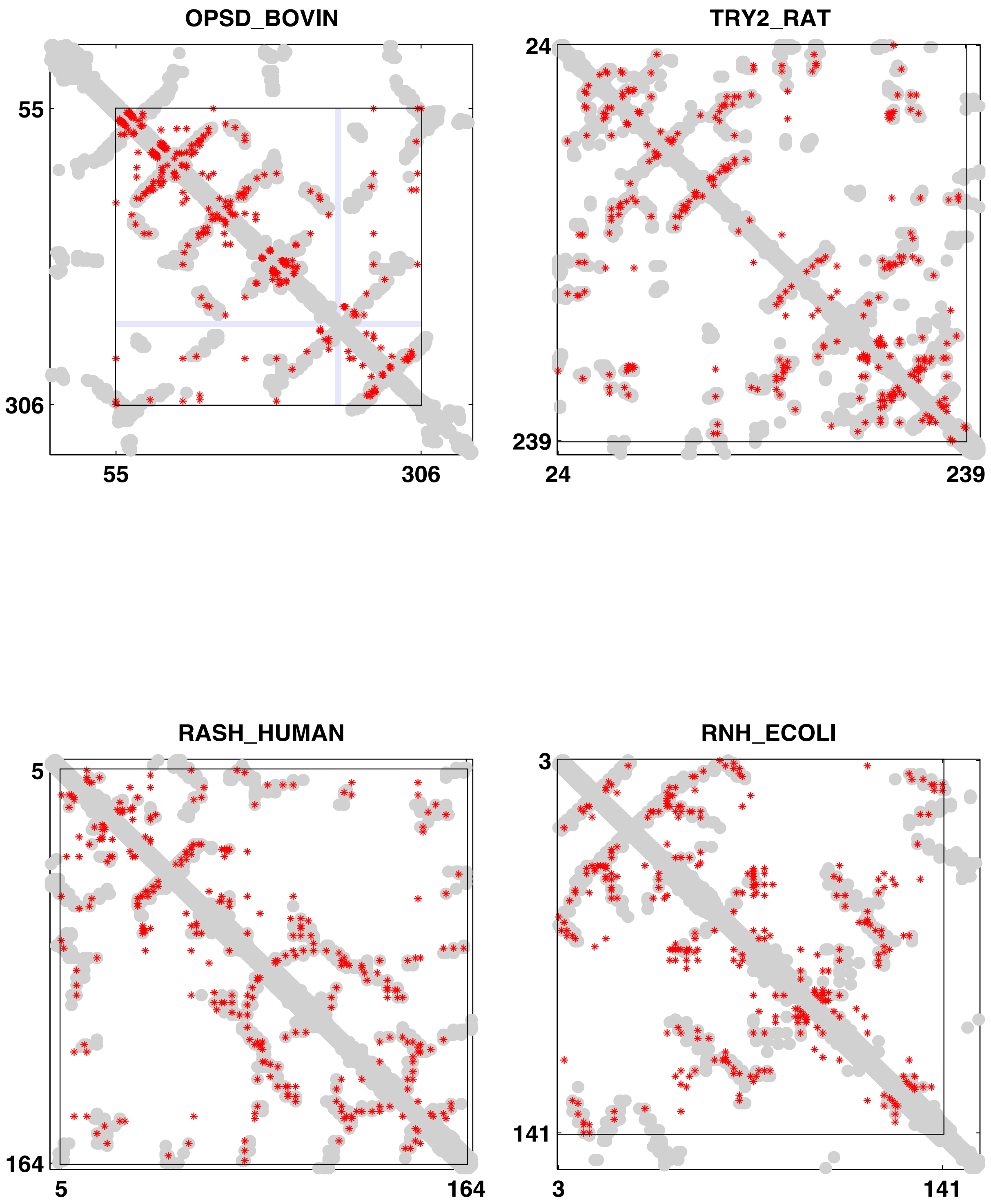

Figure S2. Evolutionary Inferred Contacts (EICs) (from Direct Information (DI)) contact maps.

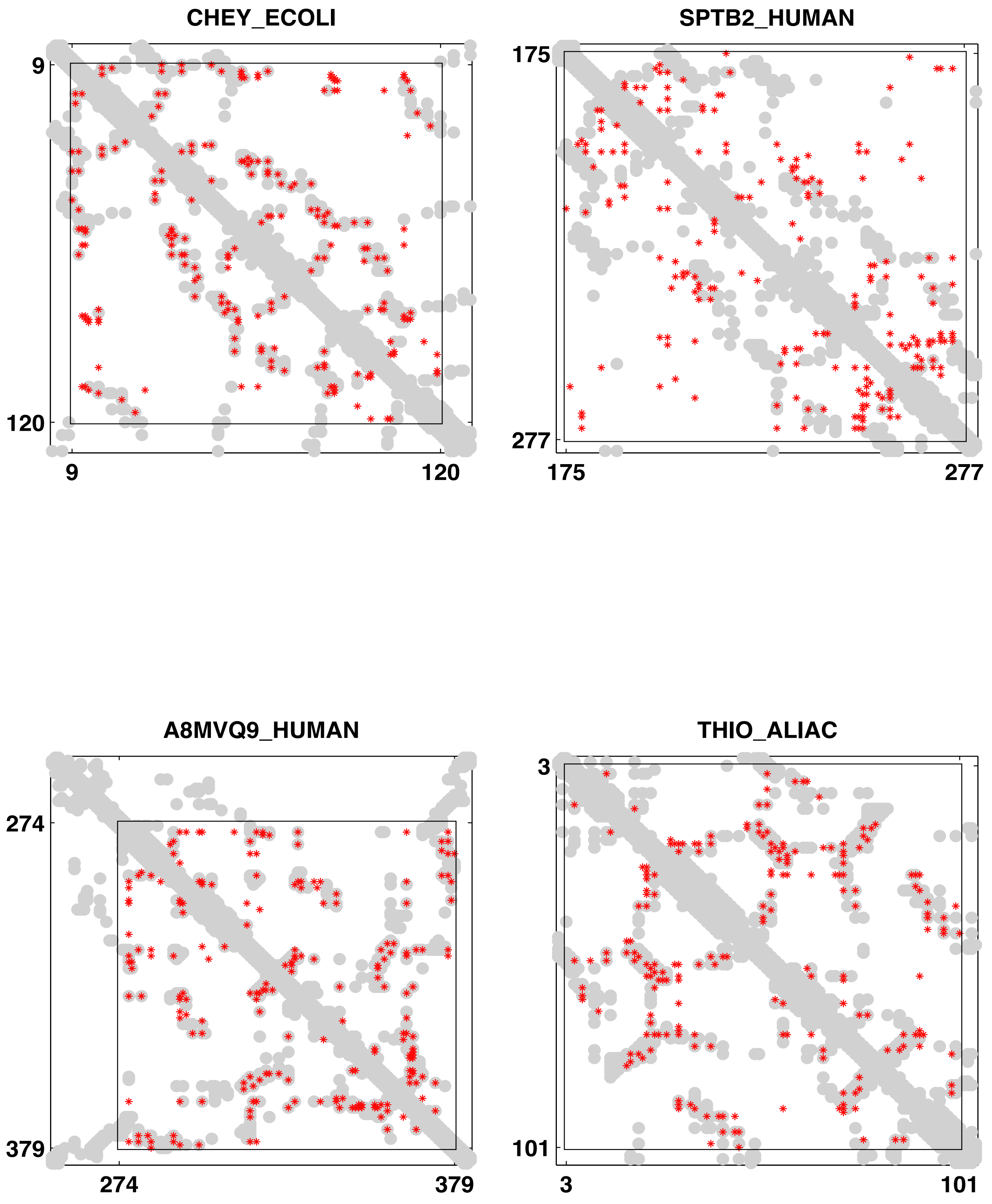

Figure S2. Evolutionary Inferred Contacts (EICs) (from Direct Information (DI)) contact maps.

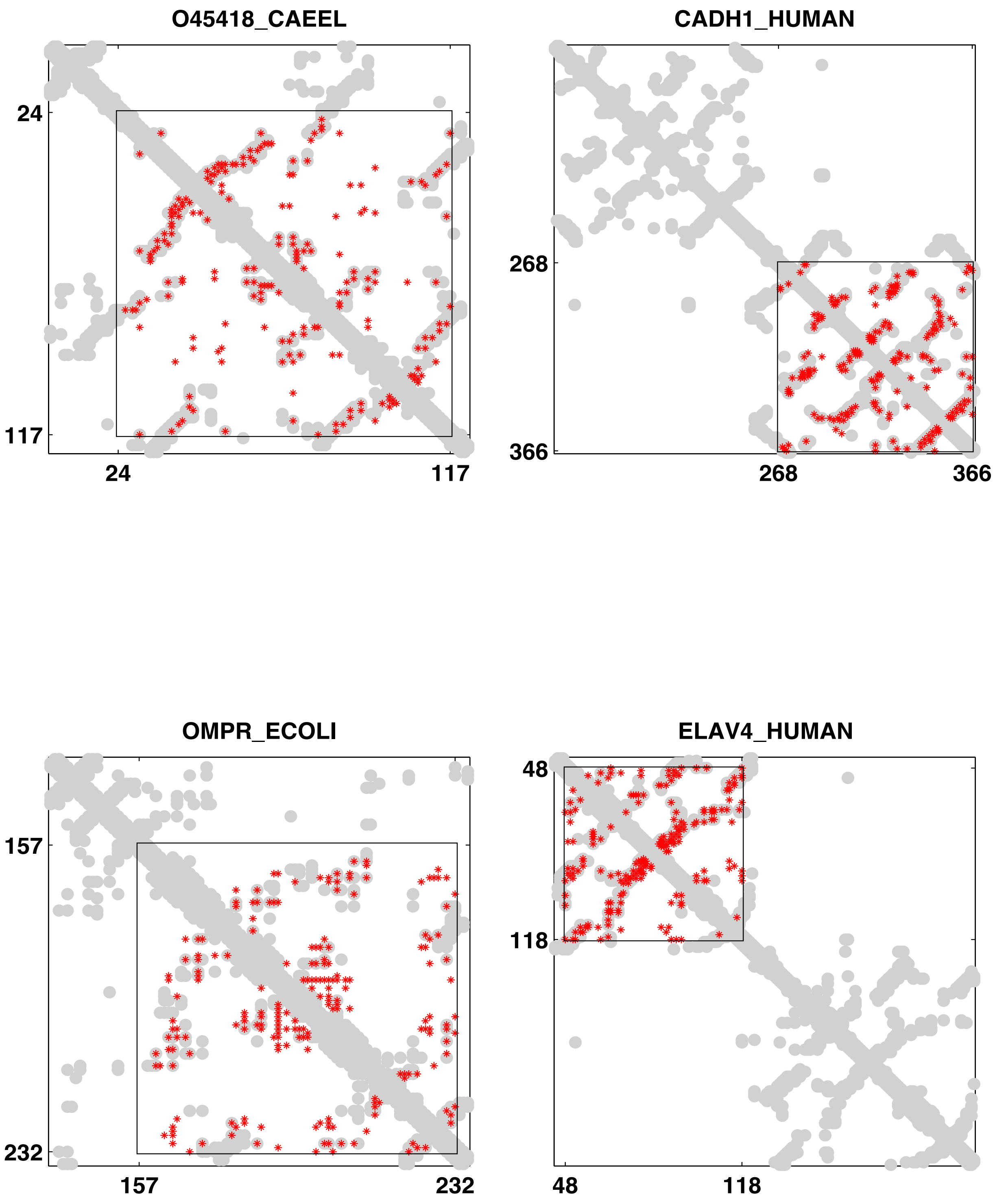

Figure S2. Evolutionary Inferred Contacts (EICs) (from Direct Information (DI)) contact maps.

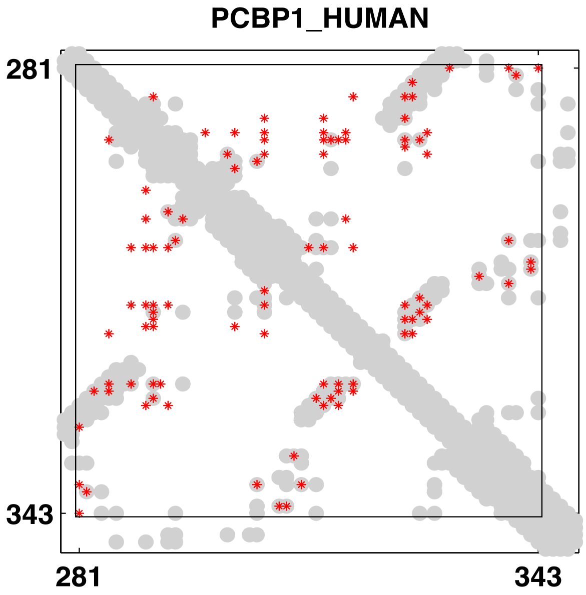

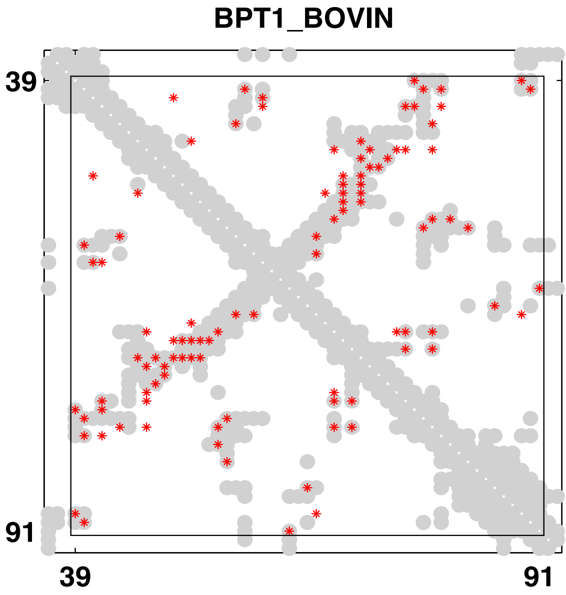

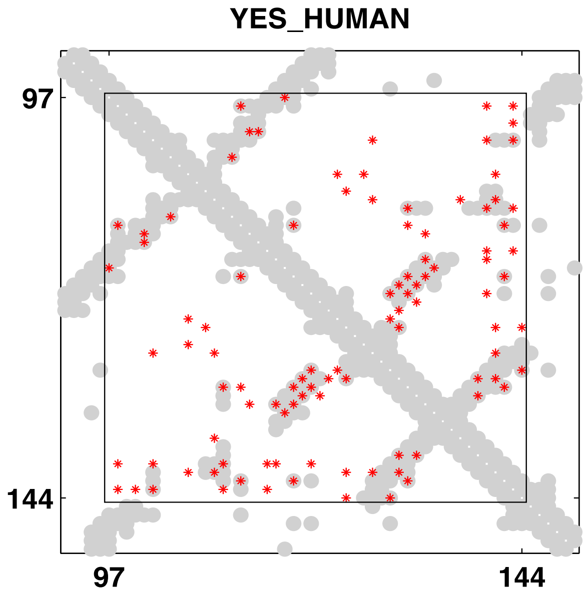

Supplement: Figure S2 — Evolutionary Inferred Contacts (EICs) (from Direct Information (DI)) contact maps. (4 pages). Predicted contacts (red stars) from high-ranking DI scores (excluding clashes with secondary structure prediction (see Text S1), and residues pairs 5 or less apart in the polypeptide chain). EIC predicted contacts overlaid onto contacts made in the corresponding crystal structure (grey circles), names as in Table 1. Contacts defined as 5 Å or less from any atom between the paired residues. Number of top-ranked EIC contacts shown sorted into 4 groups: page 1, 150 (larger proteins); pages 2 and 3, 100 (medium size proteins); page 4 (smaller proteins), 50. EIC ranked scores of residue couplings are available in Web Appendix A1. (PDF) [file pone.0028766.s002.pdf]
