# Supplementary material for: Protein 3D Structure Computed from Evolutionary Sequence Variation
Source: PLoS One. 2011 Dec 7;6(12):e28766. doi: 10.1371/journal.pone.0028766 (PMC3233603; doi:10.1371/journal.pone.0028766)

Figure S3. Ribbon representations of top ranked predicted structures.

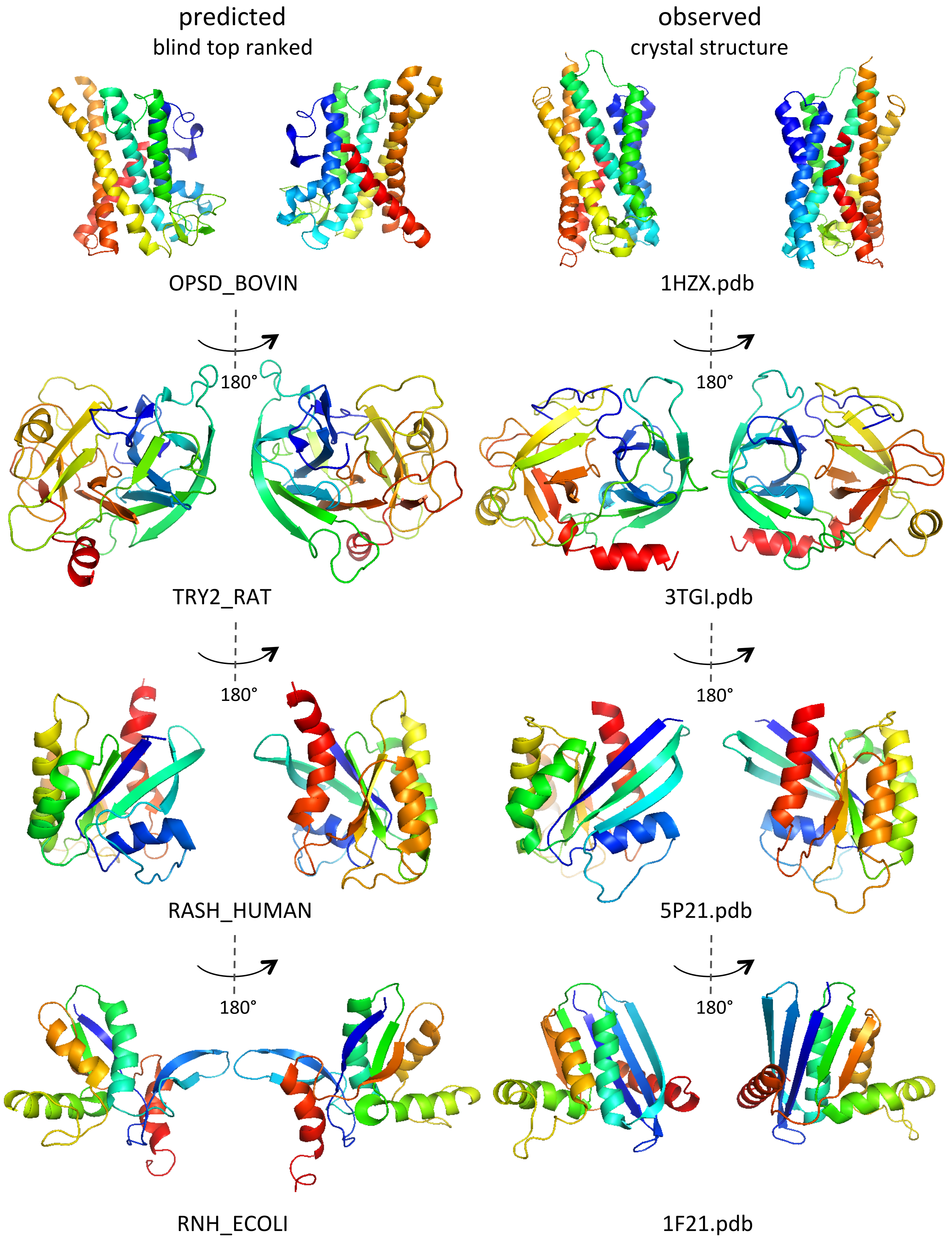

Figure S3. Ribbon representations of top ranked predicted structures.

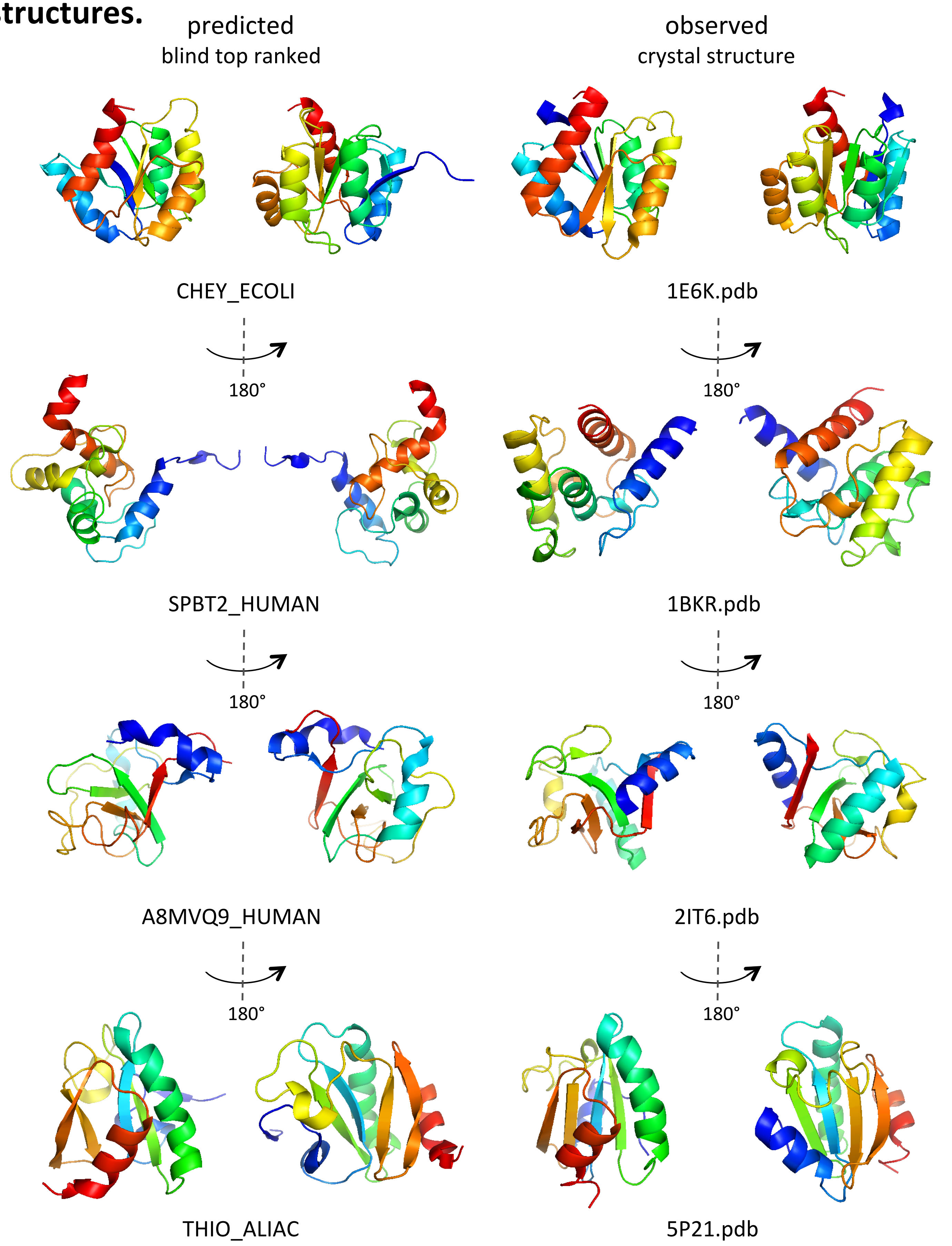

Figure S3. Ribbon representations of top ranked predicted structures.

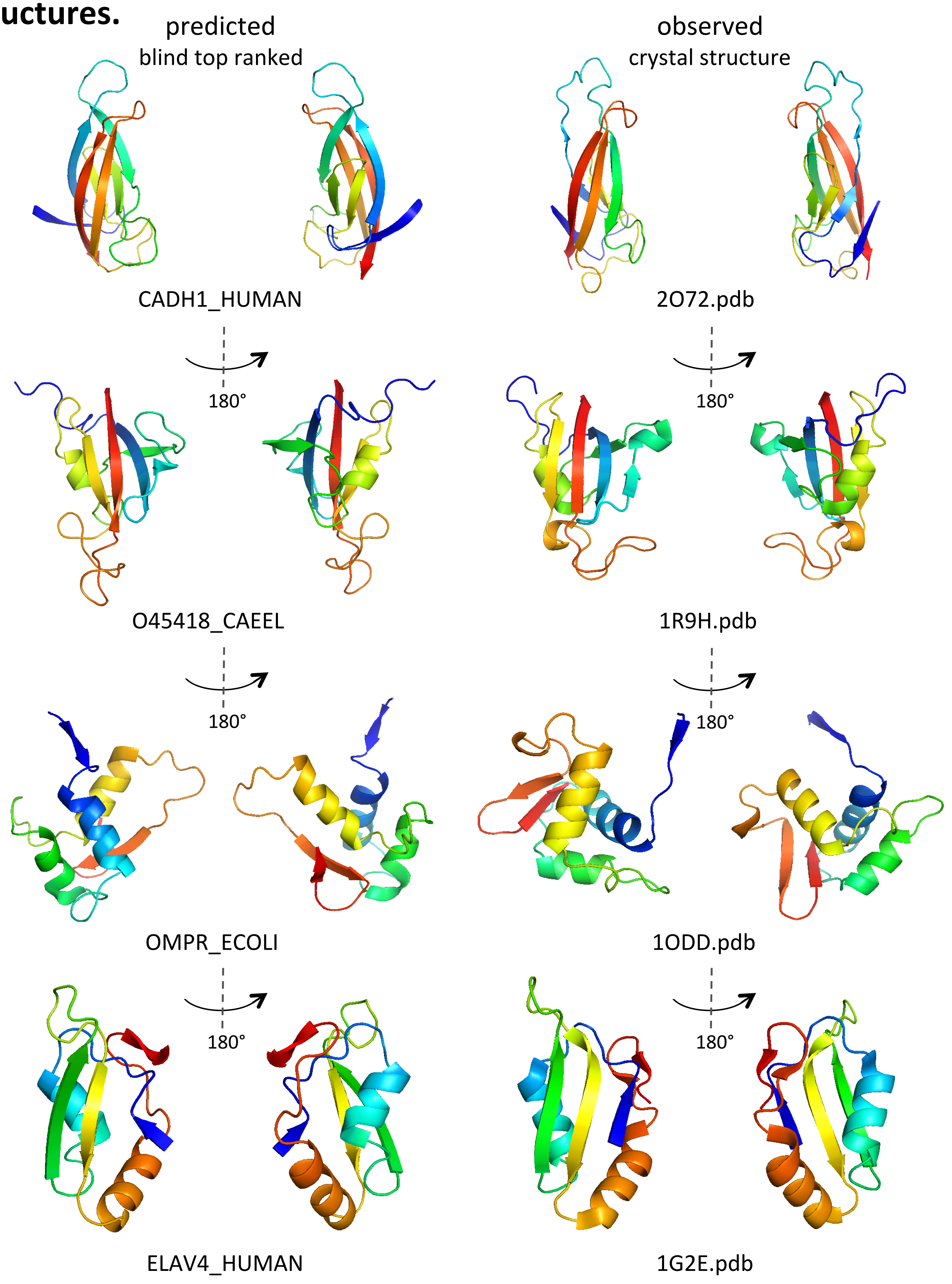

Figure S3. Ribbon representations of top ranked predicted structures.

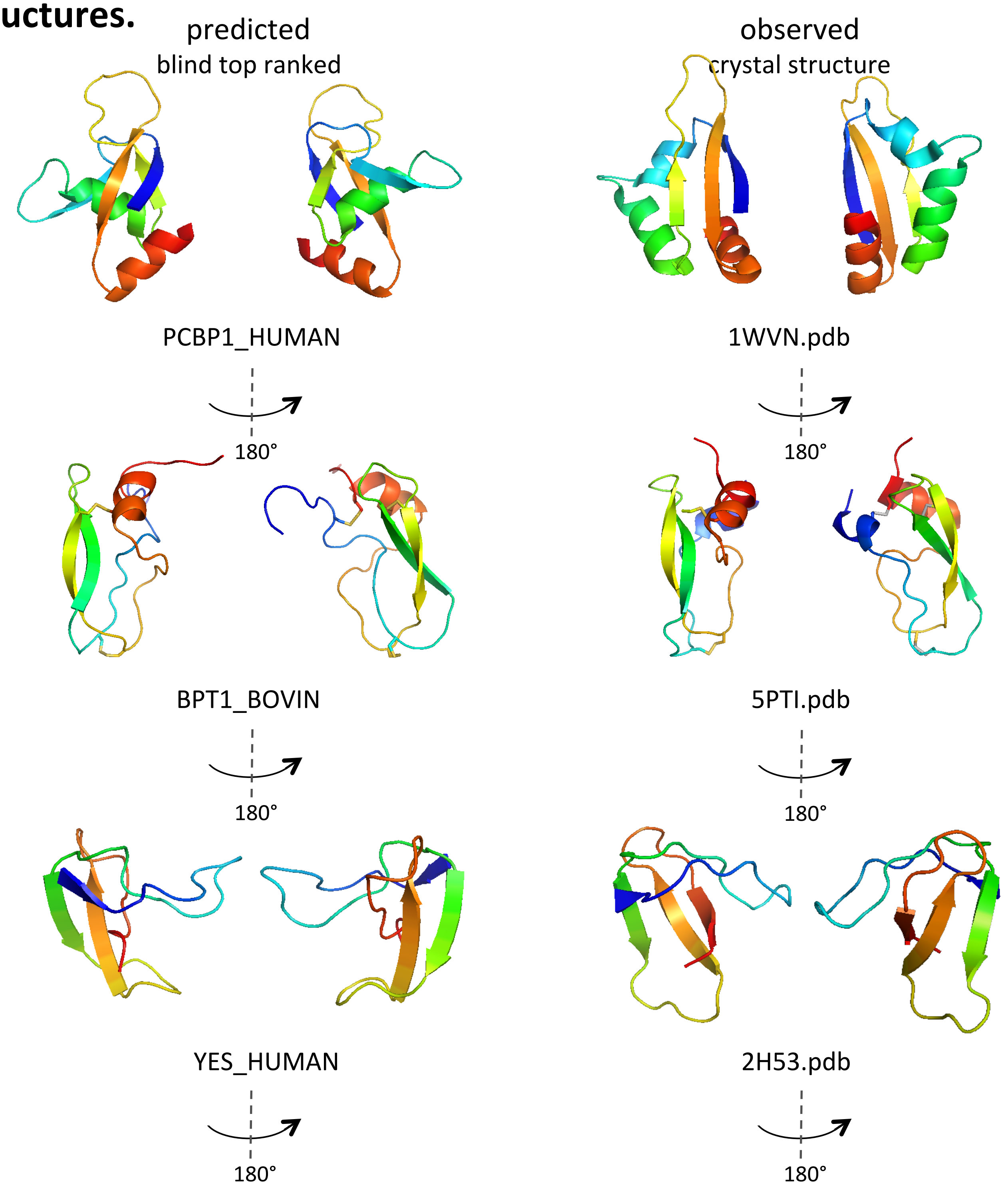

Supplement: Figure S3 — Ribbon representations of top ranked predicted structures. (4 pages). All 15 proteins showing on left, two views of top ranked predicted structure (turned 180°), and on the right, the same two views of representative crystal structure. Cartoon representation calculated in Pymol using predicted secondary structure (in predicted structures) and shown with rainbow coloring, blue N terminal, red C-terminal. All structure coordinates and Pymol sessions for top-ranking structures available in Web Appendices A3 and A4. Predicted structure IDs in order are: OPSD_BOVIN: PF00001_P02699_180_20, TRY2_RAT:PF00089_P00763_160_20, RASH_HUMAN:PF00071_P01112_130_17 RNH_ECOLI:PF00075_P0A7Y4_70_16, CHEY_ECOLI:PF00072_P0AE67_110_1, SPTB2_HUMAN:PF00307_Q01082_60_20 A8MVQ9_HUMAN:PF00059_Q9NNX6_110_20, THIO_ALIAC:PF00085_P80579_80_8, CADH1_HUMAN:PF00028_P12830_70_4, O45418_CAEEL:PF00254_O45418_50_9, OMPR_ECOLI:PF00486_P0AA16_40_18, ELAV4_HUMAN:PF00076_P26378_40_12, PCBP1_HUMAN:PF00013_Q15365_40_15, BPT1_BOVIN:PF00014_P00974_30_5, YES_HUMAN:PF00018_P07947_40_2. (PDF) [file pone.0028766.s003.pdf]
