# Supplementary material for: Protein 3D Structure Computed from Evolutionary Sequence Variation
Source: PLoS One. 2011 Dec 7;6(12):e28766. doi: 10.1371/journal.pone.0028766 (PMC3233603; doi:10.1371/journal.pone.0028766)
Supplement: Figure S4 — Active sites of top-ranked predicted Trypsin and Ras structures. A. Overlay of 3 catalytic residues from top-ranked predicted trypsin structure and 3tgi B. Overlay of 4 residues involved in the GTP binding site from the top-ranked predicted Ras structure and 5p21. Pymol session available in Web Appendix A4. (PDF) [file pone.0028766.s004.pdf]

Figure S4. Active sites of top-ranked predicted Trypsin and Ras structures

A. Trypsin – 3 catalytic residues

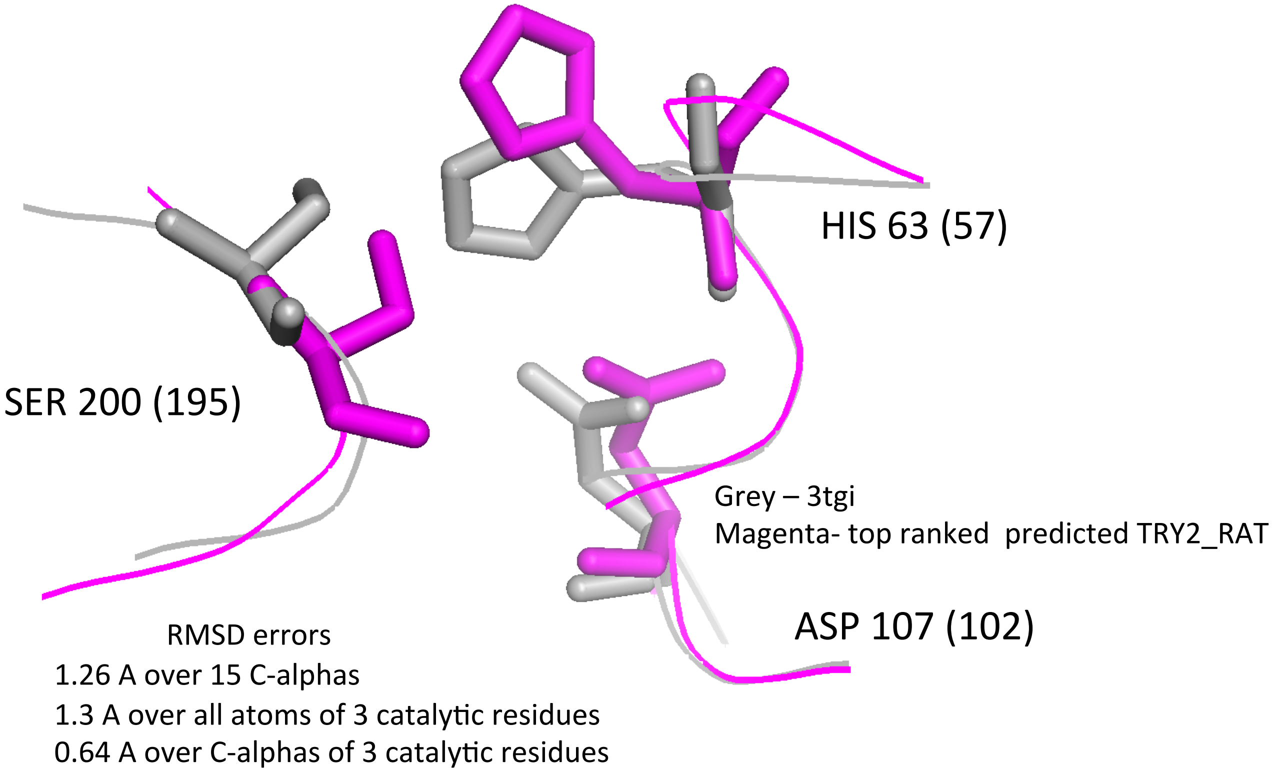

B. RAS GDP binding site

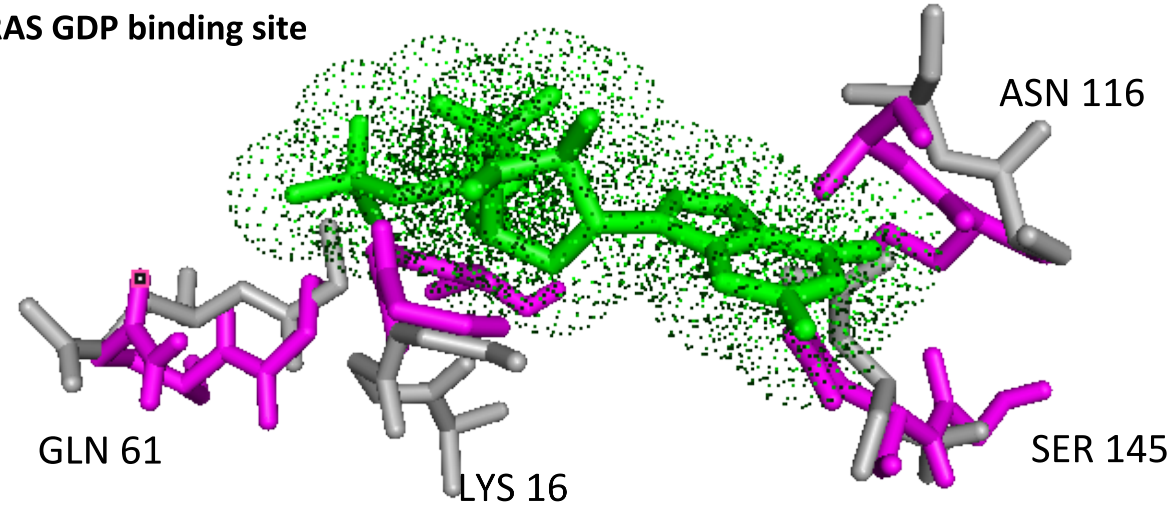

Grey: 5p21, green: bound GDP, magenta: top ranked predicted Ras structure
