# Supplementary material for: Protein 3D Structure Computed from Evolutionary Sequence Variation
Source: PLoS One. 2011 Dec 7;6(12):e28766. doi: 10.1371/journal.pone.0028766 (PMC3233603; doi:10.1371/journal.pone.0028766)

Figure S5. Discrimination accuracy of predicted structures

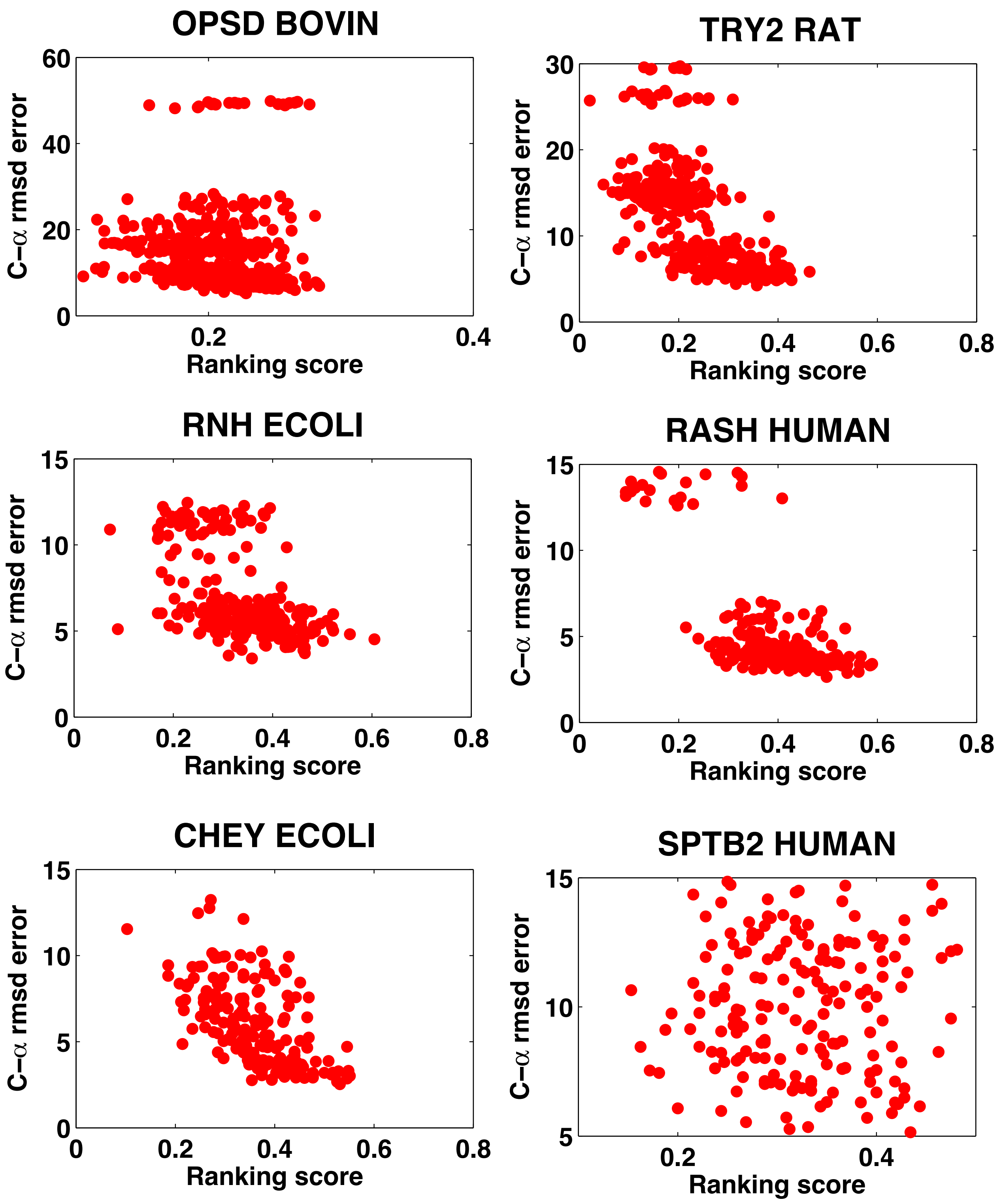

Figure S5. Discrimination accuracy of predicted structures

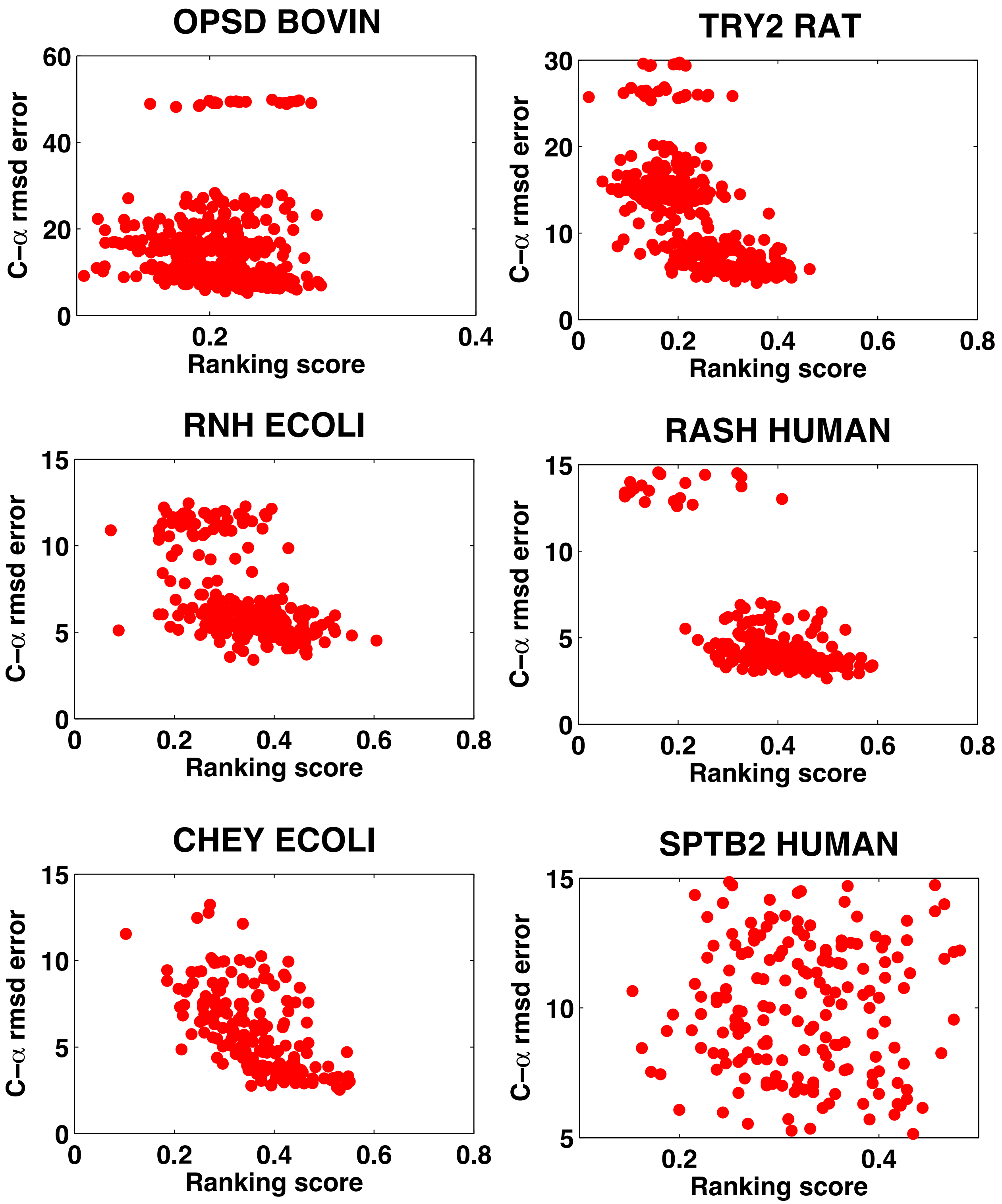

Figure S5. Discrimination accuracy of predicted structures.

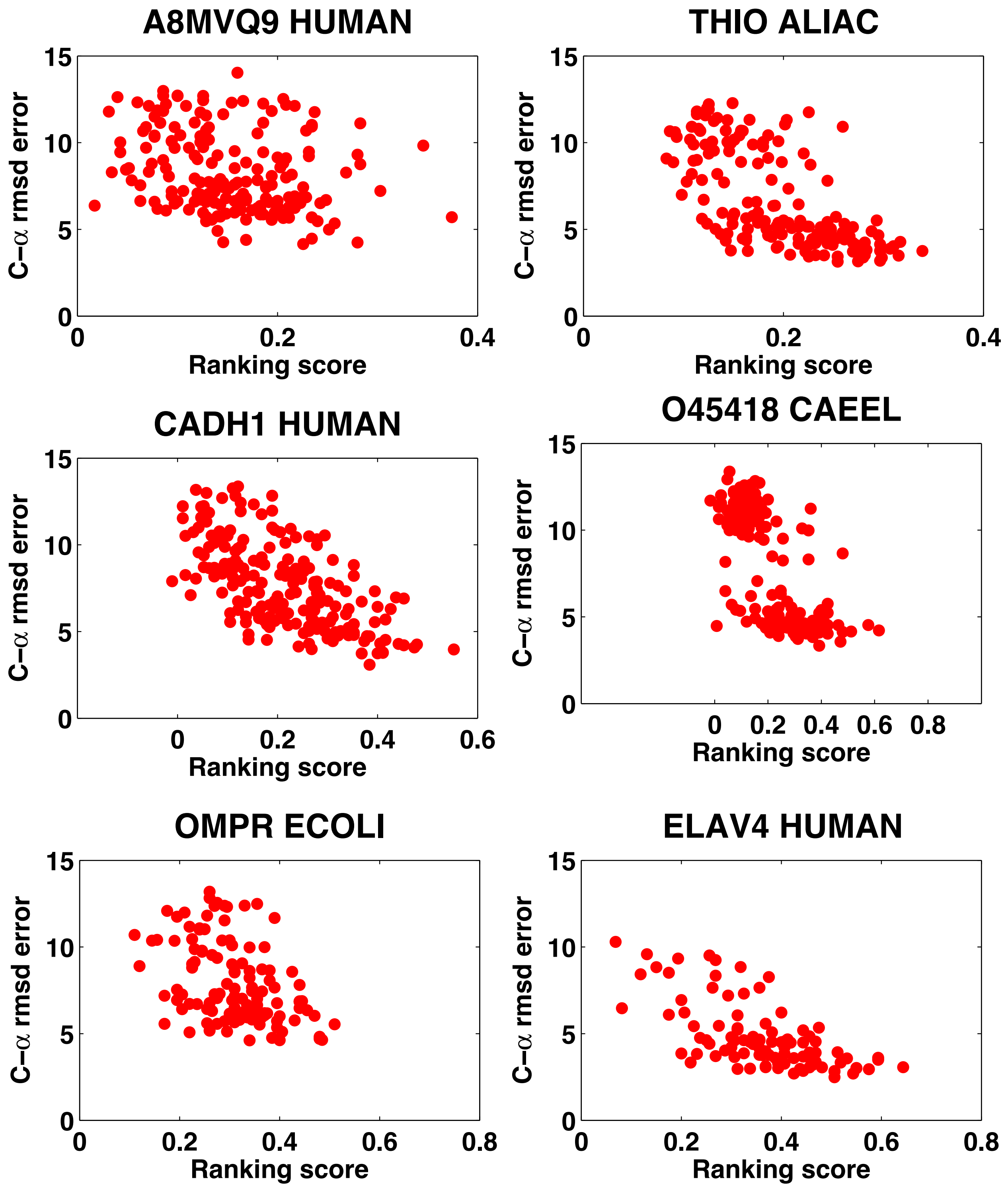

Figure S5. Discrimination accuracy of predicted structures

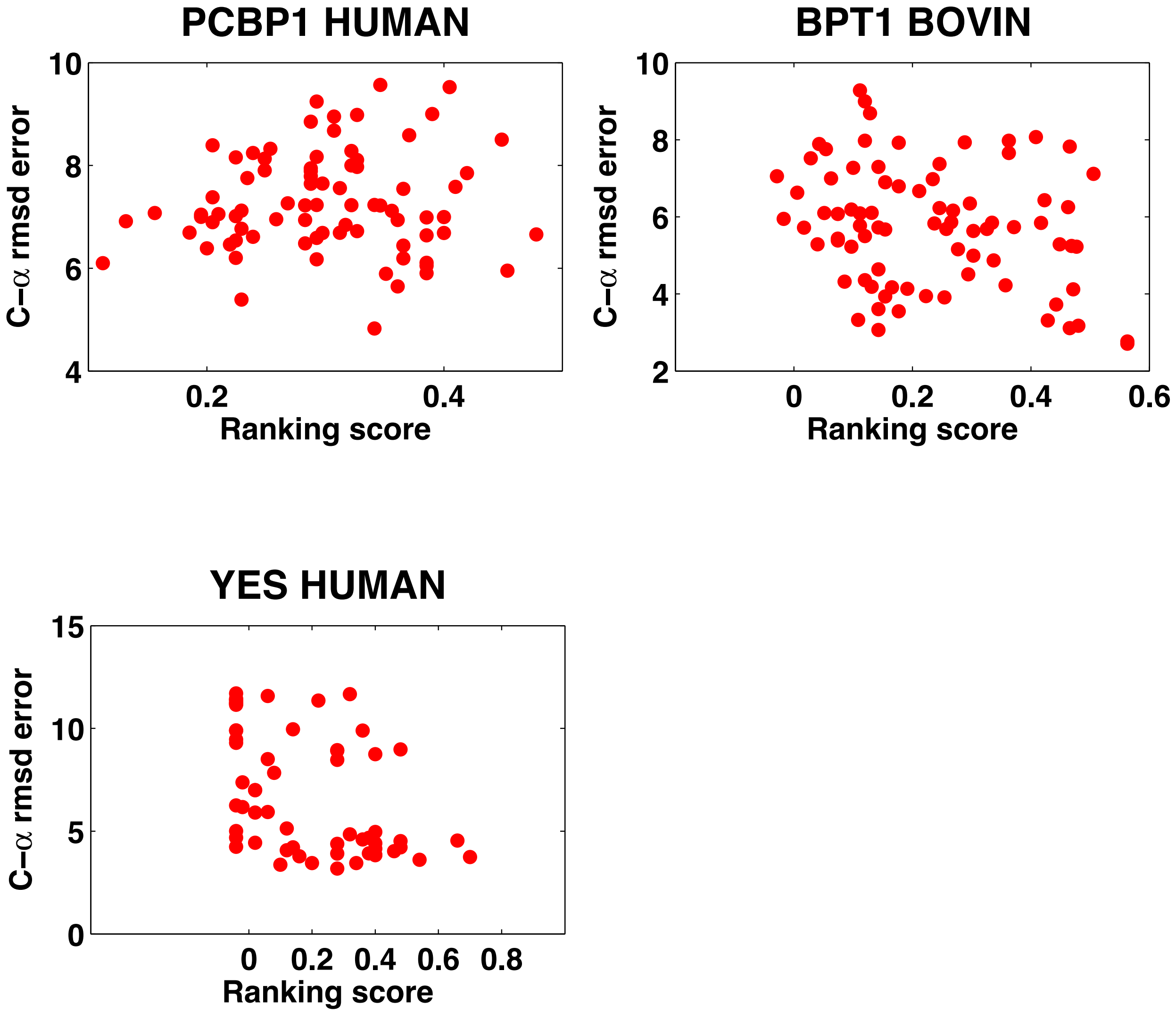

Supplement: Figure S5 — Discrimination scores of predicted structures. (2 pages) Scores are calculated for every predicted structure using quality of virtual torsion between predicted β strands and within α helices (Web Appendix A5, Text S1). Here, scoring of candidate structures is assessed by comparing the ranking score of the predicted structures with the experimentally observed structure of the chosen reference protein (PDB), see Table 1 for PDB names. (PDF) [file pone.0028766.s005.pdf]
