# Supplementary material for: Protein 3D Structure Computed from Evolutionary Sequence Variation
Source: PLoS One. 2011 Dec 7;6(12):e28766. doi: 10.1371/journal.pone.0028766 (PMC3233603; doi:10.1371/journal.pone.0028766)

Figure S6. True positive rate of predicted contacts for 4 methods

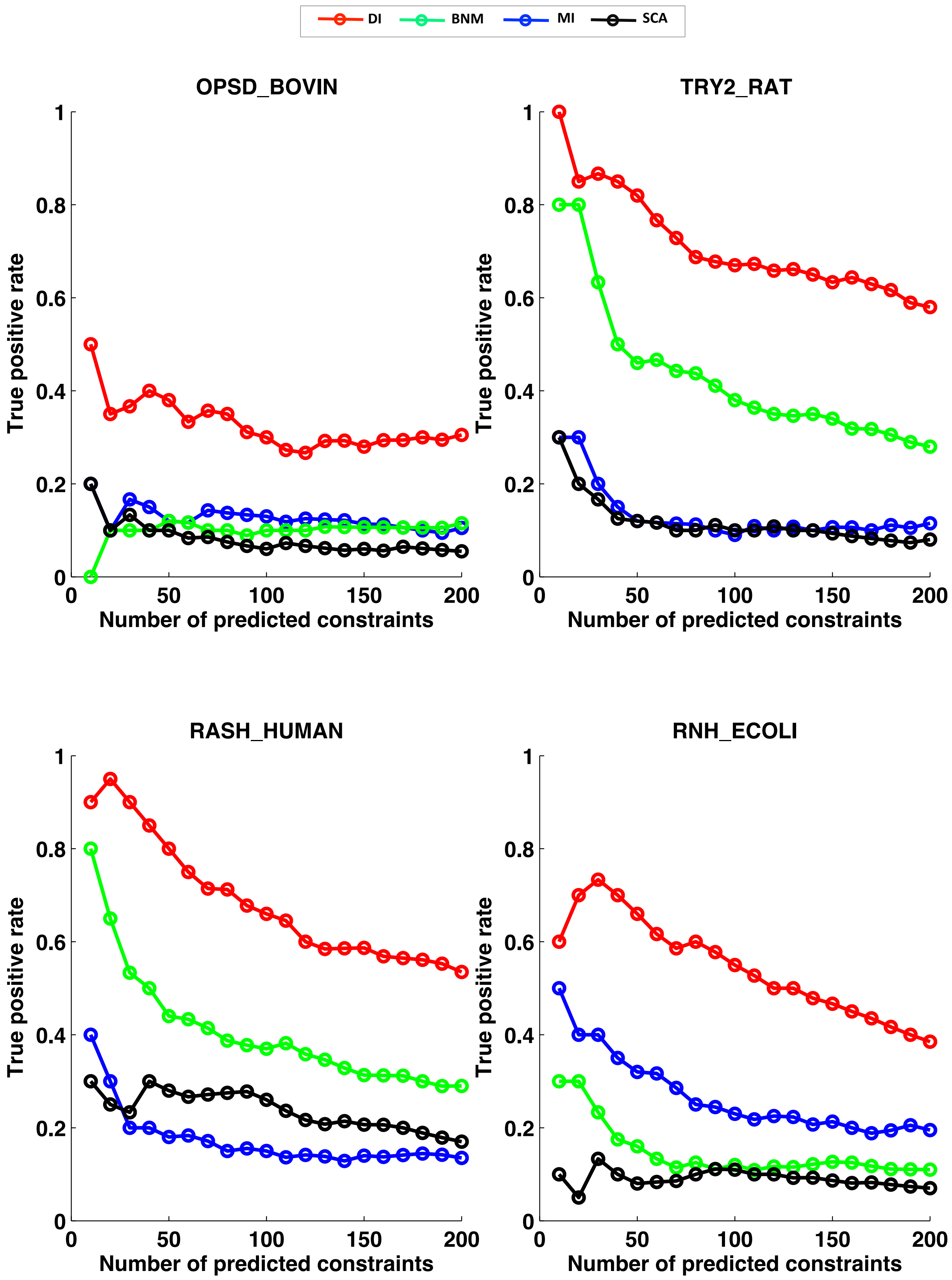

Figure S6. True positive rate of predicted contacts for 4 methods

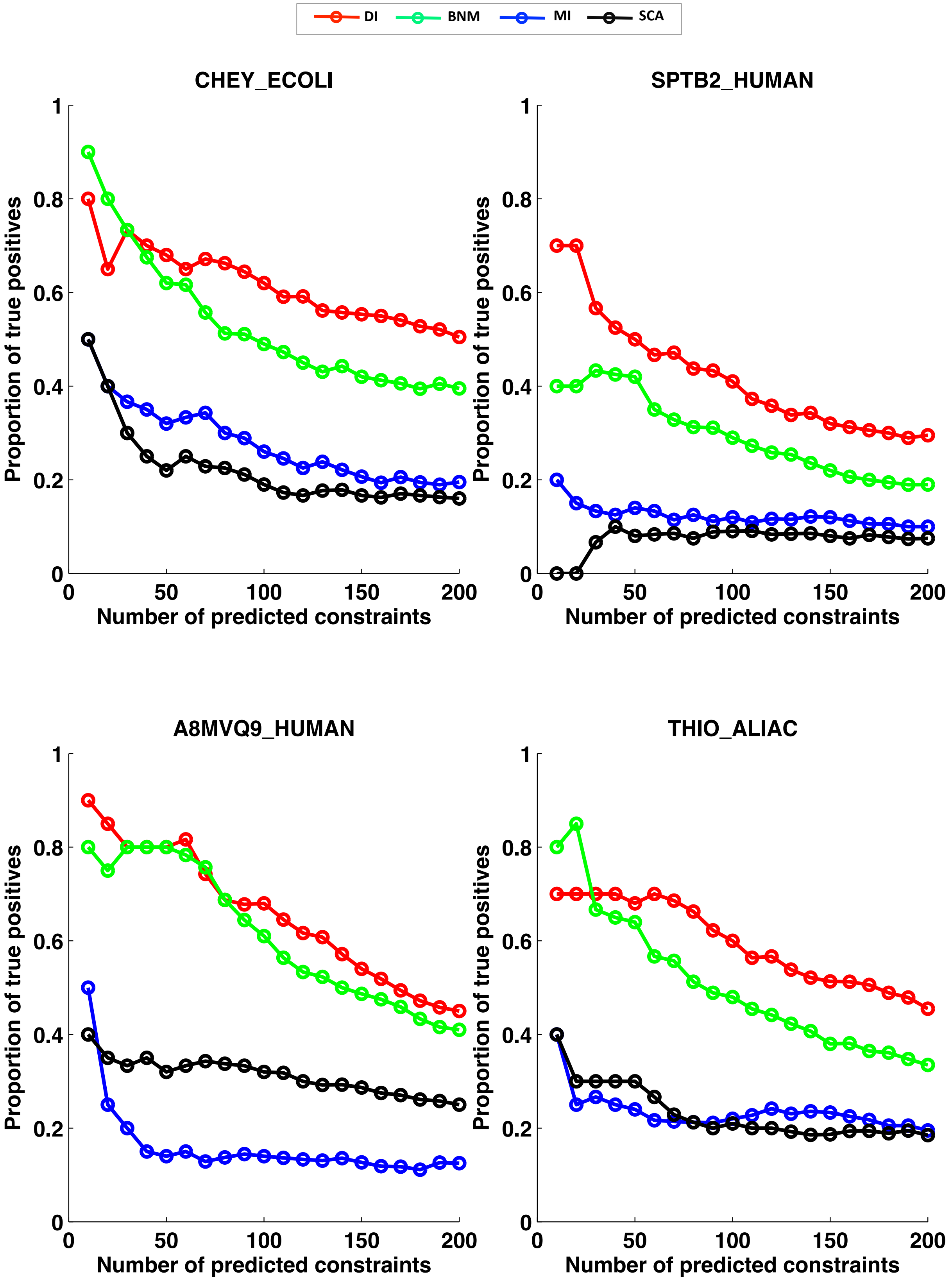

Figure S6. True positive rate of predicted contacts for 4 methods

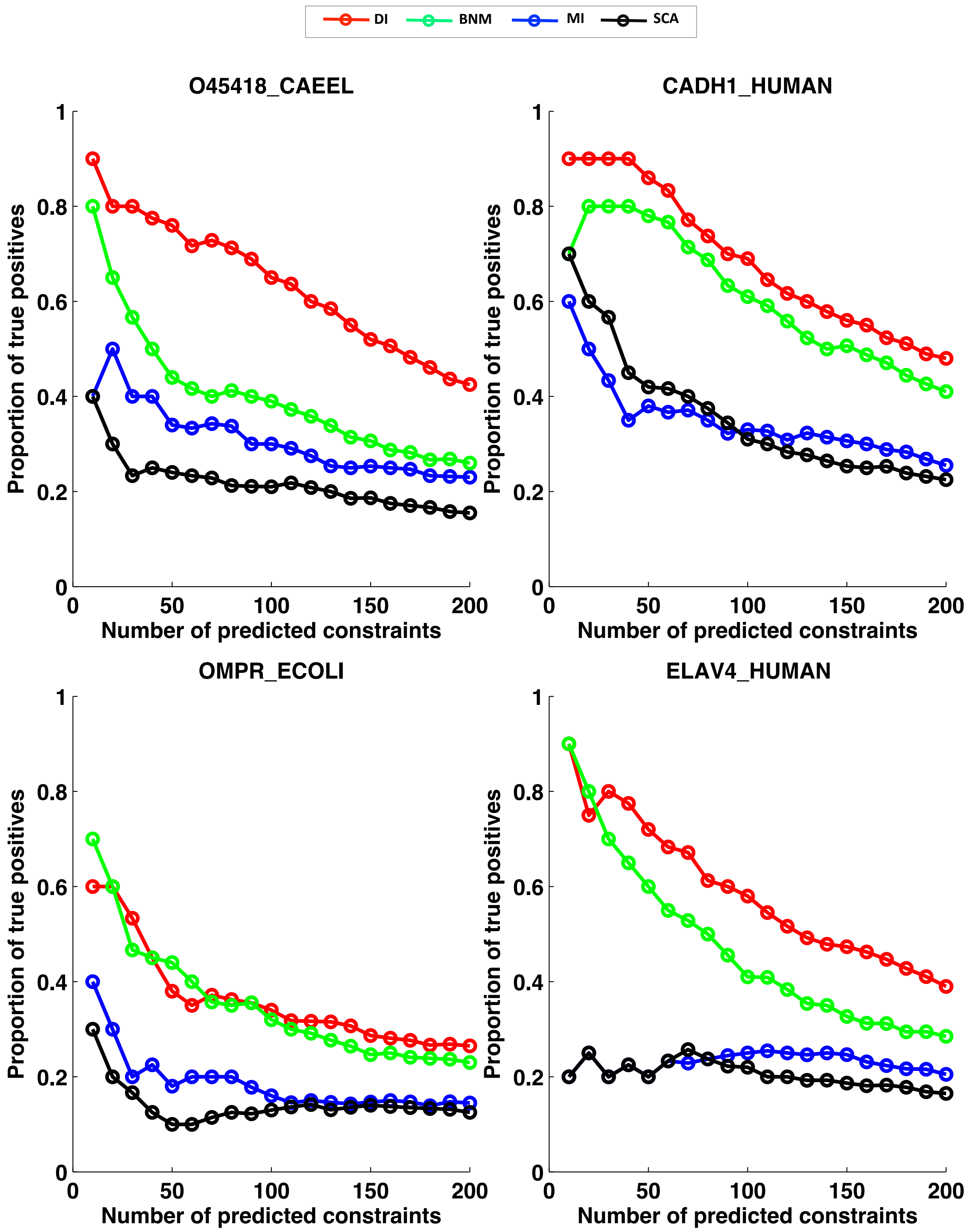

Figure S6. True positive rate of predicted contacts for 4 methods

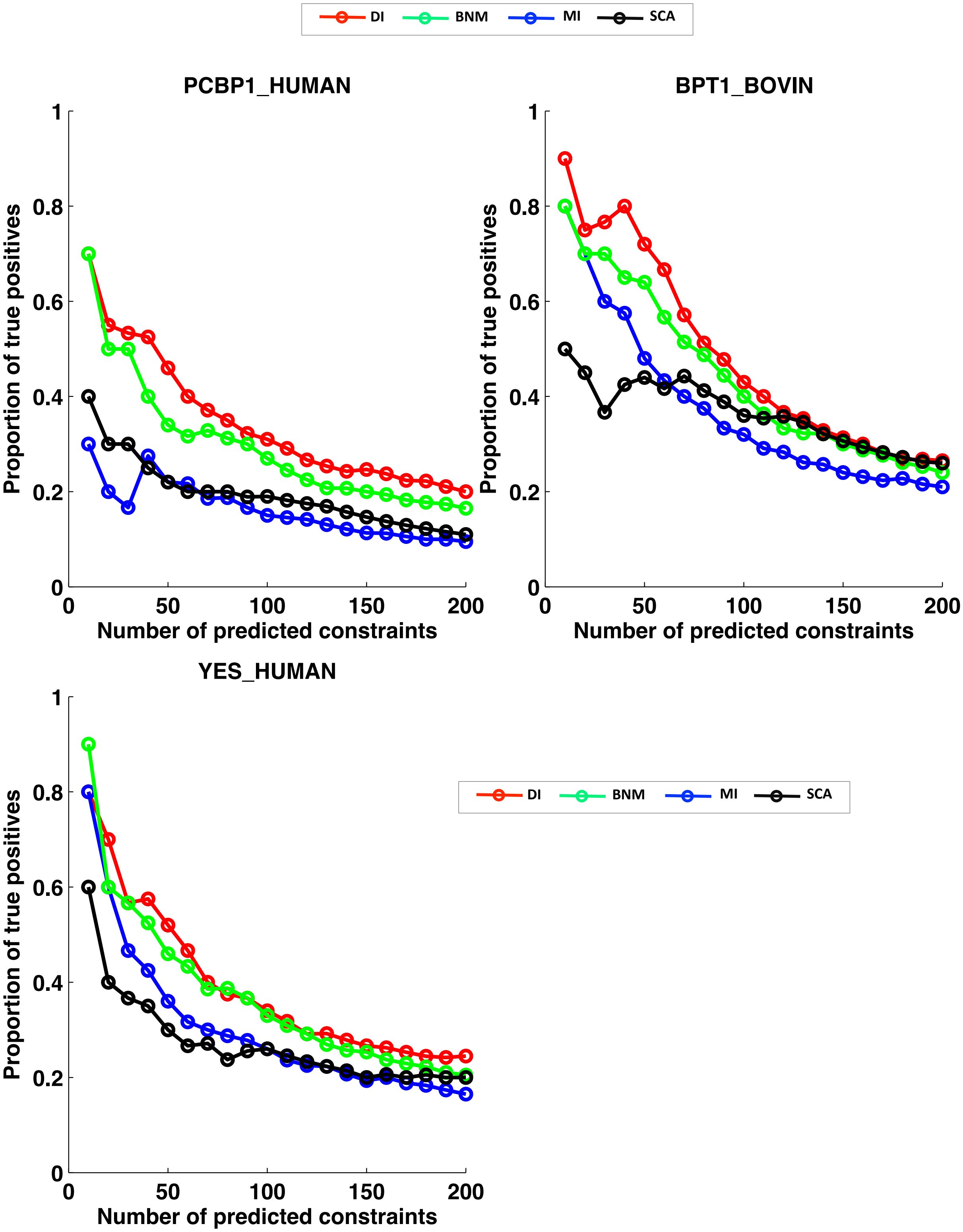

Supplement: Figure S6 — True positive rate of predicted contacts for 4 methods. For each of the 15 proteins, plots show the proportion of true positives over a range of top ranking constraint numbers (10–200) for 4 different contact prediction methods. EIC (DI), this work, shown in red, BNM [13] in green, SCA [61] in black and MI ( our calculation) in blue. True positive is defined as within 5 Å minimum atom distance. Contact predictions from all methods were treated equivalently, with predicted secondary structure clashes, more than one cysteine pairing per cysteine, and >90% conserved residues removed, see Text S1 for pipeline. Although the DI/EIC contacts almost always have the best true positive proportion, the BNM method is favorable in some cases. (PDF) [file pone.0028766.s006.pdf]
