# Supplementary material for: Protein 3D Structure Computed from Evolutionary Sequence Variation
Source: PLoS One. 2011 Dec 7;6(12):e28766. doi: 10.1371/journal.pone.0028766 (PMC3233603; doi:10.1371/journal.pone.0028766)

Figure S7. The minimum atom distance of top 200 ranked DI pairs

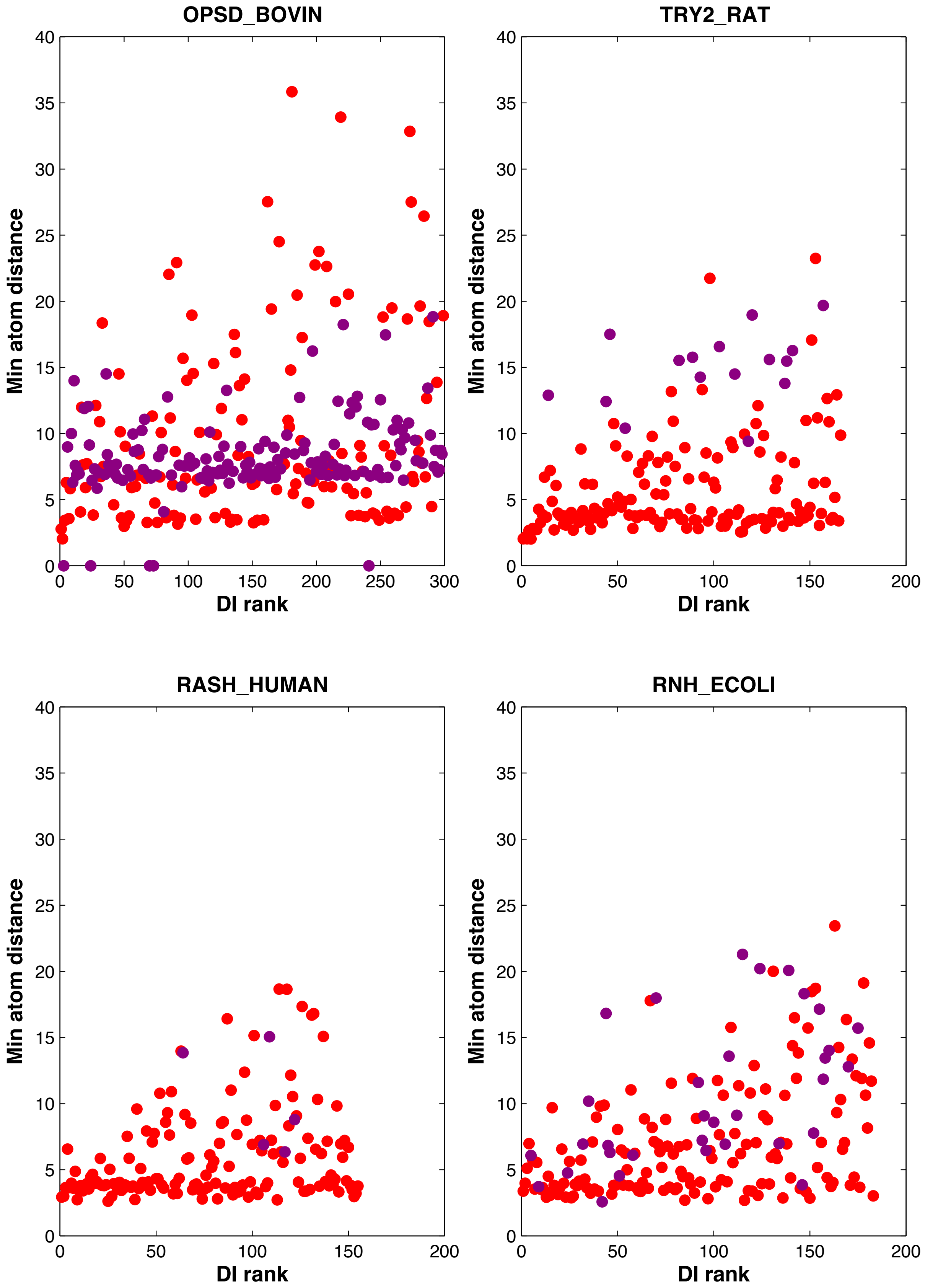

Figure S7. The minimum atom distance of top 200 ranked DI pairs

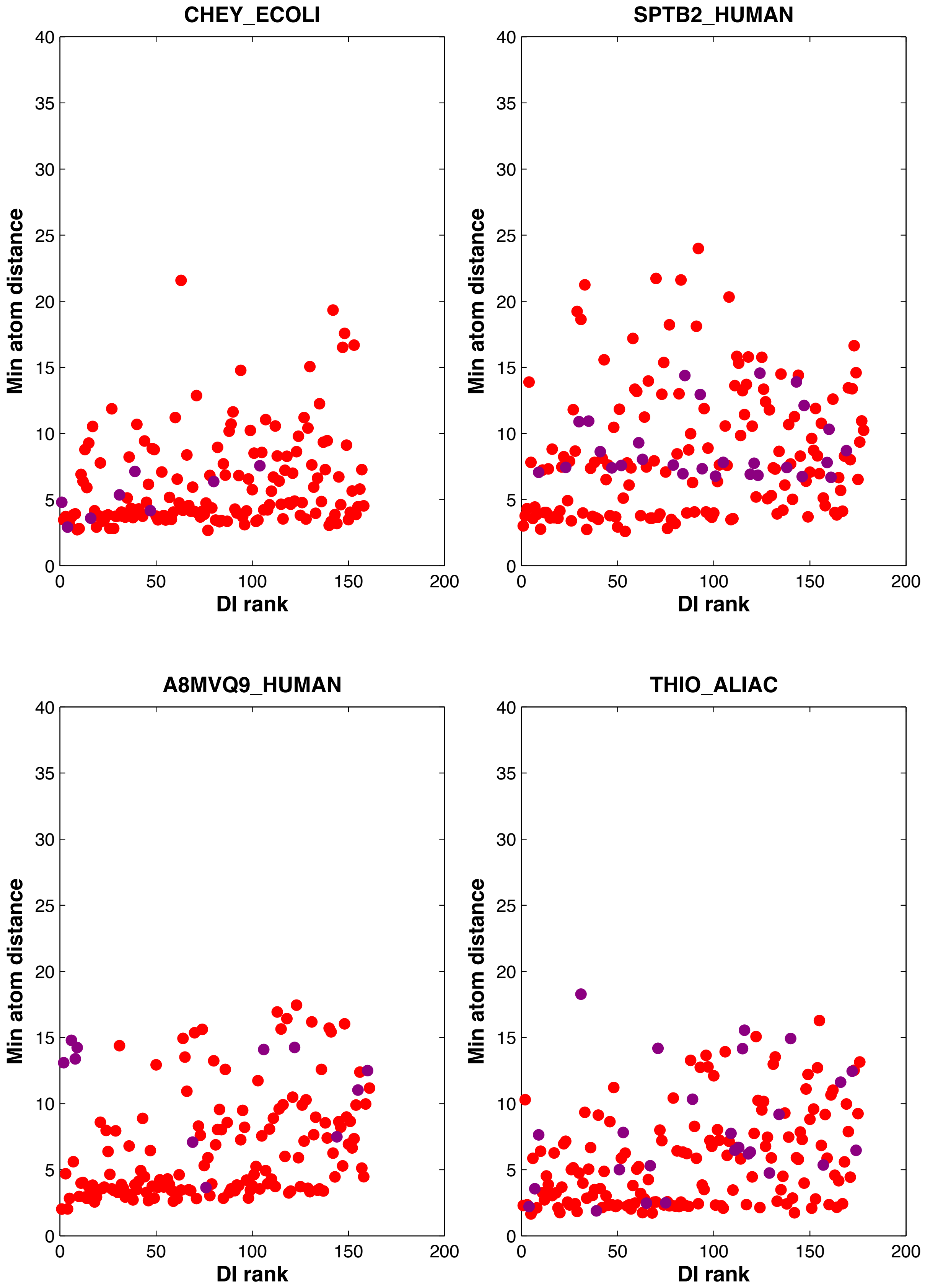

Figure S7. The minimum atom distance of top 200 ranked DI pairs

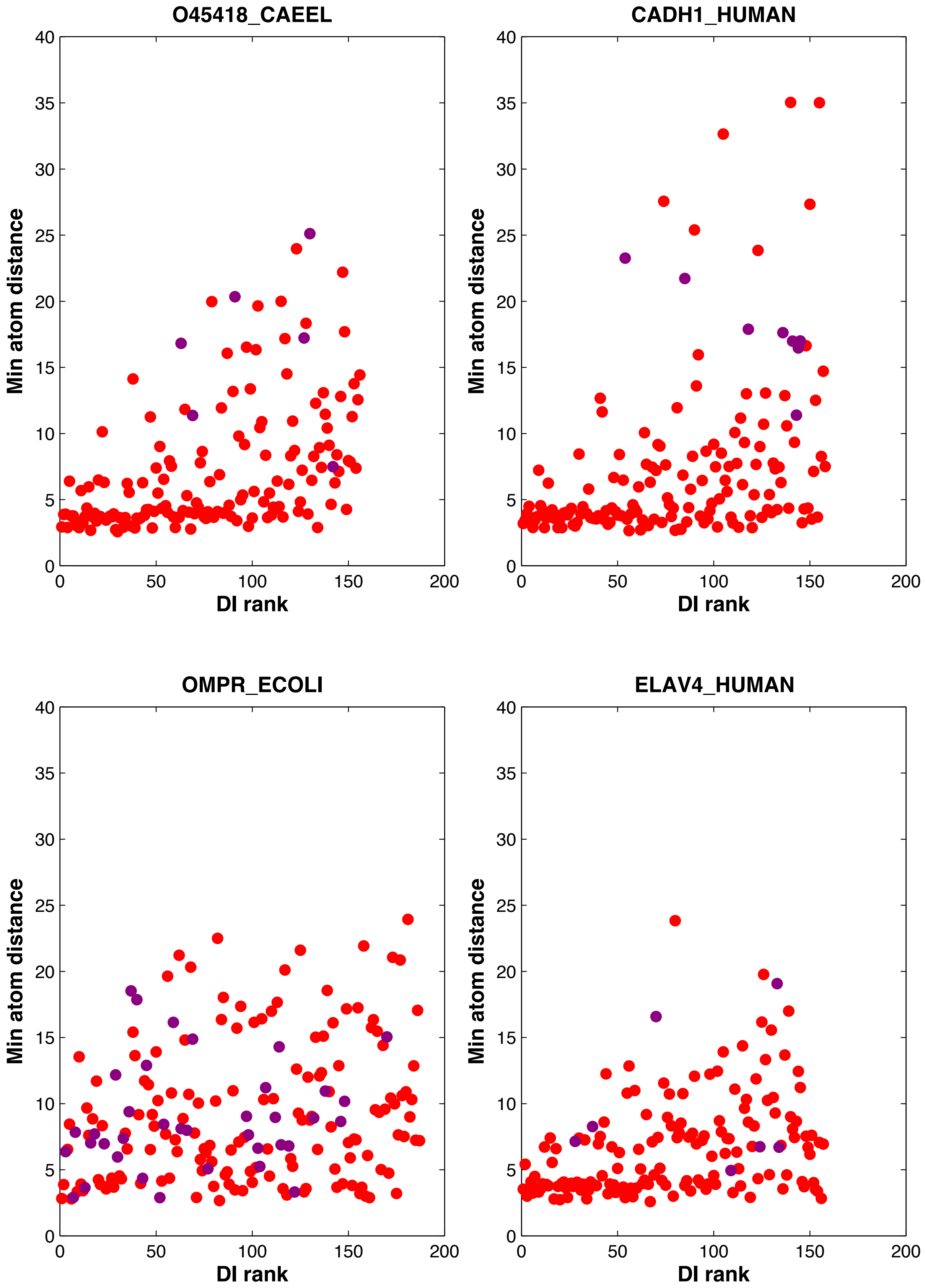

Figure S7. The minimum atom distance of top 200 ranked DI pairs

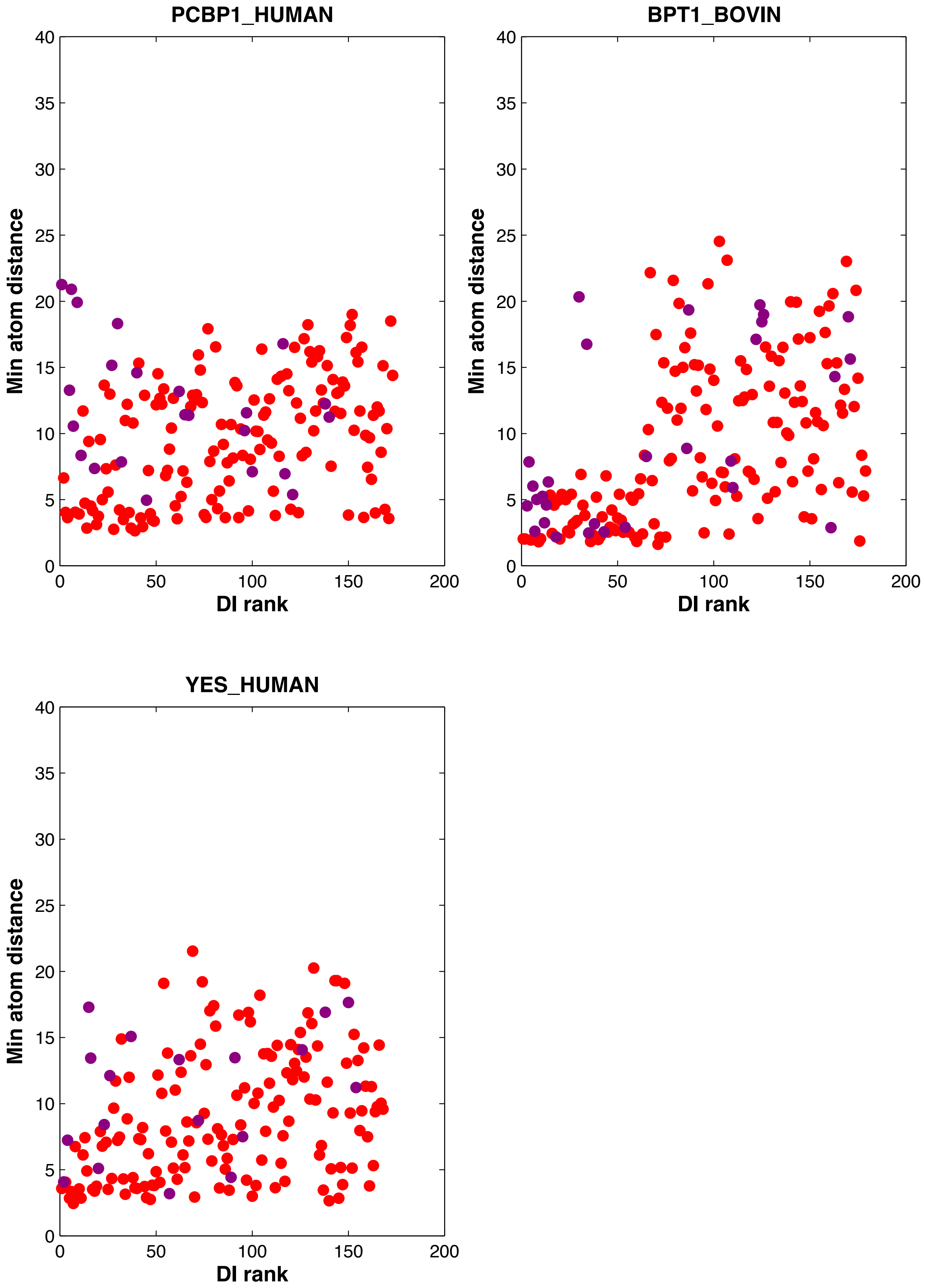

Supplement: Figure S7 — The minimum atom distance of top 200 ranked DI pairs. (4 pages). For each for the 15 proteins, plots show the minimum distance between each DI ranked residue pair. In red are the EICs and in purple the Dis which are filtered by our algorithm. Note that for many proteins, especially A8MVQ9_HUMAN (lectin C ) and Trypsin, high ranking DIs which are false positives are removed from the EICs used for folding, whereas others, for example Ras and Chey are hardly affected, Text S1 and all scores available in Web Appendix A1. Note that the scale changes for each protein. (PDF) [file pone.0028766.s007.pdf]
