# Supplementary material for: Protein 3D Structure Computed from Evolutionary Sequence Variation
Source: PLoS One. 2011 Dec 7;6(12):e28766. doi: 10.1371/journal.pone.0028766 (PMC3233603; doi:10.1371/journal.pone.0028766)

## Figure S8. Relationship between proportion of false positives and 3D structure prediction accuracy

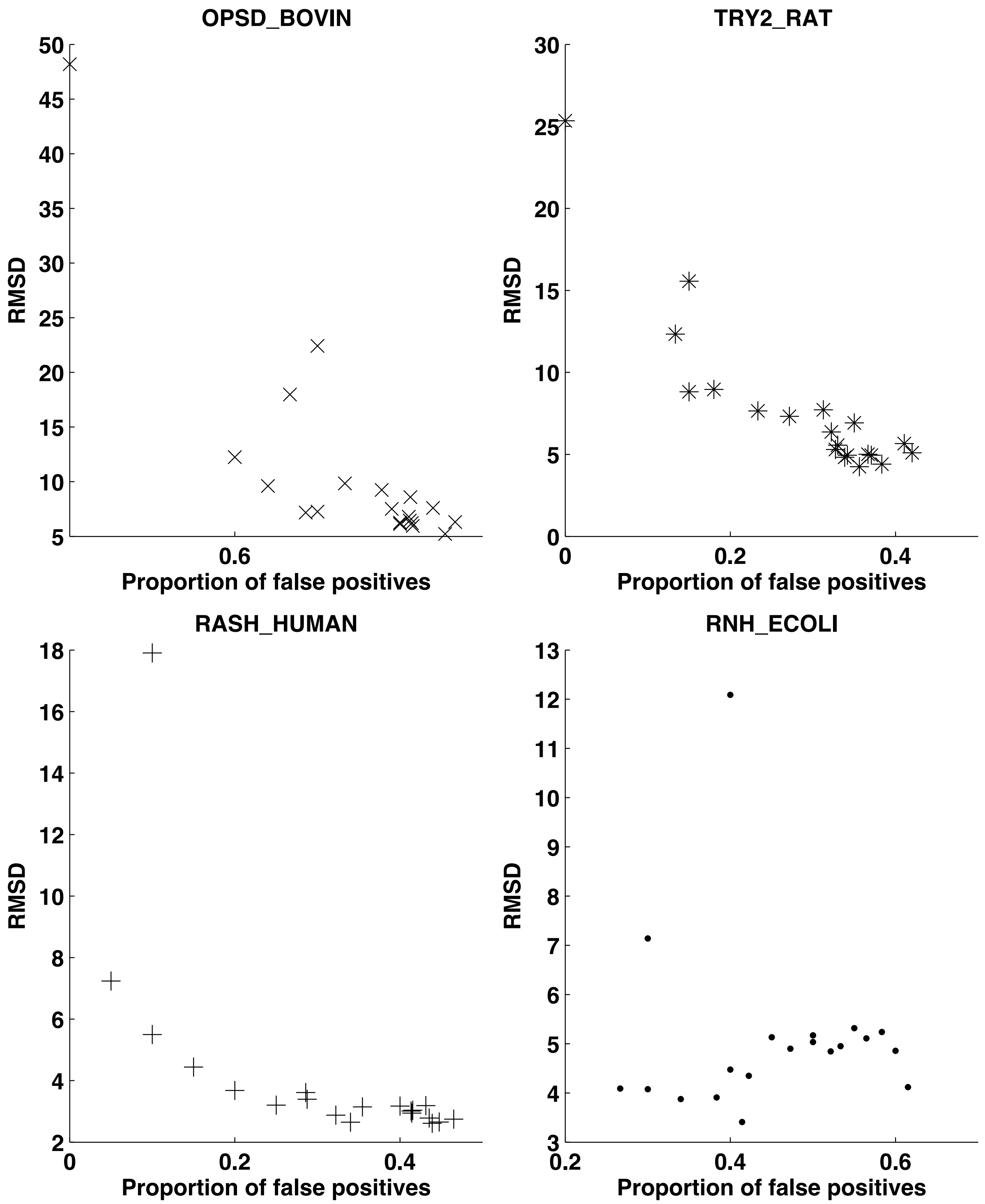

## Figure S8. Relationship between proportion of false positives and 3D structure prediction accuracy

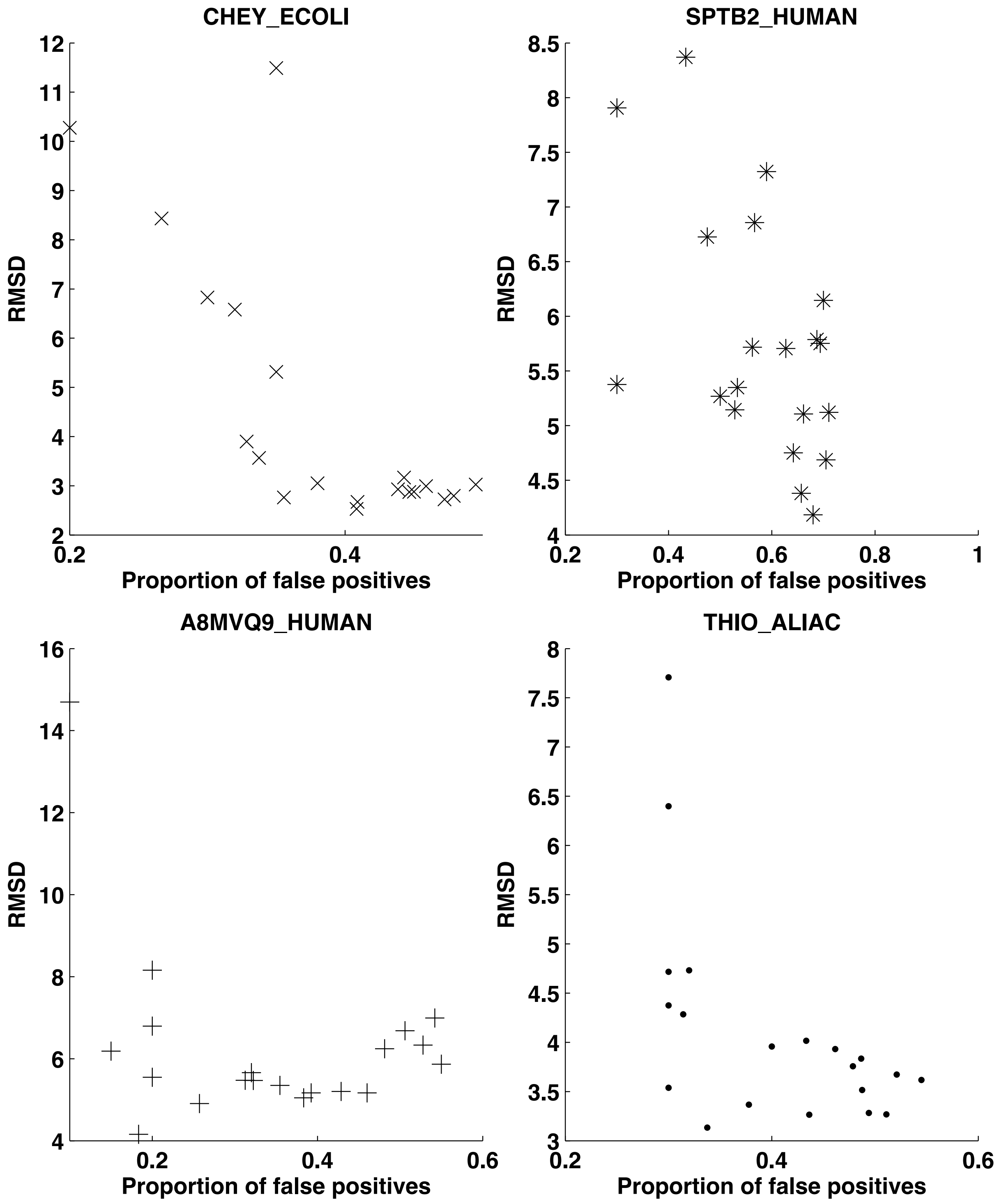

## Figure S8. Relationship between proportion of false positives and 3D structure prediction accuracy

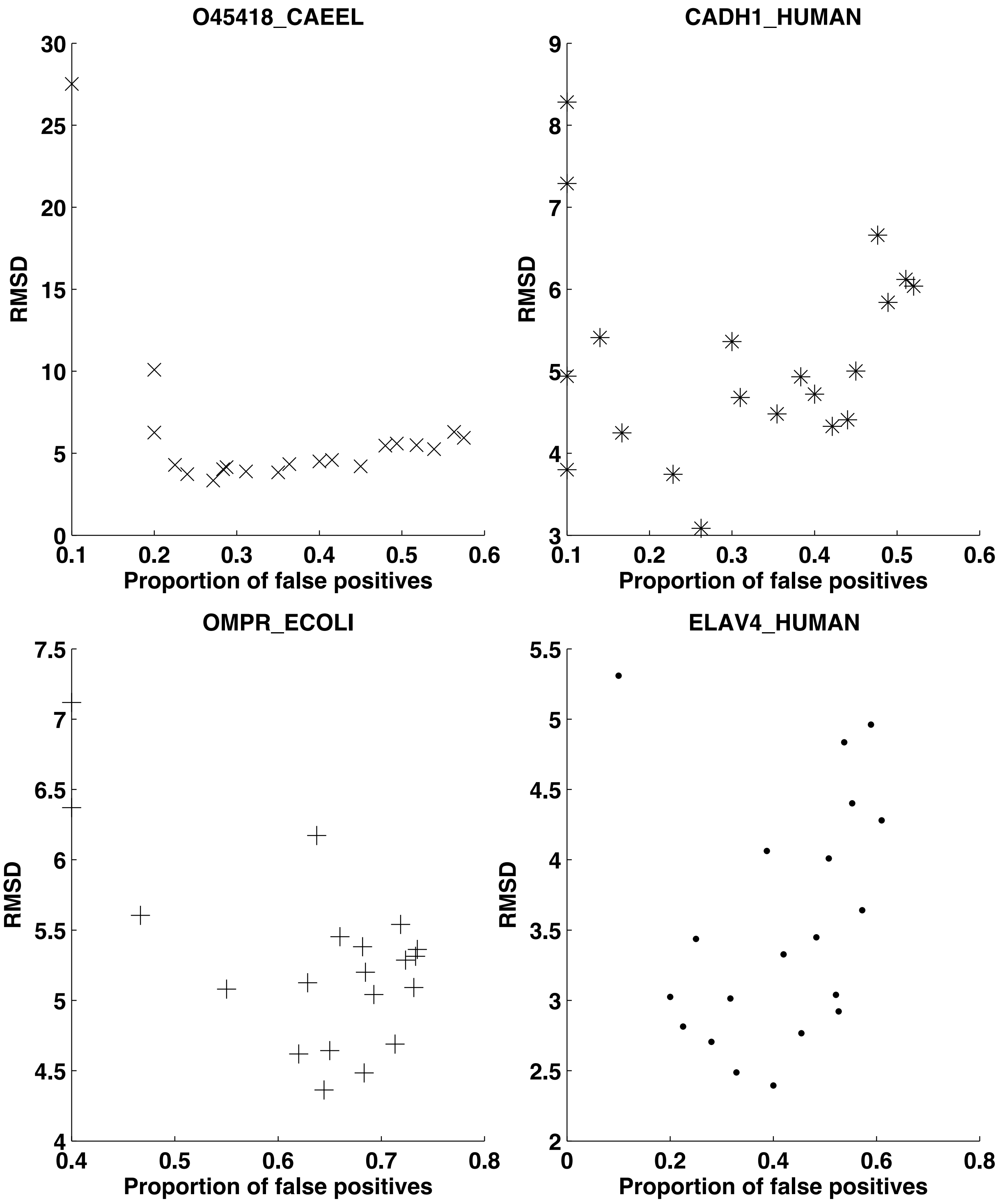

**Figure S8. Relationship between proportion of false positives and 3D structure prediction accuracy**

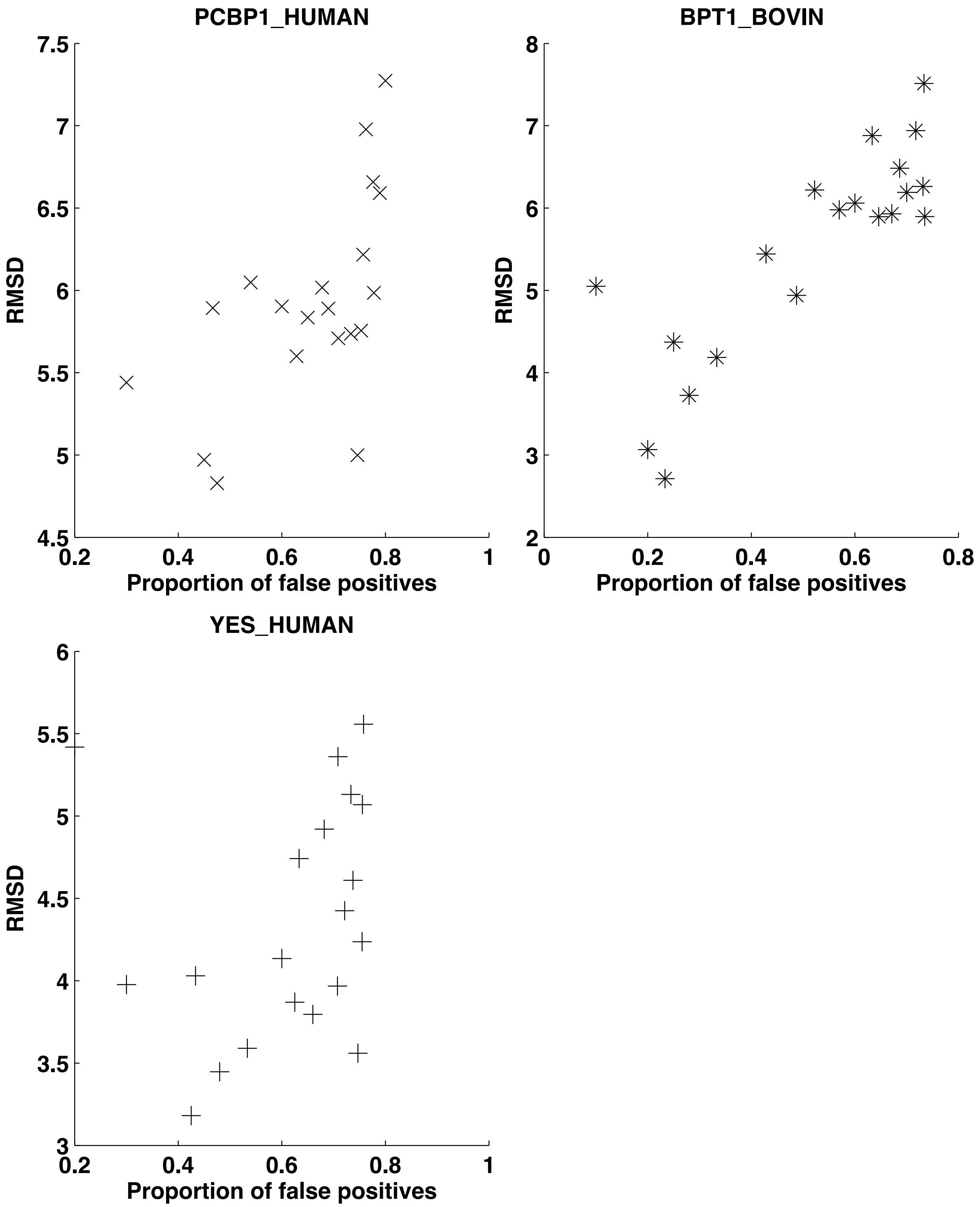

Supplement: Figure S8 — Relationship between proportion of false positives and 3D structure prediction accuracy. (4 pages). For all 15 proteins, comparison of the proportion of false positives in 20 sets of constraint numbers ranging from 10–200, compared to the best Cα-RMSD accuracy for a structure predicted using the same number of EIC constraints. Some proteins such as the SH3 domain of YES and trypsin inhibitor, how a clear decline in best predicted structure accuracy with increasing proportion of false positive contact and others such as CheY show the inverse relationship. However, those that show an inverse relationship tends to have a lower rate of FPs overall and all proteins show best accuracy at FP proportion below 0.4. (PDF) [file pone.0028766.s008.pdf]
