# Supplementary material for: Protein 3D Structure Computed from Evolutionary Sequence Variation
Source: PLoS One. 2011 Dec 7;6(12):e28766. doi: 10.1371/journal.pone.0028766 (PMC3233603; doi:10.1371/journal.pone.0028766)

Figure S9. Quantitative false positive assessment

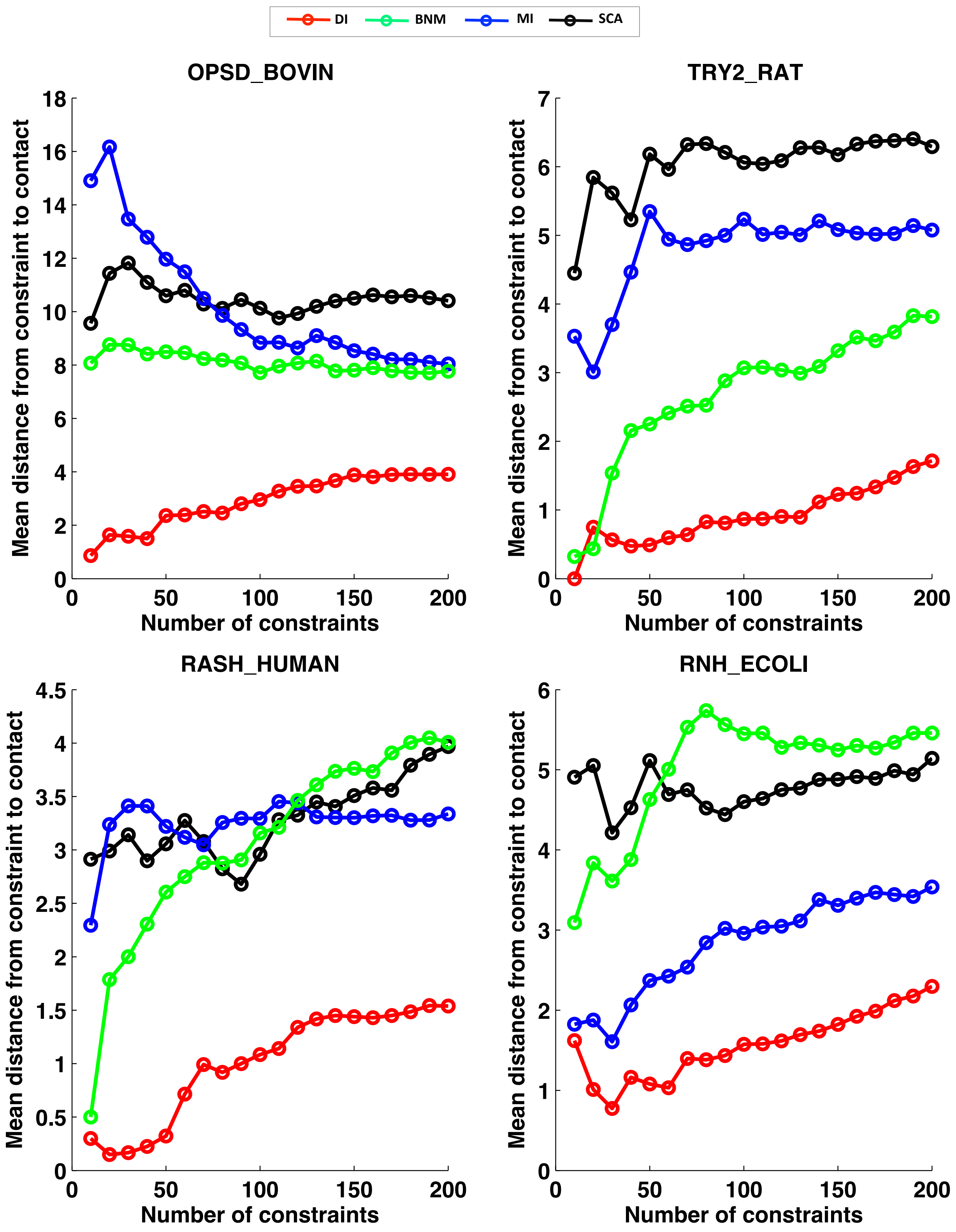

Figure S9. Quantitative false positive assessment

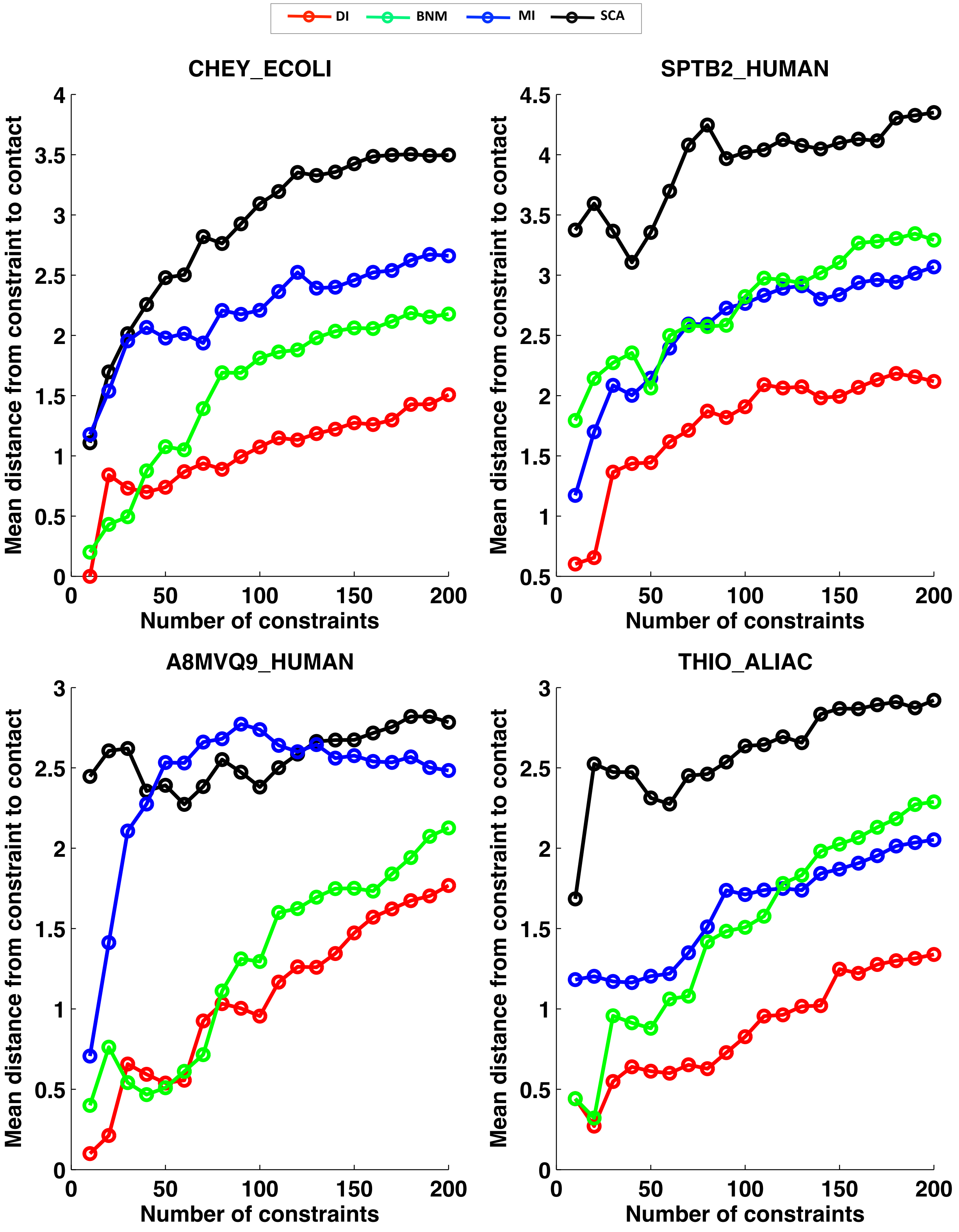

Figure S9. Quantitative false positive assessment

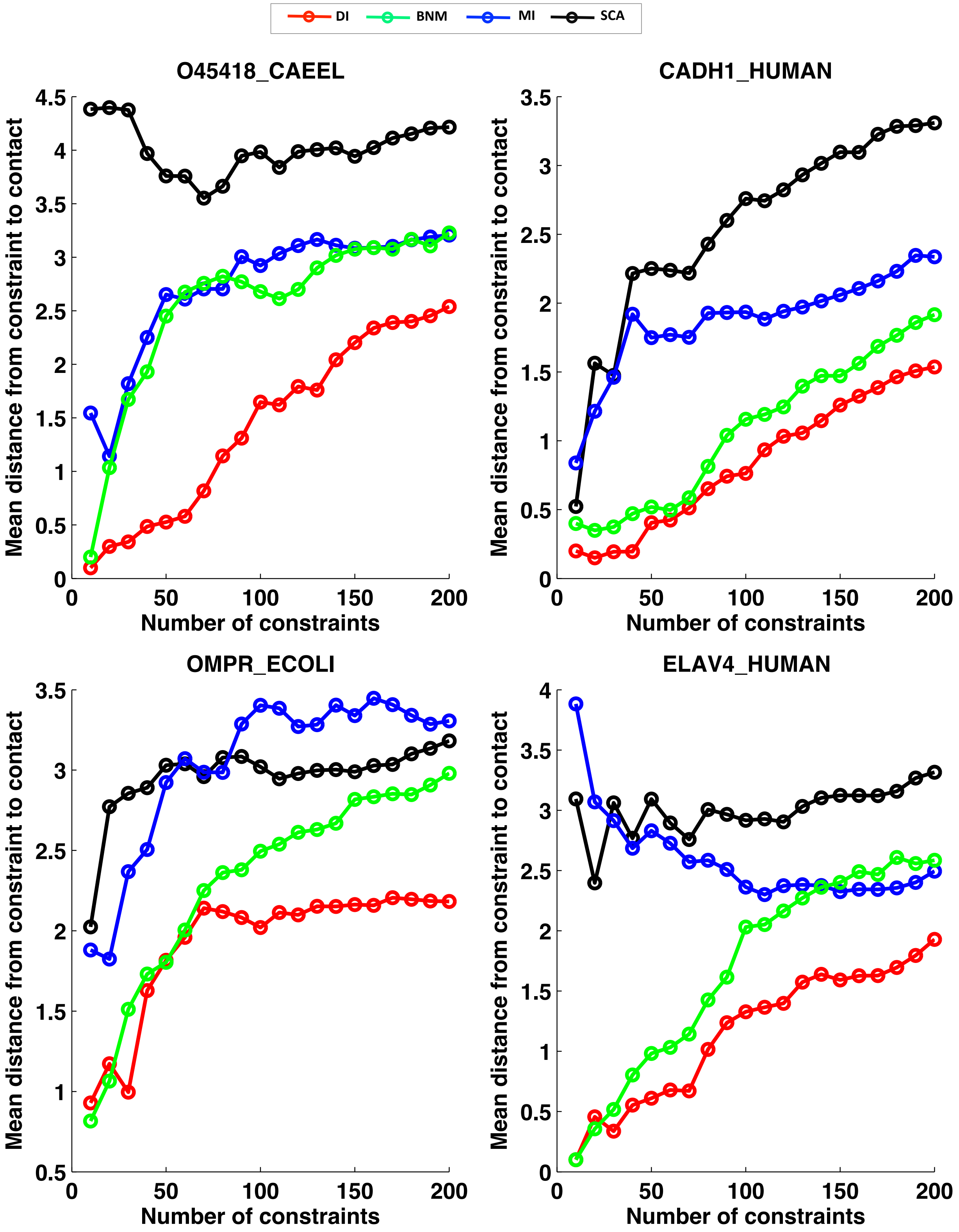

Figure S9. Quantitative false positive assessment

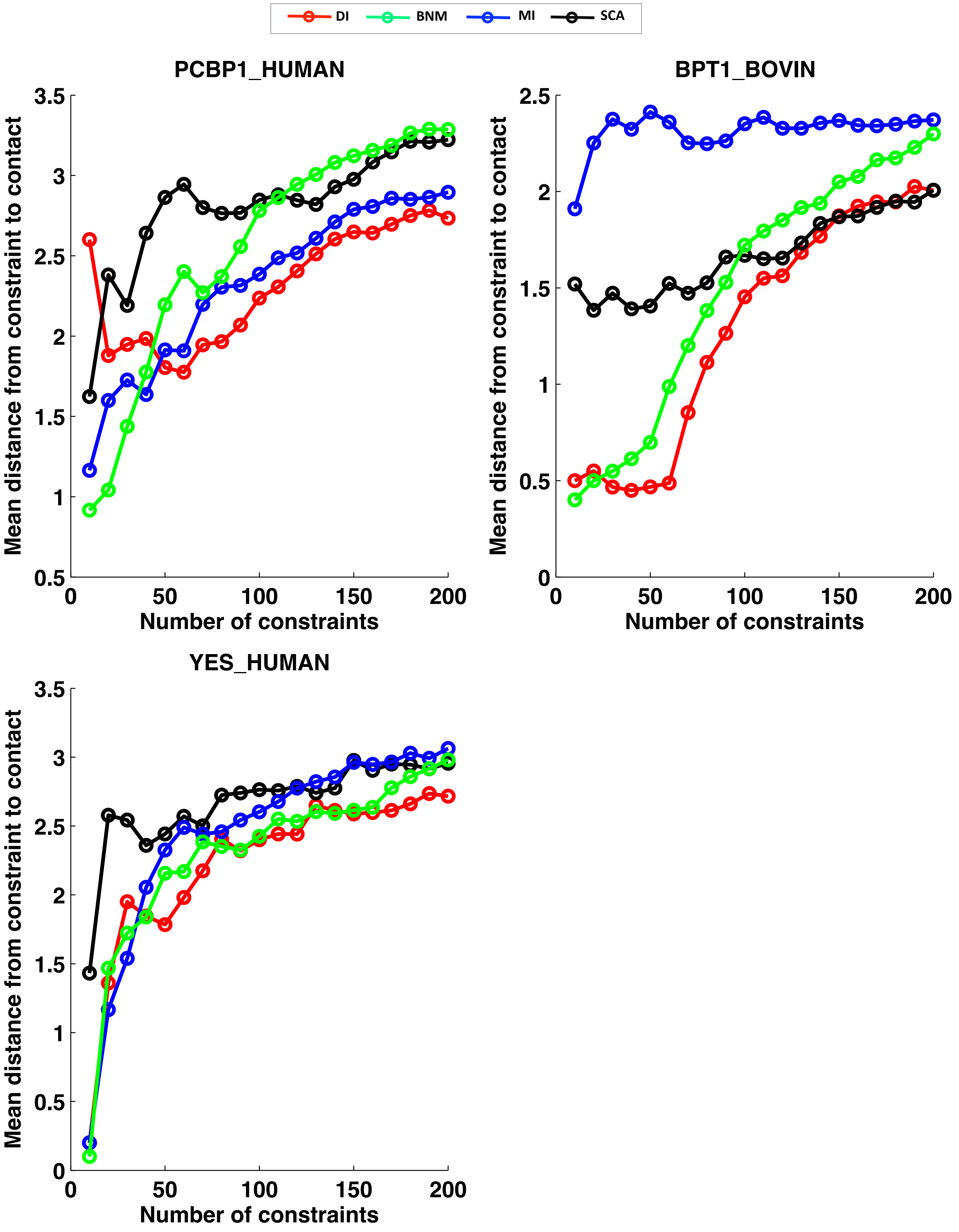

Supplement: Figure S9 — Quantitative false positive assessment. (4 pages). Since a false positive calculation is typically is limited to a binary count (it is counted as either a false positive or not), we developed a metric to compare how far ‘wrong’ the FPs are. For each predicted EIC constraint, in N-scoring residue pairs (10–200) we calculate the Euclidean 2D distance to the nearest contact in the crystal structure and report the mean of this distance for each Nc, over all 15 proteins. This is repeated for each of the other contact predicted methods, MI, BNM and SCA. Red, DI: blue, MI; green, BNM: black SCA. (PDF) [file pone.0028766.s009.pdf]
