# Supplementary material for: Protein 3D Structure Computed from Evolutionary Sequence Variation
Source: PLoS One. 2011 Dec 7;6(12):e28766. doi: 10.1371/journal.pone.0028766 (PMC3233603; doi:10.1371/journal.pone.0028766)

Figure S10. Quantitative assessment of spread of predicted contacts

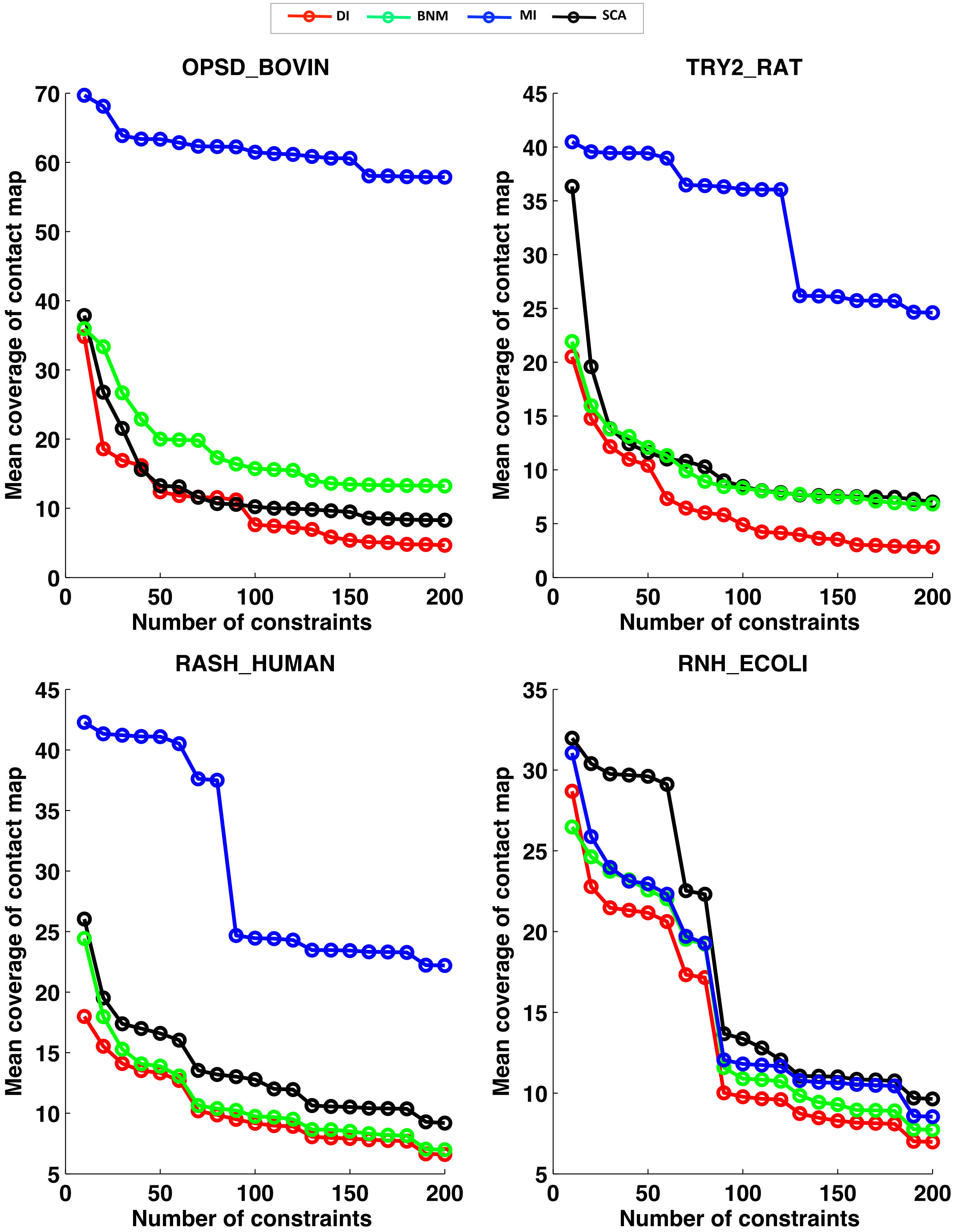

Figure S10. Quantitative assessment of spread of predicted contacts

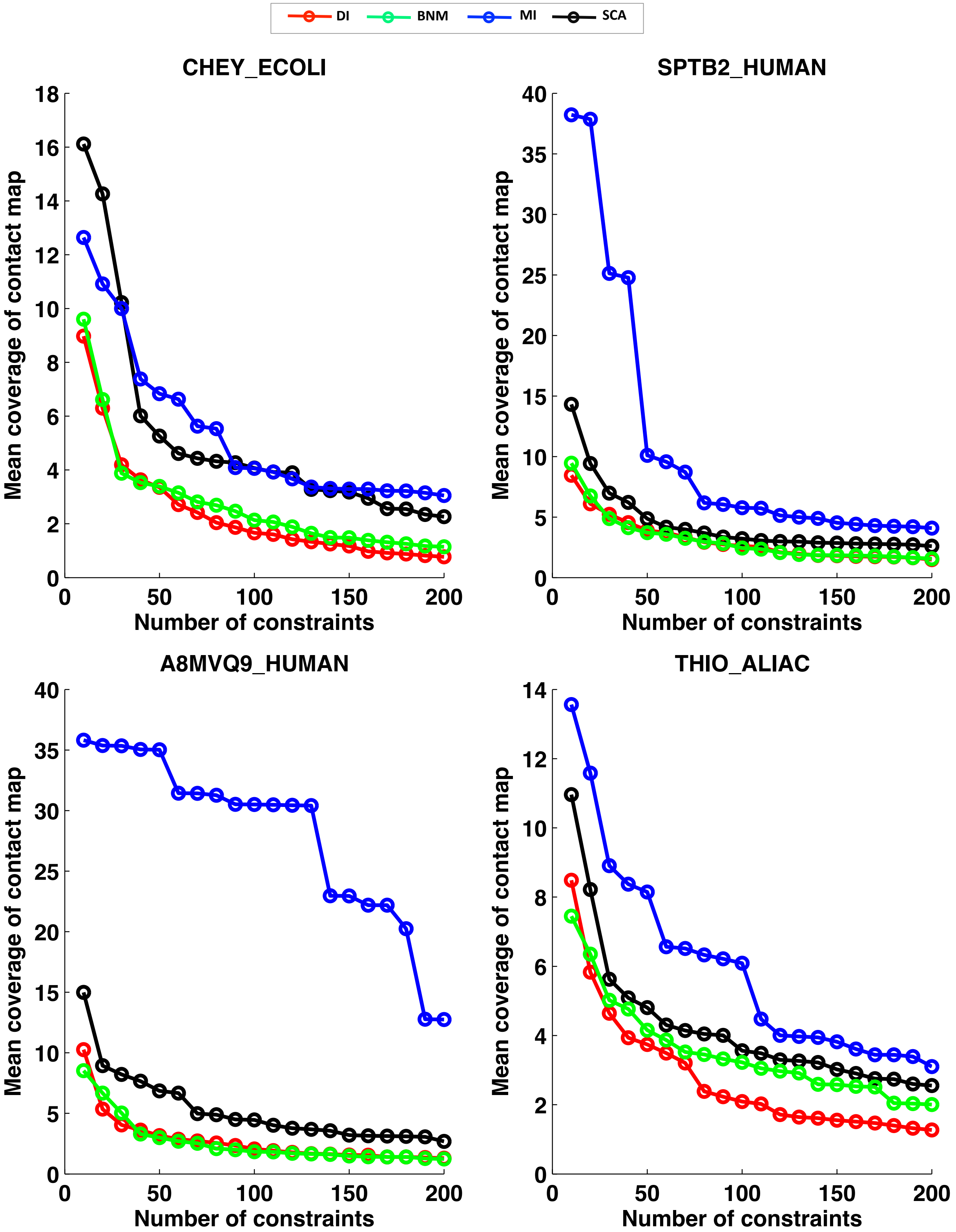

Figure S10. Quantitative assessment of spread of predicted contacts

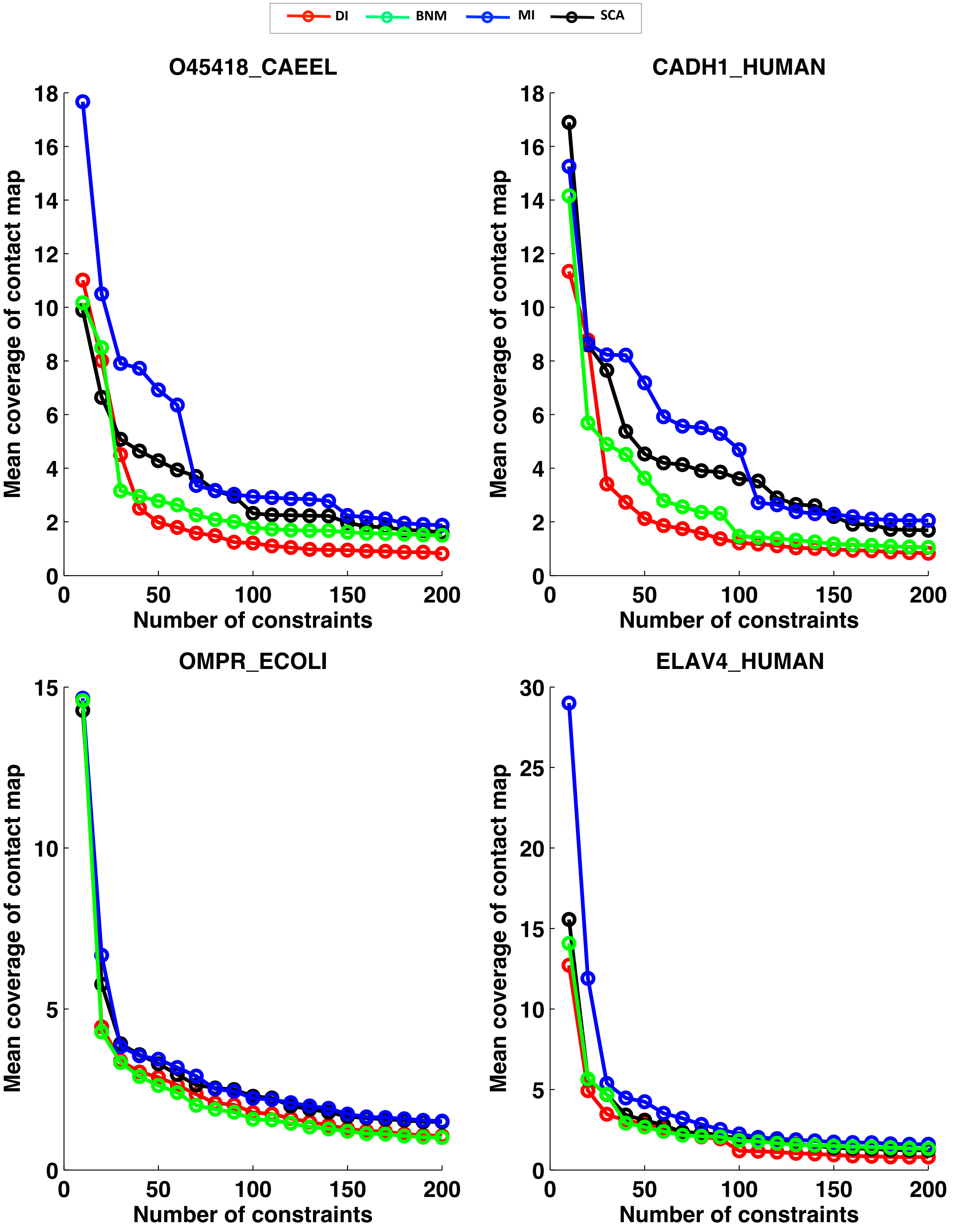

Figure S10. Quantitative assessment of spread of predicted contacts

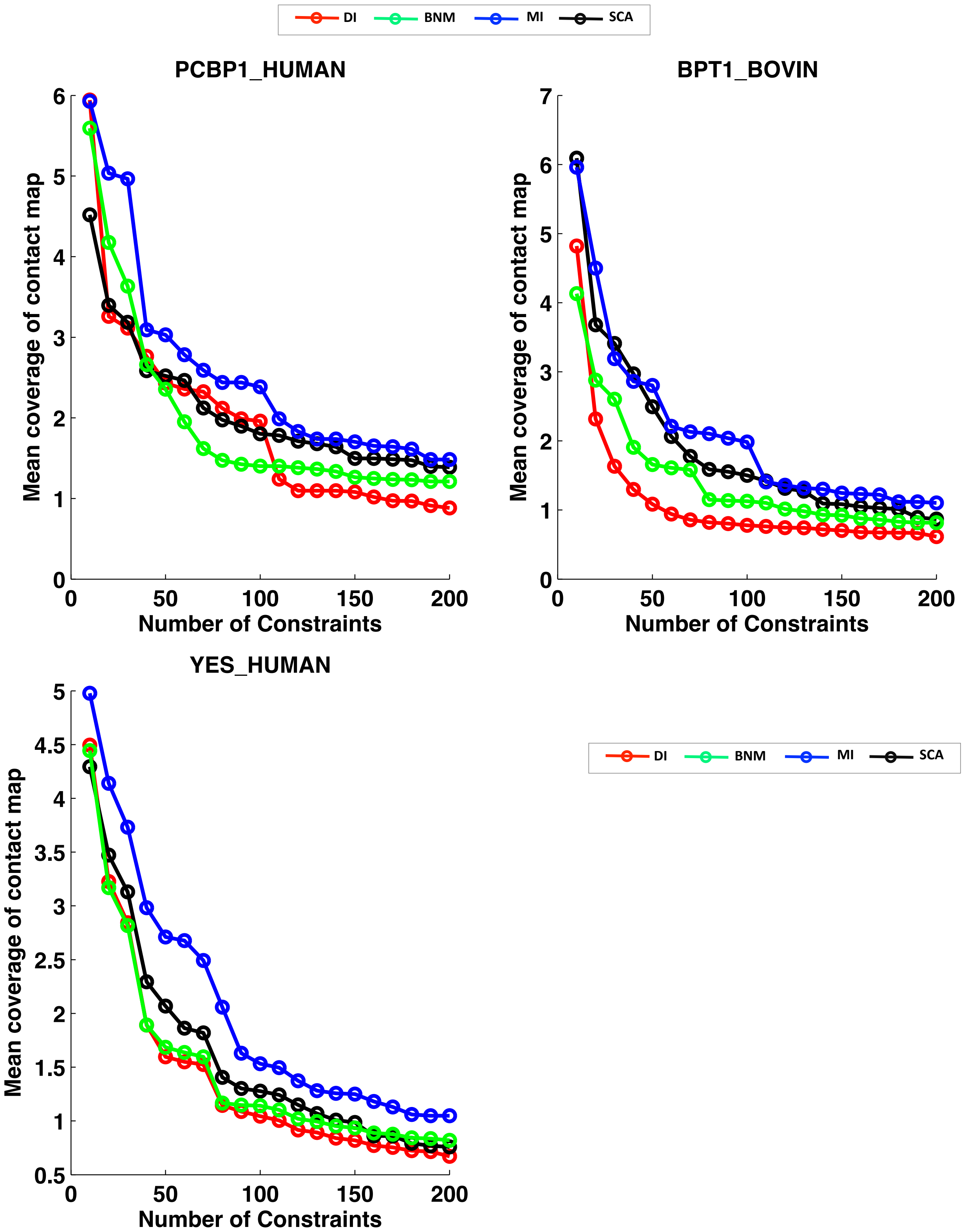

Supplement: Figure S10 — Quantitative assessment of spread of predicted contacts (4 pages). True positive counts alone do not reflect how well-distributed the top N-scoring pairs are across the protein. Therefore we developed a metric to measure how well the top N-scoring residue pairs ‘cover’ the contact map of a corresponding crystal structure. We compute the Euclidean 2D distance between the contact map of the corresponding crystal structure, and the contact map consisting of the top N-scoring residue pairs. For each residue pair, separated by more than five residues in sequence, we compute the distance to the nearest high-scoring residue pair (for instance, the nearest ‘red star’ in the contact map, in the case of EIC pairs). For each set of N-scoring residue pairs we calculate the mean of the distances for all contacts to the nearest contact in the crystal structure. Plotted is the mean spread for each Nc for 4 methods, across all 15 proteins. Red, DI: blue, MI; green, BNM: black SCA. (PDF) [file pone.0028766.s010.pdf]
