# Supplementary material for: Protein 3D Structure Computed from Evolutionary Sequence Variation
Source: PLoS One. 2011 Dec 7;6(12):e28766. doi: 10.1371/journal.pone.0028766 (PMC3233603; doi:10.1371/journal.pone.0028766)

Figure S11. Bayesian Network Model (BNM) contact maps.

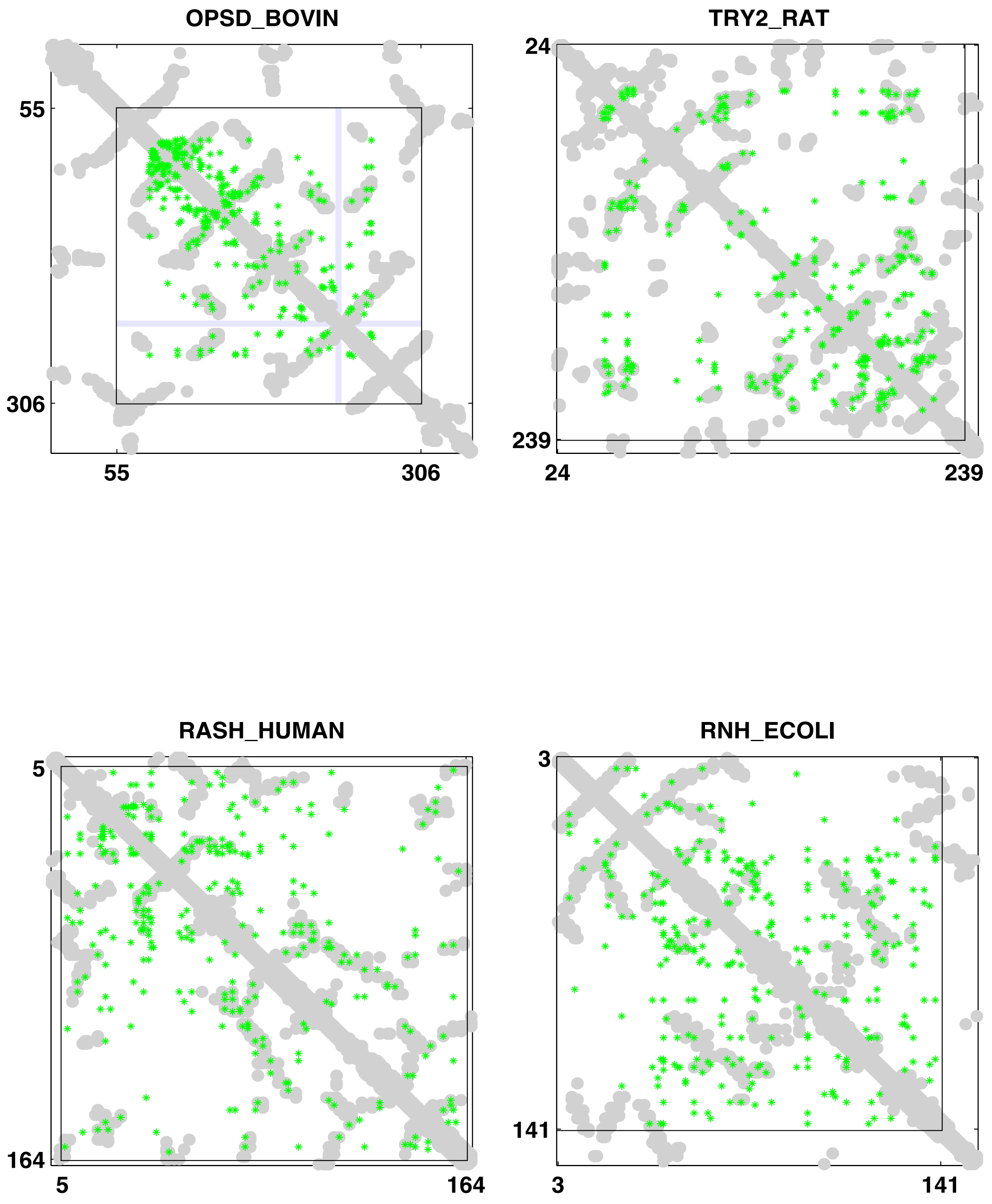

Figure S11. Bayesian Network Model (BNM) contact maps.

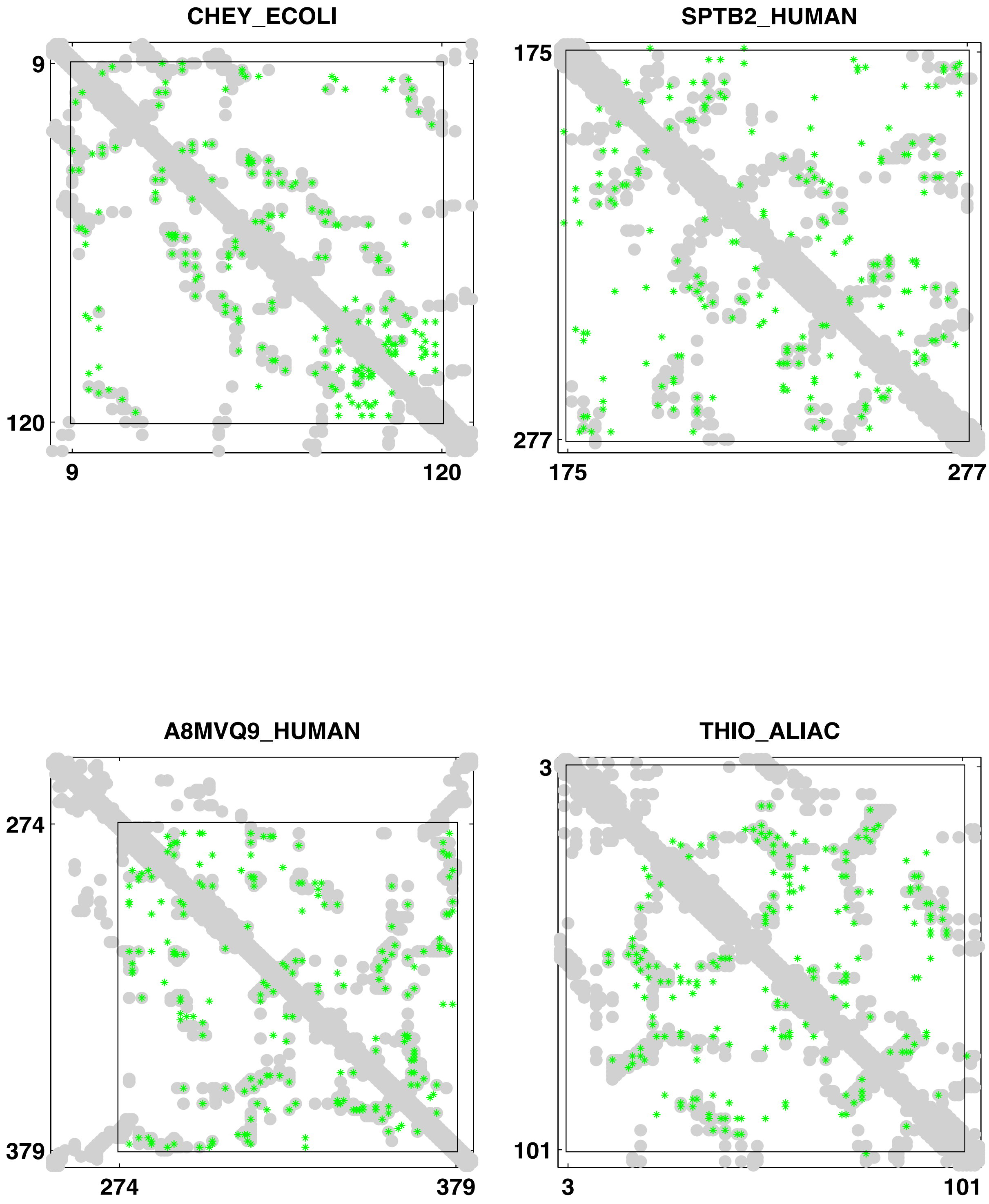

Figure S11. Bayesian Network Model (BNM) contact maps.

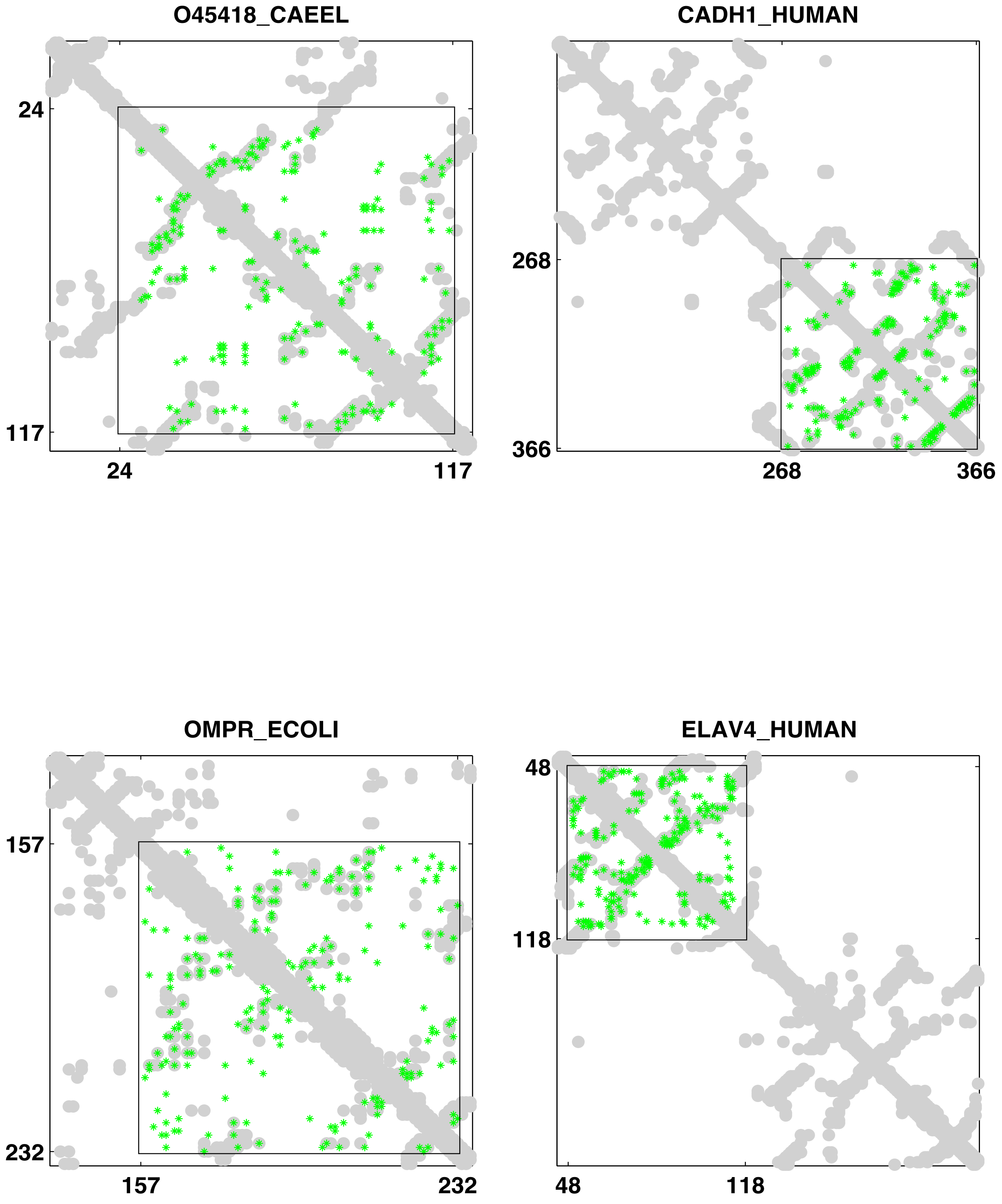

Figure S11. Bayesian Network Model (BNM) contact maps.

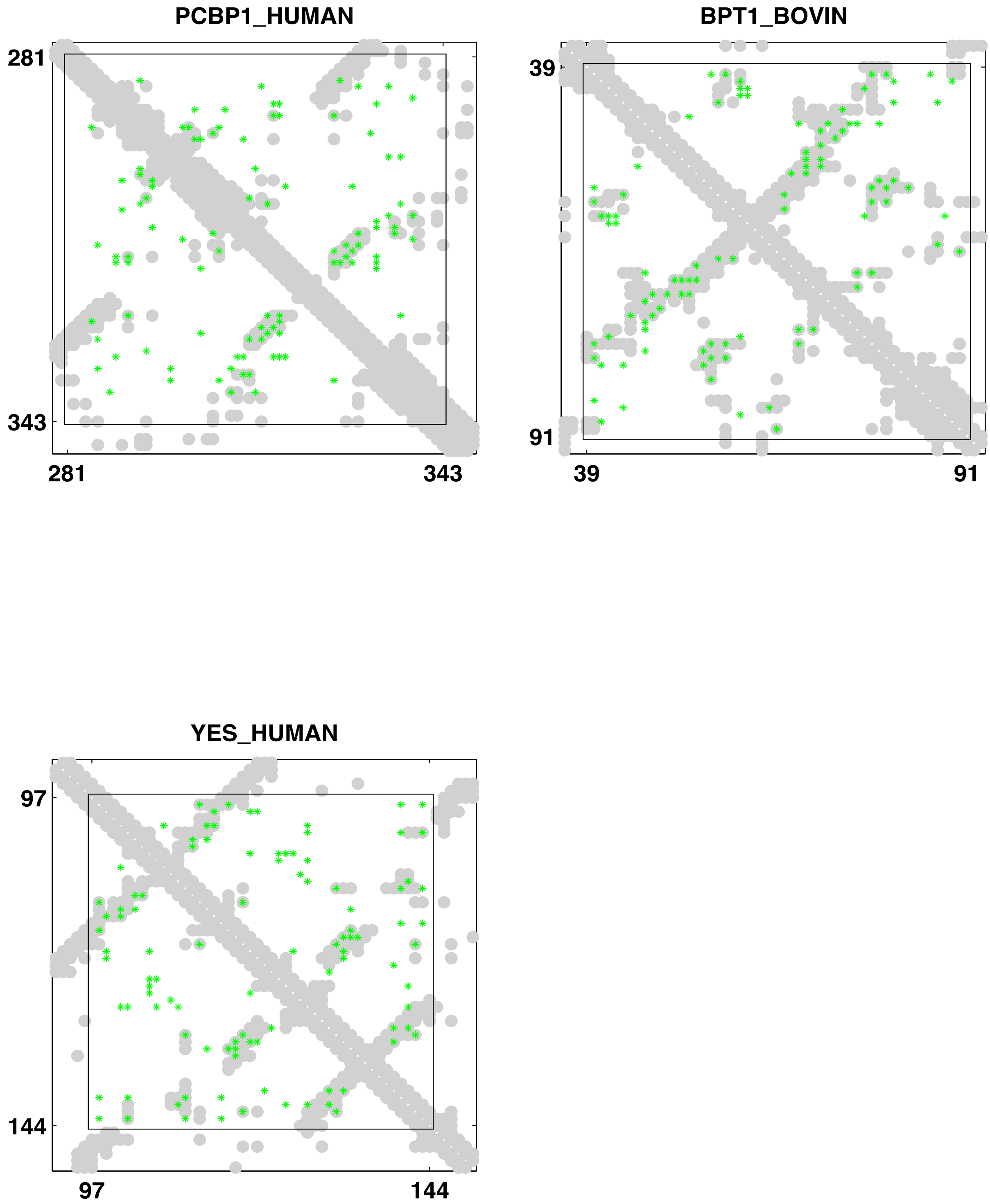

Supplement: Figure S11 — Bayesian Network Model (BNM) contact maps. (4 pages). Predicted contacts (blue dots) from high-ranking BNM scores excluding clashes with secondary structure prediction (see Text S1) and residues pairs 5 or less apart in the polypeptide chain. BNM predicted contacts overlaid onto contacts made in the corresponding crystal structure (grey circles), names as in Table 1. Contacts defined as 5 Å or less from any atom between the paired residues. Number of top-ranked BNM contacts shown sorted into 4 groups: page 1, 150 (larger proteins); pages 2 and 3, 100 (medium size proteins); page 4 (smaller proteins), 50. BNM ranked scores of residue couplings are available in Web Appendix A8. (PDF) [file pone.0028766.s011.pdf]
