# Supplementary material for: Protein 3D Structure Computed from Evolutionary Sequence Variation
Source: PLoS One. 2011 Dec 7;6(12):e28766. doi: 10.1371/journal.pone.0028766 (PMC3233603; doi:10.1371/journal.pone.0028766)

Figure S13. The minimum atom distance of top 200 ranked MI pairs.

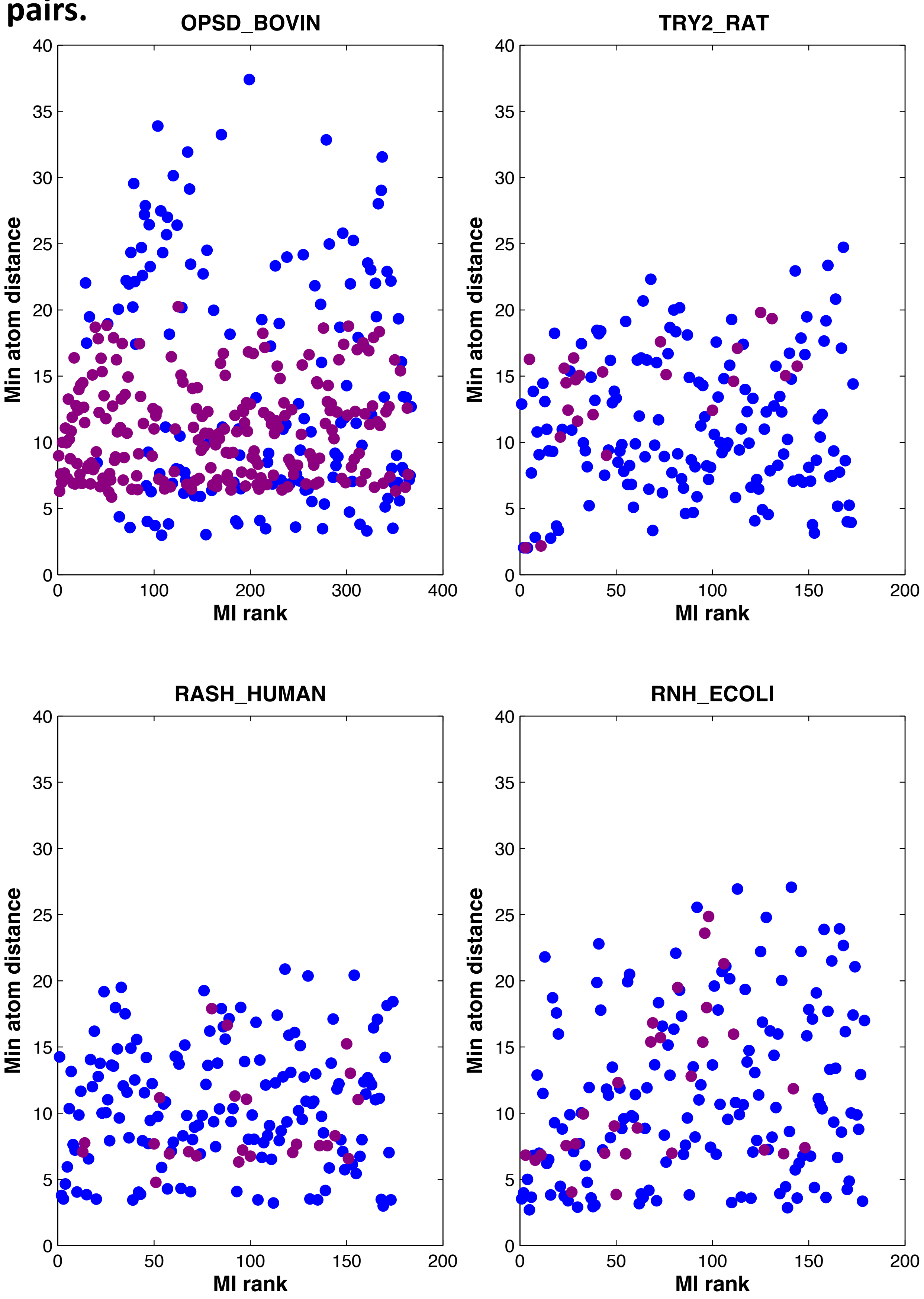

Figure S13. The minimum atom distance of top 200 ranked MI pairs.

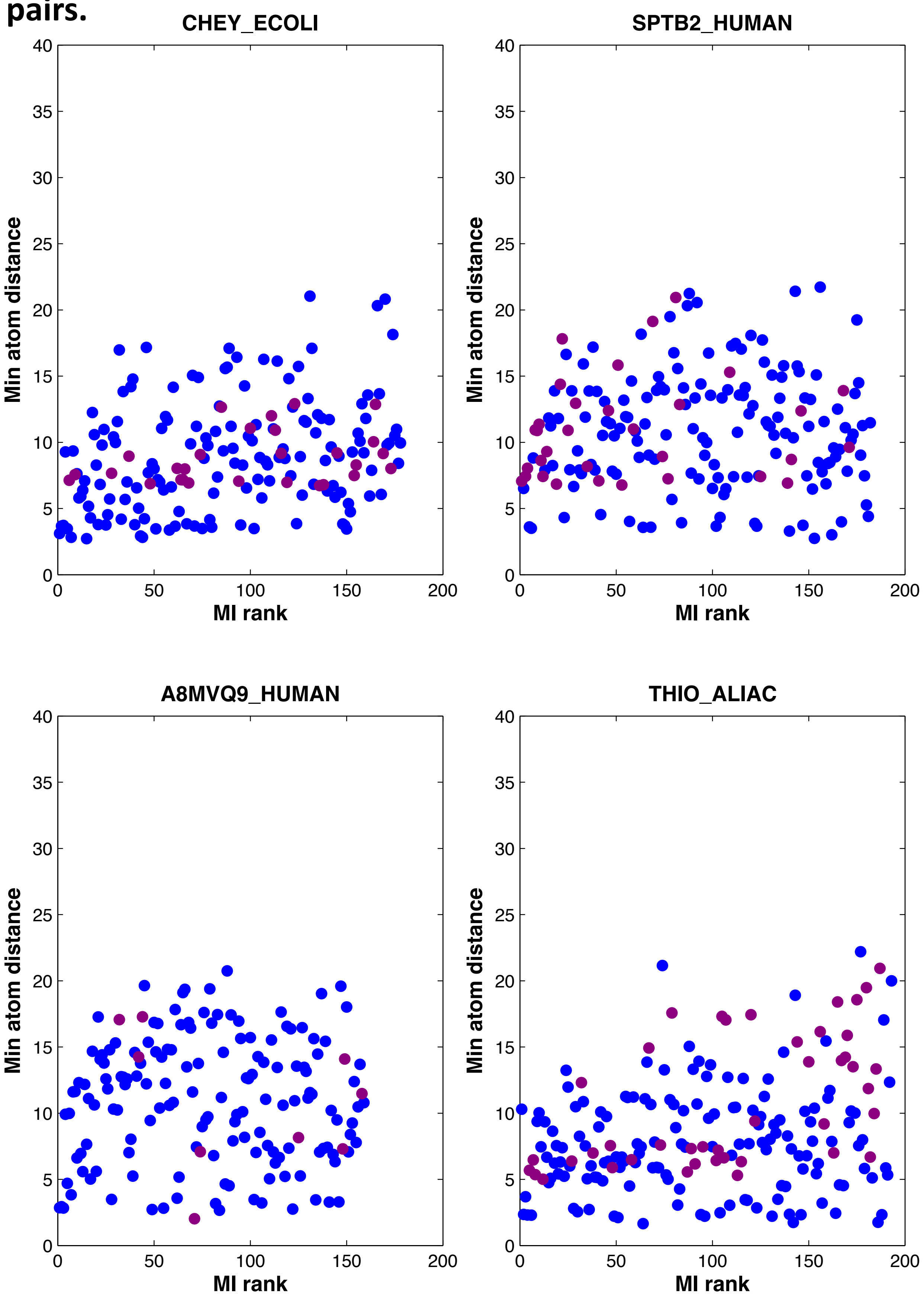

Figure S13. The minimum atom distance of top 200 ranked MI pairs.

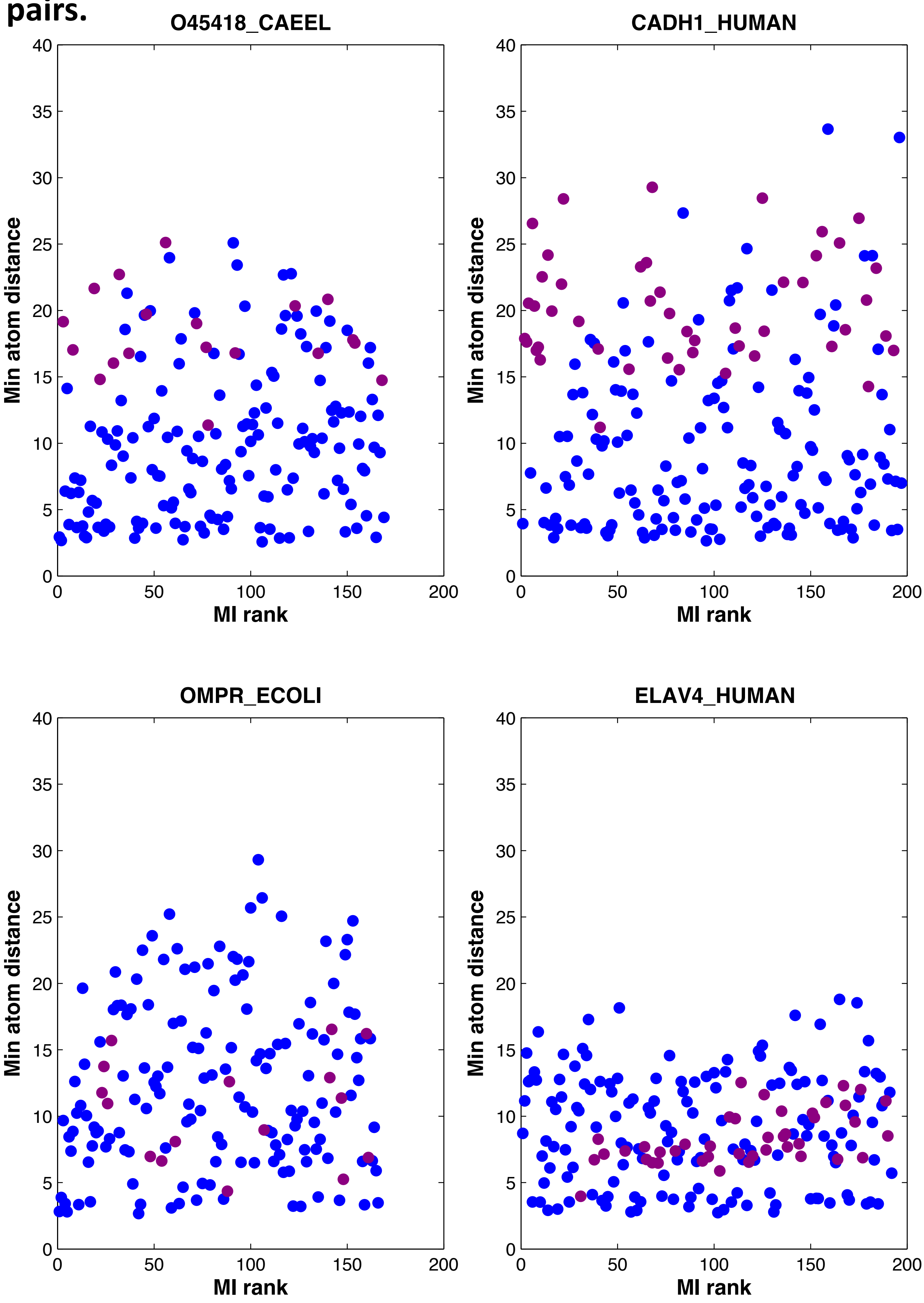

Figure S13. The minimum atom distance of top 200 ranked MI pairs.

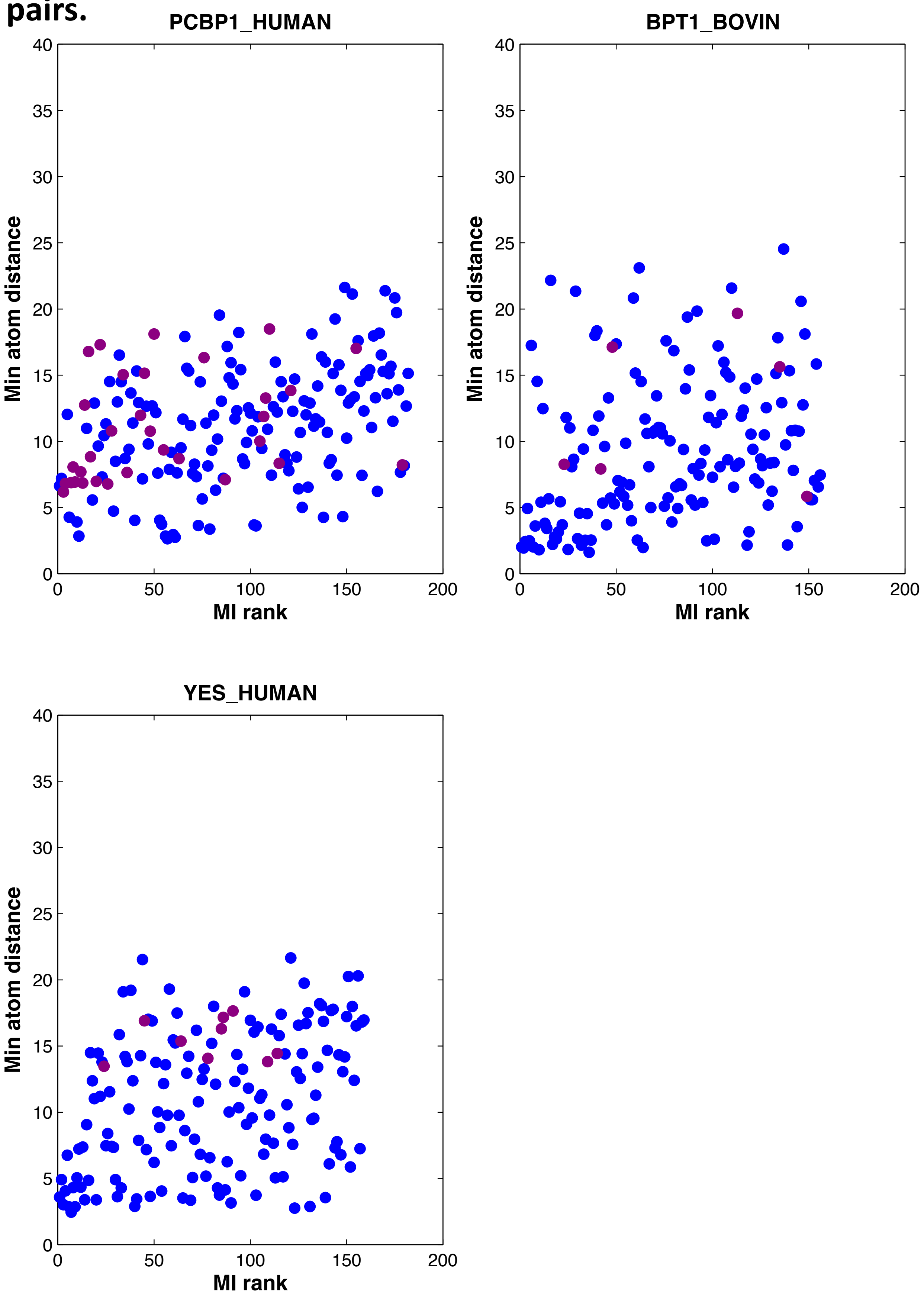

Supplement: Figure S13 — The minimum atom distance of top 200 ranked MI pairs. (4 pages). For each for the 15 proteins, plots show the minimum distance between each MI ranked residue pair. In purple the MIs which are filtered out by our algorithm, Text S1 and all scores are available in Web Appendix A8. (PDF) [file pone.0028766.s013.pdf]
