# Supplementary material for: Protein 3D Structure Computed from Evolutionary Sequence Variation
Source: PLoS One. 2011 Dec 7;6(12):e28766. doi: 10.1371/journal.pone.0028766 (PMC3233603; doi:10.1371/journal.pone.0028766)

Figure S15. The minimum atom distance of top 200 ranked SCA pairs.

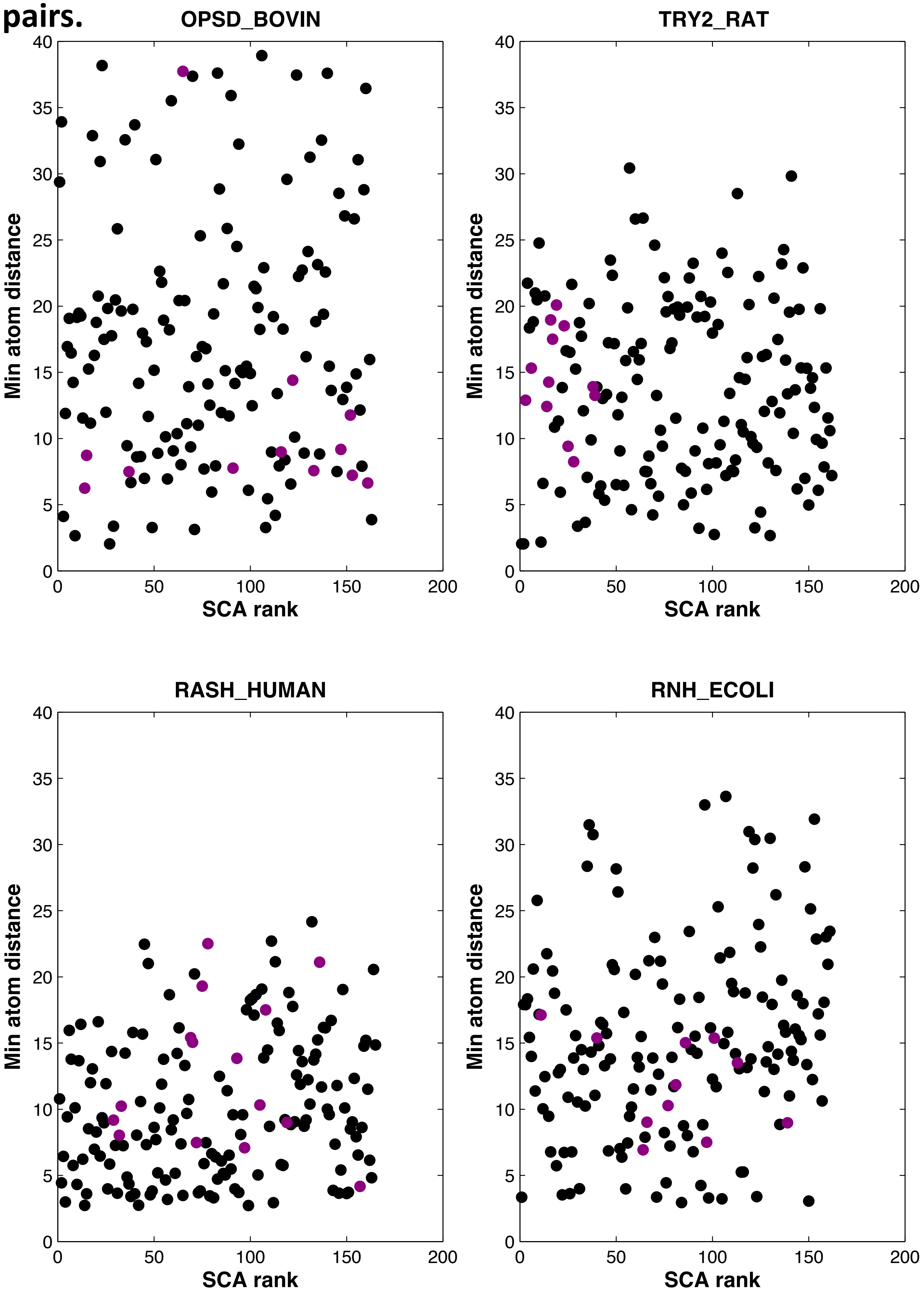

Figure S15. The minimum atom distance of top 200 ranked SCA pairs.

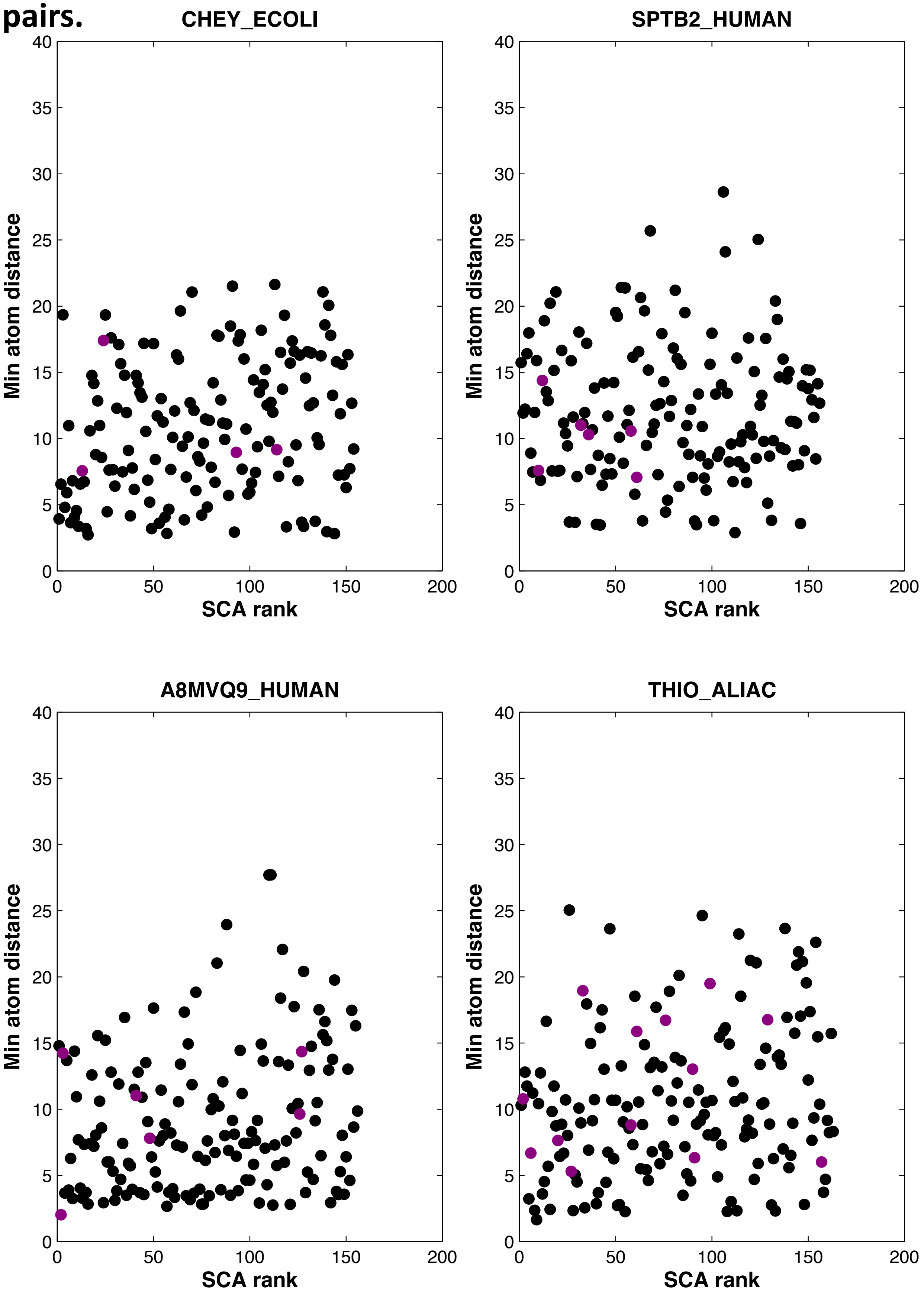

Figure S15. The minimum atom distance of top 200 ranked SCA pairs.

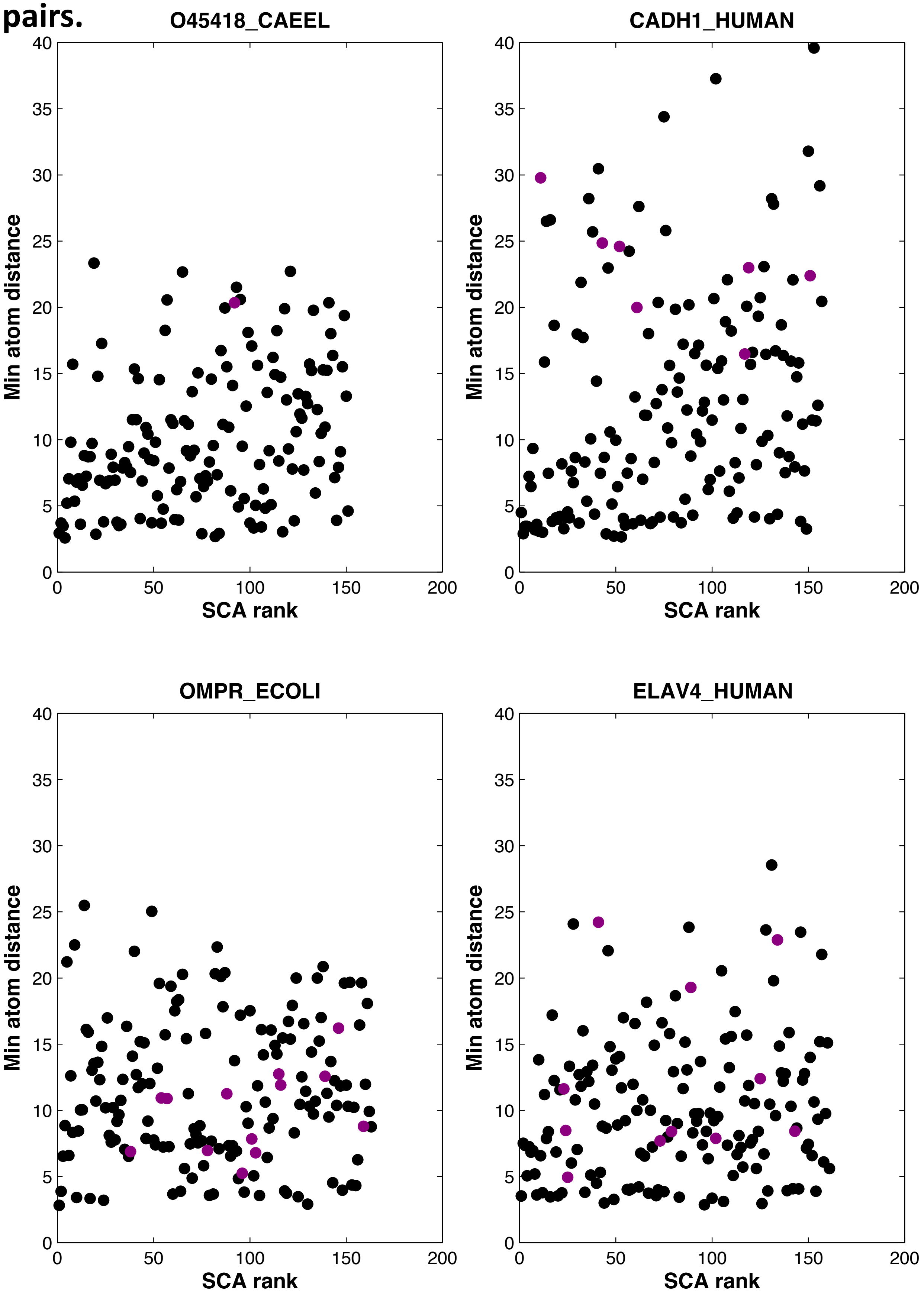

Figure S15. The minimum atom distance of top 200 ranked SCA pairs.

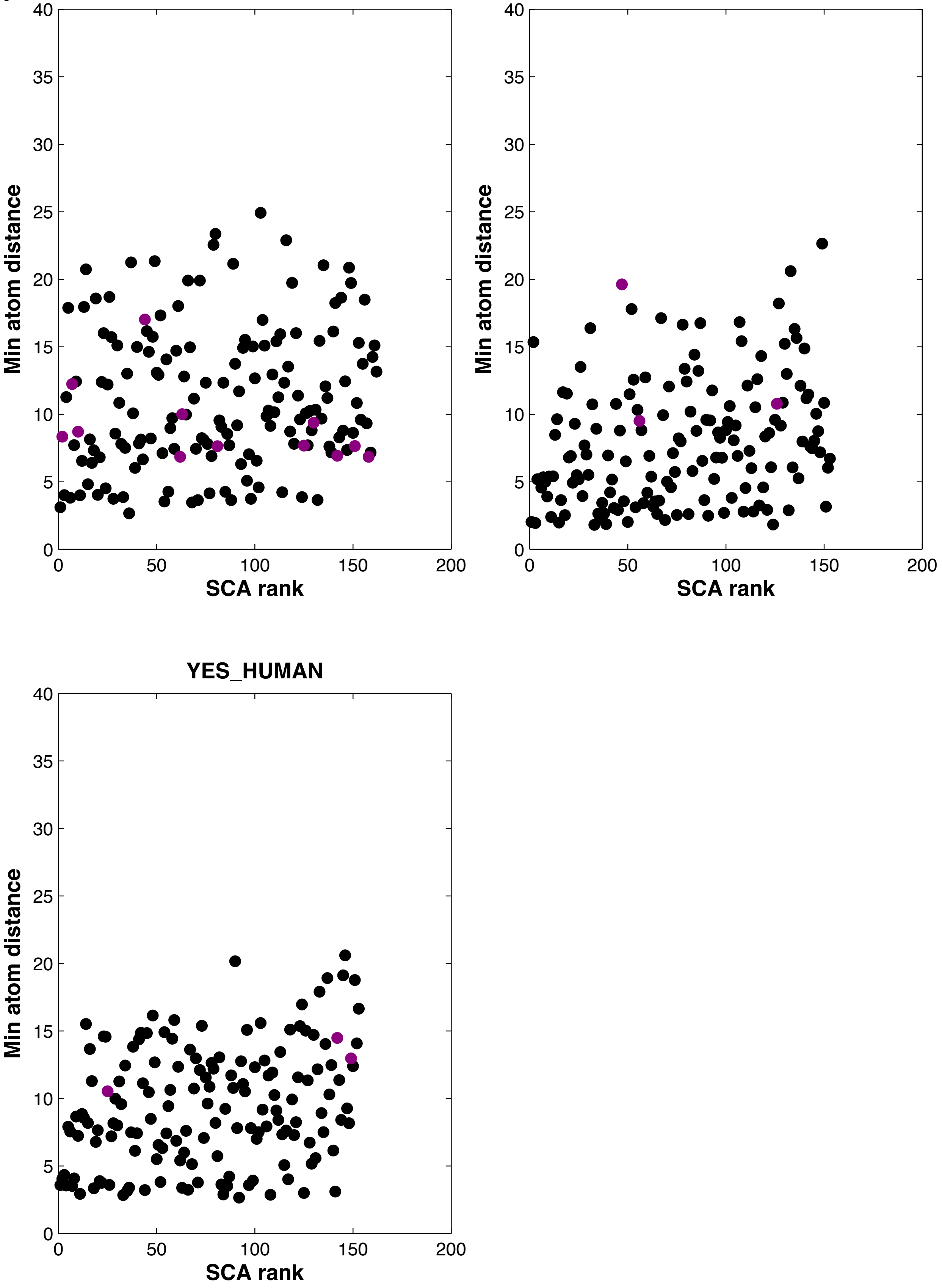

Supplement: Figure S15 — The minimum atom distance of top 200 ranked SCA pairs. (4 pages). For each for the 15 proteins, plots show the minimum distance between each DI ranked residue pair. In purple the SCAs which are filtered by our algorithm, Text S1 and all scores available in Web Appendix A10. (PDF) [file pone.0028766.s015.pdf]
