# Supplementary figures and images for: Protein 3D Structure Computed from Evolutionary Sequence Variation
Source: PLoS One. 2011 Dec 7;6(12):e28766. doi: 10.1371/journal.pone.0028766 (PMC3233603; doi:10.1371/journal.pone.0028766)

**Figure S16. Number of distance constraints required for best 3D structure prediction.**

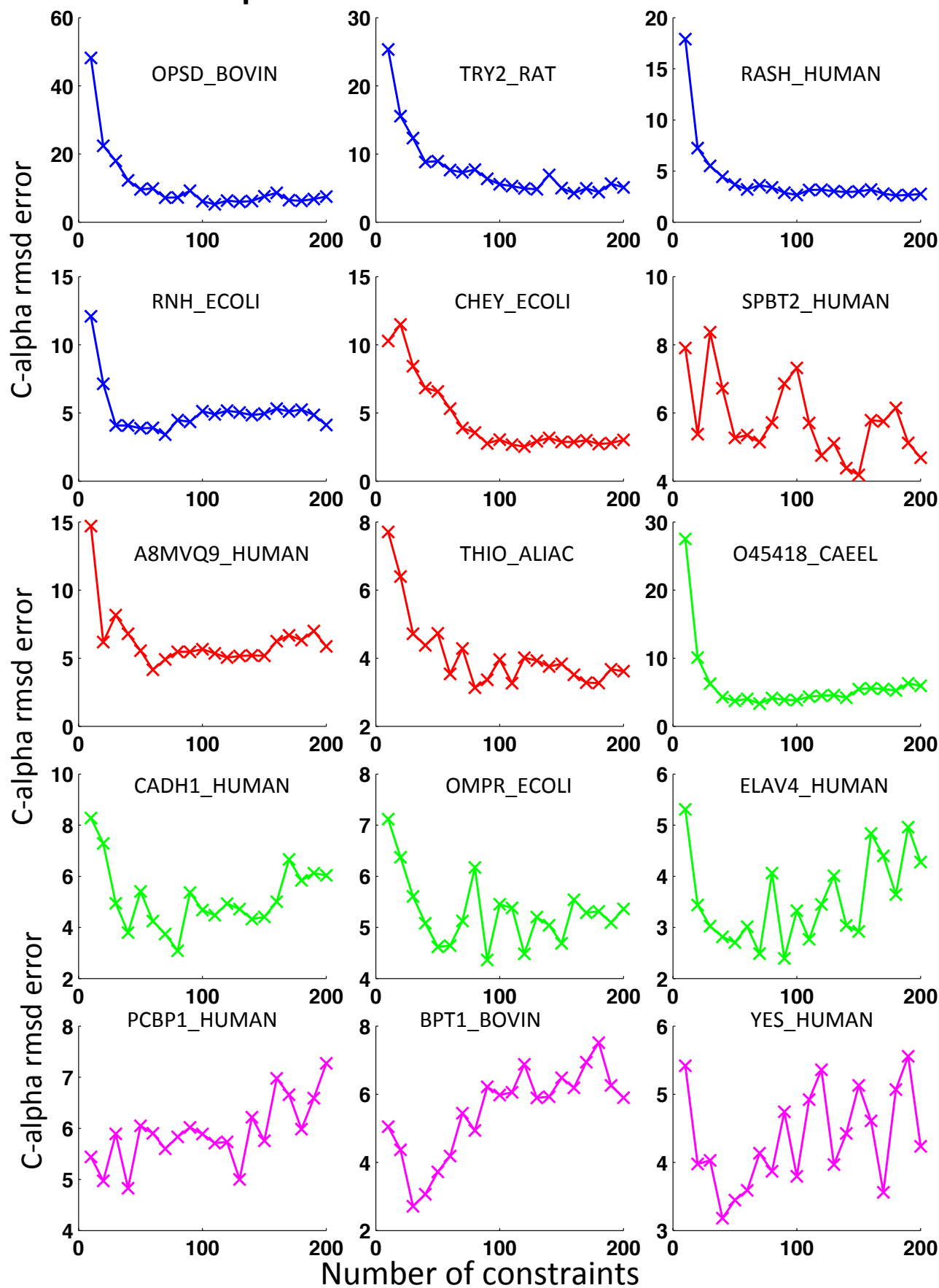

Supplement: Figure S16 — Number of distance constraints required for correct 3D structure prediction. With increasing number of predicted essential distance constraints (NC, horizontal axis), 3D prediction error decreases rapidly, as assessed by Cα-RMSD between the best of 20 (in each NC bin) predicted structures and the observed structure (here, for the 15 test proteins, using Pymol) shown separately. Remarkably, as few as ∼NRES/2 (∼L/2) distance constraints dij (with chain distance |i−j|>5) suffice for good quality predictions below 5 Å Cα-RMSD, where NRES is the number of amino acid residues in the protein multiple sequence alignment. (PDF) [file pone.0028766.s016.pdf]
