# Supplementary material for: Protein 3D Structure Computed from Evolutionary Sequence Variation
Source: PLoS One. 2011 Dec 7;6(12):e28766. doi: 10.1371/journal.pone.0028766 (PMC3233603; doi:10.1371/journal.pone.0028766)
Supplement: Text S1 — Supplementary Methods and Analysis. (PDF) [file pone.0028766.s017.pdf]

Protein 3D structure computed from evolutionary sequence variation

Debora S Marks*, Lucy J Colwell*, Rob Sheridan, Thomas A Hopf, Andrea Pagnani, Riccardo Zecchina, Chris Sander

* Joint first authors

Supplement and Appendices at: http://cbio.mskcc.org/foldingproteins

Supplementary Text and Figures

Methods for coupling predictions and folding proteins **2**
  Summary **2**
  Principle: From co-evolution to distance constraints **3**
  Direct coupling analysis (DCA) to infer 3D contacts (EICs) **3**
    Protein family alignments 3
    Multiple sequence alignments 4
    Weighting protein sequences 4
    Mutual information 5
  Maximum entropy sequence model **5**
    Mean field approximation 7
    Inferred residue pair couplings 8
  From Direct Information (DIs) to Inferred Contacts (EICs) **9**
    Use of Primary Sequence Position 9
    Conservation filter 9
    Cysteine pairs and disulfide bonds 9
    Secondary Structure prediction 10
  Folding the Proteins **10**
    The distance constraints from EICs 10
    Distance constraints from secondary structure 10
    Number and ranking of inferred contacts 10
    Distance geometry to generate trial structures 11
    Annealing 11
    Energy minimization 11
Assessment of contacts and folded structures **12**
  Ranking predicted structures **12**
    In summary 12
    α-helix and β-sheet twist angle criteria 12
    Blind detection of β sheets in predicted structures 13
    Folding without secondary structure 13
    Algorithm performance comparison 13
    Visualizing contacts in predicted folded structures 14
    Analysis of accuracy of inferred contacts in 2D contact space 14
    Quantitative measure of false positives 14
    Quantitative measure of contact prediction spread 15
    Control calculations folding proteins using observed residue contacts 15
    Mapping between PDB and PFAM 15

References 16

Methods for coupling predictions and folding proteins

Summary

The essential components of our method for the prediction of a 3D protein structure using evolutionary sequence information without the use of structural templates are:

(1) Protein sequence alignment for the protein family containing the target protein.
(2) Formulation of a global statistical model for sequences in a protein family.
(3) Derivation of parameters that maximize entropy in this model, using direct coupling analysis (DCA).
(4) Derivation of a ranked set of evolutionarily inferred contacts (EICs).
(5) Secondary structure prediction using well-established methods.
(6) Implementation of weighted distance restraints from inferred contacts.
(7) Application of distance geometry and constrained molecular dynamics.
(8) Automated ranking of predicted structures to nominate a single predicted structure and a set of lower-ranked alternatives.
(9) Evaluation, effectively blinded, of prediction accuracy.

The goal of our statistical analysis of co-variation in protein sequences is the inference of residue-residue proximity within an iso-structural protein family. While it is plausible that residues in close proximity tend to co-vary, the inverse is not necessarily true, i.e., residue correlations can and do occur between amino acids that are not physically close. For example, co-variation may result from spatially distant coupling via external interaction partners, such as oligomerization (homo- or hetero-), binding of biomolecular substrates such as RNA/DNA, or via transitivity (see main text). In addition, residues close in space do not necessarily co-vary. If inference were perfect, residue pairs with the highest correlation scores would be physically close in the folded structure. In reality, however, the inference of spatial proximity from residue pair correlations is susceptible to both false negative and false positives. The goal of the statistical method and inference rules developed is to optimize prediction accuracy of the resulting 3D structures.

Starting from the observed residue counts for each sequence position in a multiple sequence alignment, containing hundreds or thousands of members of a protein family, we quantify amino acid co-variation between each pair of sequence positions generating an initial set of correlation scores. We then use a maximum entropy approach, direct coupling analysis (DCA), to derive a set of essential pair couplings.
DCA aims to maximize the number of directly coupled pairs and to minimize the number of indirect couplings, i.e., residues connected via directly coupled pairs, we term this transitivity. The principal result is a minimal set of residue pairs whose coupling strengths (interactions) are sufficient to explain the complete set of observed amino acid co-variation values. From the set of DCA coupling scores (Eqn. 22) we derive and rank a set of evolutionary inferred contacts (EICs) – residue pairs predicted to be in physical proximity in the folded structure. This set of EICs is converted to distance restraints, which are used as input to distance geometry and simulated annealing calculations. These calculations start with the fully extended polypeptide chain of a single target protein of interest, chosen from the family, and results in a set of folded structures. Typically 2*L structures (120 for a 60-residue protein, 440 for a 220-residue protein) are generated, from which a single, top-ranked predicted structure can be selected.
Evidence suggests that accuracy of contacts is not sufficient to evaluate the ability of the contacts to predict a protein fold. Hence the real assay to test the 'accuracy for folding' is to predict the 3D structure from the contacts alone.

Principle: From co-evolution to distance constraints

How and why do patterns of amino acid co-evolution contain information about residue-residue contacts in 3D? Imagine a simple evolutionary scenario in which one or more residues of a protein sequence randomly mutate, affecting the fitness of the protein. Functional or structural constraints on this protein could require other residues to change in response to the first change to 'rescue' the functional phenotype of the protein in the context of the evolving organism. For example, in response to increasing the size of residue *i* in the protein interior, say from ALA to ILE, a neighboring residue *j* might need to reduce in size, say LEU to ALA, keeping the overall volume occupancy of the pair *i,j* approximately constant. Similarly a +/- charge pair could evolve to -/+ charge pair, maintaining a favorable interaction. Inspection of known 3D structures and homologous sets of protein sequences reveals many cases of physically interacting residues that co-evolve, perhaps the first published observation is by Bloomer et al. [1].

However, there are several other plausible causes of residue correlations. A particularly important one is transitivity, where primary correlations in two proximal residue pairs, say (*i,j*) and (*j,k*), lead to significant correlation between residues *i* and *k*, despite their lack of proximity [Box1 in main text, also termed 'indirect correlation']. In addition, residue pair correlations can be caused by physical contact between two monomers in a protein complex; or, other, more complicated constraining interactions, such as substrate binding. As a consequence, the inverse inference, from pair correlation to physical contact within one protein chain, will often be incorrect, generating false positives, as discussed in Main text. After computation of all pair correlations from a multiple sequence alignment we are therefore faced with the difficult statistical problem of ascertaining which pairs consist of residues in close physical proximity.

A simple estimate illustrates the numerical complexity of the problem. A protein family with, say, 100 aligned residues, has just under 5,000 different pairs (*i,j*). A globular protein of this size has approximately 600 physical residue-residue contacts, where a contact is defined as the two residue centers (C_a atoms) being within 8 Å of each other. So in this example, if the inference were perfect, we would expect the top ~ 10% of residue correlations to imply residue-residue contacts. Reconstruction from known contacts has shown that one needs in the order of 25-40% of real contacts, selected randomly, to reconstruct the protein fold [2,3,4], so a protein of ~100 residues may need as many as 50-150 correct constraints.

We therefore face the challenge of ranking residue-residue pair correlations computed from multiple sequence assignments such that the top N constraints accurately predict proximity in 3D space. To meet this challenge, we look for a minimal set of pair interactions that, through transitivity, will produce all the observed pair correlations. The maximum entropy expansion addresses this requirement by requiring a minimally constrained probability model that is consistent with the observed pair counts $f_{ij}(A,B)$. This principle has been used previously for other biological problems, such as genetic regulation and correlations between neuronal spikes [5,6,7] and here is applied to co-variation in residue positions, earlier termed correlated mutations [1,8,9,10,11]. The mean field approximation implements a particular functional form for the maximum entropy pair terms, using the notion of effective pair interactions for any pair (*i,j*) in an average single residue field at *i* and *j*, which reflect the influence of all other interactions [12].This leads to an efficient computational procedure for inferring a set of basic residue-residue interaction parameters.

Direct coupling analysis (DCA) to infer 3D contacts (EICs)

Protein family alignments

We chose a diverse set of protein families from the PFAM collection [13] for the purposes of testing and analyzing the predictive power of the DCA-EIC method using these criteria: (i) size of the protein family *M*, currently set at M≥1000 sequences per protein family; (ii) range of protein sizes *L*; (iii) inclusion of the main protein fold families, such as all-α, α/β, α+β and all-β; (iv) availability of experimentally derived (PDB) structures for at least one family member to allow blinded accuracy tests, Table S1. The PFAM

collection of multiple sequence alignments for more than 10,000 protein domain families has the advantage of being pre-computed, archived, regularly updated, easily accessible and widely used. Each PFAM alignment contains a large number of sequences and may reach low levels of sequence similarity (below 20% sequence identity in some families). Each PFAM family is assumed to be iso-structural, so that all protein structures in a family form a tight and distinct cluster in protein structure space [14], though this is a simplifying assumption which can be revisited in future work. We find that the 2011 PFAM collection provides a huge increase in evolutionary information since the time of earlier attempts to predict residue contacts from multiple sequence alignments [15] [9]., and is increasing exponentially.

Multiple sequence alignments

The multiple sequence alignment is organized as a $M \times L$ matrix $\left\{A_i^m\right\}$ of amino acid residues in proteins *m=1,M* (rows) at sequence positions *i=1,L* (columns). Each matrix element $A \in \{A,C,D,E,F,G,H,I,K,L,M,N,P,Q,R,S,T,V,W,Y,-\}$ can take *q=21* values, for the 20 amino acids and a sequence gap. The starting points of the algorithm are the single and pair residue frequencies

$$f_i\left(A\right) \equiv \frac{1}{M}\sum_{m=1}^{M}\delta\left(A_i^m, A\right) \qquad \text{Eq (1)}$$

$$f_{ij}\left(A,B\right) \equiv \frac{1}{M}\sum_{m=1}^{M}\delta\left(A_i^m, A\right)\delta\left(A_j^m, B\right) \qquad \text{Eq (2)}$$

at sequence positions $1 \le i, j \le L$, and where *A* is a variable for the amino acid residue type at position *i*, and *B* a variable for the amino acid residue type at position *j*. Counting is formalized using a delta function $\delta\left(a,b\right)$, equals 1 when $a = b$ and zero otherwise. If columns *i* and *j* were statistically independent, the joint empirical frequency distribution $f_{ij}(A,B)$ would be approximately equal to the product of the individual frequency distributions $f_i(A) \cdot f_j(B)$. In general, departure from equality measures the statistical correlations between sequence positions.

Weighting protein sequences

We wish to optimize the detection of correlations in multiple sequence alignments, which arise due to evolutionary constraints. However, spurious correlations may also arise for reasons independent of maintaining protein structure and function such as: (i) phylogenetic correlations from residue pairs that appear correlated after species divergence because of a low mutation rate; (ii) uneven sampling in the space of natural sequences, due to experimental ascertainment bias in sequencing projects as a result of sequencing many closely related species; and, (iii) 'transitive' correlations that arise via direct correlations.

To reduce the influence of spurious correlations that arise due to sampling bias in the sequence alignment, we assign a lower weight to highly similar sequences and a higher weight to sequences dissimilar to other family members. Several weighting schemes are available in the literature, e.g. [16]. Here we take a straightforward approach, if *L* is the sequence length, we set a similarity threshold *x,* where $0 \le x \le 1$, and group together sequences with more than *xL* identical residues. More precisely, for each sequence *m* in the alignment we compute the number of sequences k_m whose similarity to sequence *m*, quantified as the total number of aligned identical residues relative to *m*, is larger than *xL*; formally,

$$k_m \equiv \sum_{n=1}^{M} \theta\left(\sum_{i=1}^{L} \delta\left(A_i^m, A_i^n\right) - xL \right) \qquad \text{Eq (3)}$$

where θ is the unit step function. We then redefine frequency counts in equations (1) and (2) by down-weighting each sequence *m* by the inverse neighborhood density $1/k_m$ in sequence space, as:

$$f_i(A) \equiv \frac{1}{\lambda + M_{\text{eff}}} \left(\frac{\lambda}{q} + \sum_{m=1}^{M} \frac{1}{k_m} \delta\left(A_i^m, A\right) \right) \qquad \text{Eq (4)}$$

$$f_{ij}(A,B) \equiv \frac{1}{\lambda + M_{\text{eff}}} \left(\frac{\lambda}{q^2} + \sum_{m=1}^{M} \frac{1}{k_m} \delta\left(A_i^m, A\right) \delta\left(A_j^m, B\right) \right) \qquad \text{Eq (5)}$$

where $M_{eff} = \sum_{m=1}^{M} \frac{1}{k_m}$ is the effective number of sequences in the MSA after reweighting at the relevant position(s). The lambda term (pseudo-count) is used to regularize the data for finite data sets as described in [9]. Numerical tests on a variety of different MSAs led to the choice of x ~ 0.7, and λ ~ 0.5. Setting x ~ 0.7 translates to treating all sequences with more than 30% identical residues to a sequence *m* as having unit weight as a group.

Mutual information

A popular measure of correlation among pairs of randomly distributed variables is the mutual information (MI) which is defined in terms of the empirical frequency distributions in equations 4 & 5 as:

$$MI_{ij} = \sum_{A,B} f_{ij}(A,B) \ln\left(\frac{f_{ij}(A,B)}{f_i(A) f_j(B)} \right) \qquad \text{Eq (6)}$$

The mutual information, or information gain, M_{ij} between residue positions i and j is a relative entropy is equal to the divergence (Kullback-Leibler) $D_{KL}(f_{ij} \,||\, f_i f_j)$, between the co-occurrence probability distribution f_{ij} and the factorized model distribution $f_i f_j$.

Maximum entropy sequence model

Several methods have been used to perform unsupervised inference of residue-residue contacts from multiple sequence alignments (MSAs), ranging from purely local statistical analysis of correlations [9,17,18,19,20,21] to more global approaches that use Bayesian and maximum entropy techniques [22,23,24]. Here, 'local' means that positions *i* and *j* are considered independently, while 'gobal' means that the score for the pair *i* and *j* depends on the rest of the alignment. Recent work suggests that the latter methods are better at inferring residue-residue contacts from MSAs than local statistical methods [24]. Our maximum entropy model aims to identify a minimal set of coupled pairs, as the result of a global inference calculation, from which we infer residue proximity. In this respect it is conceptually different

from approaches that calculate the correlation of each pair of residues independently, without global considerations (*e.g.* methods based on the MI defined in equation 6). The local methods, reviewed in [20], are intrinsically unable to distinguish between 'causal' (direct) correlations and transitive correlations.

The statistical model for each protein family describes the probability of occurrence of the amino acid sequence of any particular family member as a joint probability distribution $P(A_1,\ldots,A_L)$. In general the estimate of such function is an intractable task, as the number of parameters specifying this joint probability distribution is q^L. The problem simplifies considerably if one limits the objective to estimating a probability distribution which describes the single and pair residue frequencies observed in the MSA. We thus define

$$P_i(A_i) \equiv \sum_{\{A_k=1,\ldots,q\}|k\neq i} P(A_{1,\ldots,}A_L) = f_i(A_i) \qquad \text{Eq (7)}$$

$$P_{ij}(A_i,A_j) \equiv \sum_{\{A_k=1\ldots q\}|k\neq i,j} P(A_i\ldots A_L) = f_{ij}(A_i,A_j) \qquad \text{Eq (8)}$$

where the sum is over all possible sequences, i.e, over all possible amino acid values A_k at each position k, except for those constrained by the left hand side of the equation. In principle one could also consider triplet terms, i.e., correlations between 3 residues or, in general, k-residues correlations with k≥2. However, even for k=3, the number of parameters to be inferred would grow enormously, scaling like $\sim \binom{L}{3} q^3$, where L is the sequence length.

There is a large number of probability distributions, which are consistent with these data. We choose the maximally flat distribution (consistent with the empirical data constraints), that is the model with **maximum entropy** S, where:

$$S = -\sum_{\{A_i|i=1,\ldots,L\}} P(A_1,\ldots,A_L)\ln P(A_1,\ldots,A_L) \qquad \text{Eq (9)}$$

The solution to this maximization problem is standard and explained in textbooks [25]. Lagrange multipliers $h_i(A_i)$ and $e_{ij}(A_i, A_j)$ are introduced allowing enforcement of the crucial compatibility condition with the empirical one- and two-residues frequency distributions, i.e., with the empirical data. The joint probability distribution can be written as:

$$P(A_1,\ldots,A_L) = \frac{1}{Z}\exp\left\{\sum_{1\leq i<j\leq L} e_{ij}(A_i,A_j) + \sum_{1\leq i\leq L} h_i(A_i)\right\} \qquad \text{Eq (10)}$$

$$Z = \sum_{\{A_i|i=1,\ldots,L\}} \exp\left\{\sum_{1\leq i<j\leq L} e_{ij}(A_i,A_j) + \sum_{1\leq i\leq L} h_i(A_i)\right\} \qquad \text{Eq (11)}$$

where the choice of the parameters e_{ij}, h_i is such that the constraints in equations 7 & 8 are satisfied and Z is the normalization constant which depends only on the model parameters. The main challenge in satisfying the constraints in equations 7 & 8 is to efficiently compute from equation 10 the one- and two-residue marginals ($P_i(A)$ and $P_{ij}(A,B)$), and the partition function Z. Formally, the marginals of this distribution are given by:

$$\frac{\partial \ln Z}{\partial h_i(A_i)} = -P_i(A_i) \quad \text{Eq (12)}$$

$$\frac{\partial^2 \ln Z}{\partial h_i(A_i)\partial h_j(A_j)} = -P_{ij}(A_i,A_j) + P_i(A_i)P_j(A_j) \quad \text{Eq (13)}$$

However, the direct computation of equations 12 & 13 is computationally prohibitive. Different strategies have been implemented to address this problem: in [23] the Bethe approximation strategy, originally proposed in [26,27] was chosen. In addition researchers have tried Monte Carlo sampling [6], and perturbative schemes [28,29].

As discussed in [9] the model has a scaling ambiguity, a so called 'gauge invariance' that, without loss of generality, can be addressed by the following relations:

$$e_{ij}(A,q) = e_{ij}(q,A) = h_i(q) = 0 \quad \text{Eq (14)}$$

for the sequence positions $1 \le i < j \le L$, and amino acid types $A=\{1...q\}$.

Mean field approximation

Our approach considers a Taylor expansion of the free energy, proposed by Plefka [30], and subsequently by Georges and Yedidia [31] in the context of the Ising spin-glass model (which in this context corresponds to the q = 2 case). The Plefka expansion to first order provides what is known in physics as the mean-field approximation, where the couplings are assumed to be zero. While there exist different techniques for deriving the same approximation, the Plefka approach is particularly elegant and simple. We introduce a small parameter α into equation 11:

$$Z(\alpha) = \sum_{\{A_i | i=1,...,L\}} \exp\left\{ \alpha \sum_{1\le i<j\le L} e_{ij}(A_i,A_j) + \sum_{1\le i\le L} h_i(A_i) \right\} \quad \text{Eq (15)}$$

Now consider the Legendre transform of $-\ln Z$:

$$G(\alpha) = \ln Z(\alpha) - \sum_{\{i=1,...,L\}} \sum_{A_i=1,...,(q-1)} h_i(A_i)P_i(A_i) \quad \text{Eq (16)}$$

We are now interested in approximating $G(\alpha)$ to first order in α using a Taylor series expansion:

$$G(\alpha) \approx G(0) + \left.\frac{\partial G(\alpha)}{\partial \alpha}\right|_{\alpha=0} \alpha \qquad \text{Eq (17)}$$

In this approximation, considering the gauge invariance in equation 14, one obtains the key result, for $i \neq j$ and $1 \leq A_i, A_j \leq q-1$:

$$\left(C^{-1}\right)_{ij}(A_i, A_j)\Big|_{\alpha=0} = -\mathrm{e}_{ij}(A_i, A_j) \qquad \text{Eq (18a)}$$

and on the diagonal, for $i=j$ and, again, for $1 \leq A_i, A_j \leq q-1$:

$$\left(C^{-1}\right)_{ij}(A_i, A_j)\Big|_{\alpha=0} = \frac{\delta_{A_i, A_j}}{P_i(A_i)} + \frac{1}{P_i(q)} \qquad \text{Eq (18b)}$$

Where we defined $C_{ij}(A_i, A_j) = f_{ij}(A_i, A_j) - f_i(A_i) f_j(A_j)$. It is of technical interest that without the restriction to q-1 amino acids the correlation matrix C would not be invertible.

In practice the computation of the inferred couplings involves the following steps:

(i) compute the observed residue counts in single columns and pairs of colums, f_i and f_{ij}, from the multiple sequence alignment (equations 4 & 5);

(ii) build the empirical correlation matrix $C_{ij}(A_i, A_j) = f_{ij}(A_i, A_j) - f_i(A_i) f_j(A_j)$

(iii) invert this matrix to obtain the couplings e_{ij}.

Inferred residue pair couplings

The estimation of the e_{ij} couplings allows us to rank the coupling strength of the residue pairs through the 'direct information' (DI) [9]. For any pair of residues i, j we introduce an effective two residue model:

$$P_{ij}^{Dir}\left(A_i, A_j\right) = \frac{1}{Z} \exp\left\{e_{ij}\left(A_i, A_j\right) + \tilde{h}_i\left(A_i\right) + \tilde{h}_j\left(A_j\right)\right\} \qquad \text{Eq (19)}$$

The new fields $\tilde{h}_i, \tilde{h}_j$ can be computed imposing the single residue marginal frequency count compatibility condition:

$$\sum_{A_j=1}^{q} P_{ij}^{Dir}\left(A_i, A_j\right) \cong f_i\left(A_i\right) \qquad \text{Eq (20)}$$

$$\sum_{A_i=1}^{q} P_{ij}^{Dir}\left(A_i,A_j\right) \cong f_j\left(A_j\right) \qquad \text{Eq (21)}$$

and the term Z_{ij} by normalization. Finally, we define the direct information between residues *i* and *j*, DI_{ij}, as the relative entropy between the distributions P_{ij}^{Dir} and the independent site distribution $f_i f_j$:

$$DI_{ij} = \sum_{A_i,A_j=1}^{q} P_{ij}^{Dir}\left(A_i,A_j\right) \ln \frac{P_{ij}^{Dir}\left(A_i,A_j\right)}{f_i\left(A_i\right) f_j\left(A_j\right)} \qquad \text{Eq (22)}$$

The DI_{ij}, ranked by their numerical values, describe the evolutionary couplings inferred for the alignment of interest [12] and are the basis for inferring evolutionarily maintained contacts EIC.

From Direct Information (DIs) to Inferred Contacts (EICs)

The algorithm described above reduces the set of empirically correlated residue pairs, to the minimal set of pairs most likely to co-vary due to evolutionary constraints. The full set of resulting pair correlation scores for the set of proteins in Table S1 is available at http://cbio.mskcc.org/foldingproteins (Appendix A1). This section details the information derived from the protein sequence, which is used to remove high scoring pairs that are unlikely to be close in the folded structure. The resulting set of EIC pairs contain information about which residues are most likely to be in close proximity of each other (Appendix A1). This information is subsequently used to reduce the space of possible 3D conformations that this protein can assume.

Use of Primary Sequence Position

Sequence neighbors are likely to co-vary due to the nature of the polypeptide chain. Empirically we observe that residue pairs separated by four or five positions in sequence often have high DI scores without being in close physical proximity in the folded protein. We therefore set the DI scores of all pairs separated by five or fewer positions in sequence to zero. The DCA algorithm makes no use of connectivity in the polypeptide chain, but could be adapted to do so in future algorithmic developments.

Conservation filter

Conserved residues, on average, are more likely to be located in the interior of a globular protein and to have more spatial neighbors than residues on the surface [32,33]. So one might hope to be able to use conserved residues for contact prediction [20]. However, *completely* conserved residues produce no correlation signal whatsoever, as they do not vary. *Nearly* conserved residues *may* produce a correlation signal but this is subject to statistical uncertainty, because of the small number of varying positions [20]. To deal with this uncertainty, we do not use correlations involving residue positions *i* or *j* that are highly conserved, i.e., where more than 95% of sequences have the dominant residue at position *i* or *j*. We make an exception for cysteine residues which are more than 95% conserved by allowing a single distance constraint to one other cysteine residue. (Excluded constraints are marked in the DIscores file with '888' in column 8, Web Appendix A1)

Cysteine pairs and disulfide bonds

As each Cys side chain is only able to form a disulfide bond with one other Cys side chain, we allowed each cysteine residue to be paired with at most one other cysteine residue. Thus for each cysteine residue we allowed its highest ranking cystein-cystein pairing, and ignored other pairs involving this residue in

the ranked DI pair list. For example, in trypsin inhibitor, for Cys 55, we allow the highest ranking cysteine pair interaction for Cys 55, which pairs Cys 55 with Cys 5, but do not use any of the lower ranked cystein pairs, such as Cys 55 with Cys 30. Such pairs are marked with 222 in column 9 of the relevant the DIScores.txt file (Web Appendix A1).

Secondary Structure prediction

We use two algorithms, PredictProtein and PsiPred, to calculate the secondary structure assignments for each amino acid from the primary sequence [34,35]. The residue assignments from these predictions are in Web Appendix A9 at http://cbio.mskcc.org/foldingproteins. Predicted secondary structures are used to derive local distance constraints between residues in these structures. We empirically observe that two residues in a secondary structure segment may coevolve without being close in structure, e.g. residues at opposite sides of a helix. Therefore, potential conflicts between predicted secondary structure and predicted EIC constraints are resolved by given precedence to the secondary structure prediction (details in Table S2). These rules were always applied irrespective of whether the predicted secondary structure was (in retrospect) correct, consistent with a blind prediction approach; clashes are marked as 999 in column 7 of the DIScores.txt files, Web Appendix A1

We anticipate that analogs of the four empirical rules involving primary sequence position, near-perfect conservation, Cys pairs and secondary structures can be incorporated in a more comprehensive theory in a future version of the DCA/EIC method.

Folding the Proteins

The distance constraints from EICs

Our prediction is that two residues i and j of a high-scoring EIC pair are in close spatial proximity in the protein structure. To generate all-atom 3D structures we constrained the space of possible 3D structures by requiring that the distance between the Cα atoms of i and j is less than 7Å, set as a harmonic constraint at 4 Å, (distance constraint files are in Appendix 3, http://cbio.mskcc.org/foldingproteins). A similar constraint was set for the Cβ atoms of pairs that did not contain a glycine. The assumption is that amino acids co-vary because they are in close physical proximity suggests the side chain atoms are proximal to each other in the structure. As the shape and size of side chains varies considerably among amino acids, therefore we also applied residue specific constraints to particular atoms for different amino acid pairs. Details of which atoms are provided in full in the CNS input files, see Web Appendix A10.

Distance constraints from secondary structure

Predicted secondary structure elements were used to supplement the EIC distance constraints, building on the high accuracy of secondary structure prediction methods [34,35]. We surveyed the distances between specific atom pairs within α-helices and β-strands in a set of protein structures. The means and standard deviations found in this survey were used to set distance constraints for pairs of residues predicted to lie in the same secondary structure unit, Table S2 and Web Appendix A9 http://cbio.mskcc.org/foldingproteins. These constraints were always applied irrespective of whether the predicted secondary structure was (in retrospect) correct, consistent with a blind prediction approach.

Number and ranking of inferred contacts

The number of constraints (EIC pairs, N_C) sufficient to successfully fold up a protein is of considerable theoretical and practical interest. The number may depend on many aspects, such as the distribution of EIC scores, the domain size, and the type of representative fold. In our unbiased and blinded approach structures are generated using a range of N_C values for each protein, and subsequently ranked using fully automatic quality metrics. In particular, for each protein we generate candidate structures using values of N_C that is up to 100% of the sequence length We calculated 20 structures for N_C = 30 up to L in steps of 10

where L is the length of the protein in the PFAM alignment. For instance, for protein of length 100 , we calculated 160 structures (20 structures per bin size). This range is comparable to the number of *true* distances needed as constraints to reconstruct a known protein structure, which is between 15-30% of the number of residues in the protein [2,3] as discussed above. We extended the analysis to calculate structures for very few constraints (<=20) and for larger numbers of constraints to test whether with hindsight a better number produced more accurate structures. We find that the computation of accurate structures is robust to a wide range numbers of constraints (see the graph of C_α-rmsd error of resulting candidate structures against the number of EIC pairs used as distance constraints, Figure 7 and Figure S16). While the computation of a larger number of candidate structures, is of interest in studying the robustness of the folding protocol, a single effectively blind prediction is always provided as the one structure with the highest rank.

Distance geometry to generate trial structures

Historically, distance geometry methods have been used in experimental structure determination by nuclear magnetic resonance spectroscopy (NMR) to generate trial structures. The approach is based on the premise that any three-dimensional structure can be defined as a set of inter-atomic distances. Conversely, a set of inter-atomic distances can be 'embedded' into three-dimensional space, (though not necessarily uniquely) to give the atomic coordinates of the protein. In NMR, to account for uncertainty of experimental distance constraints two distance matrices are generated, a matrix of lower bounds and a matrix of upper bounds. These matrices are then interpolated, or smoothed, so that the distances are consistent with each other. After 'smoothing' of the upper and lower bound matrices, a distance matrix that gives rise to a single trial structure is generated by selecting a random distance that lies between the upper and lower restraints for each residue pair.

We used the implementation of the Havel and Crippen distance geometry algorithm [36] in the NMR section of the cns_solve.1.21 suite of programs [37]. This distance geometry algorithm uses the distance constraints to 'embed' the extended starting structure within the ranges set by the constraints. We set the initial embedding algorithm to produce 20 trial structures The same parameter settings were used for all proteins evaluated (details of all parameter values used are in the 'dg_sa.inp' file at http://cbio.mskcc.org/foldingproteins, Web Appendix A2).

Annealing

Simulated annealing with standard protocols is used to regularize and refine the structures given by the starting coordinates generated by the distance geometry procedure. We observed empirically that the distribution of EIC scores for each protein has a long tail, data not shown. This suggests that the top scoring EIC pairs are more likely to represent true evolutionary constraints. To reflect this observation, we weighted the constraints for the simulated annealing part of the protocol using a function that emphasized the highest ranked EICs with a simple function, $10/i$, where i is the rank of the predicted contact, Web Appendix A11). The protocol begins with a starting temperature of 2000K and slowly increases the van der Waals scale factor (K_{vdw}) from 0.003 to 4.0 over 20 cycles of molecular dynamics (1000 steps). The temperature is lowered in steps of 25K until it reaches 300K. The high temperature scale factor for dihedrals is 5. This scale factor gradually increases to 200 during the slow cooling stage. The restraints are divided into two classes, those derived from the EICs and those derived from predicted secondary structure. The relative weights of the contributions to the potential energy shift during the annealing process, for example the dihedral constraints are up weighted relative to the EIC and secondary structure distance constraints, hence the influence of these distance constraints decreases during the structure refinement process.

Energy minimization

Energy minimization is performed in two stages after the simulated annealing protocol using the CNS default force field: 10 cycles of 200 steps of Powell minimization. The two classes of distance restraints are weighted as for the annealing steps. A further minimization protocol is then applied once hydrogen

atoms have been added to the candidate structures without the distance restraints; all scripts for CNS protocols are available in Web Appendix A1, and all final candidate structures in Appendix A3.

Assessment of contacts and folded structures

Ranking predicted structures

In summary

In the blinded prediction tests reported here, we construct a small set, from 40-480 of candidate 3D models for each protein. Candidate structures are generated using between 30 and L constraints, in increments of ten constraints, where L is the length of the domain. Structures are ranked using the quality of virtual torsion angles along predicted α-helices, and between predicted β-strands.

α-helix and β-sheet twist angle criteria

Distance geometry methods generate candidate structures that satisfy all distance constraints yet are topological mirror images of the correct structures [38]. These mirror images occur at different scales of organization of the protein and arise when constraints are sparse, as in this study. All-by-all 3D alignment of the set of candidate structures reveals clear clusters of predicted structures, one of which will contain the most accurate structures. Within mirror-inverted substructures, we observed secondary structure elements and pairs of secondary structure elements, notably β strand pairs, with the opposite chirality to that usually observed in proteins. This suggests that simple topological rules, based on the chirality of secondary structure units and pairs of units, can discriminate structures with mirrored sections versus those without, and can be used for objective ranking within a set of predicted structures.

To quantify such topological differences, we developed a simple method that measures the chirality of α helices and the twist between β strand pairs predicted to be adjacent in β sheets [39] and combine thes together in a weighted score which reflects the relative composition of predicted α and β elements in the protein. Firstly, we measure the handedness of predicted α-helices using the virtual dihedral angle $k(\alpha)(i)$ defined by four consecutive C_α-atoms at position i, i+1, i+2 and i+3. Right-handed helices have a range around $k(\alpha) \sim +1.5$ radians, while left handed helices ~ -1.5 radians. The quality of the helices is quantified and scored with a decreasing function around the idealized α-helix, see Appendix AX for precise values.

Secondly, we compute a virtual 'twist angle' between pairs of β strands. To calculate this we developed an algorithm which first detects the most likely pairing of β strands. For each residue *i* predicted to lie in a β strand, our algorithm finds the nearest other predicted β strand, if any, with a residue *j* that has its C_α-atom within 7 Å of the C_α-atom of residue *i.* The virtual dihedral angle $k(\beta)(i,j)$ between the four Cα-atoms i, i+2 and j, j-2 (for anti-parallel strand pairs) or j, j+2 for parallel pairs of β strands defines the strand-strand twist. Directionality of strands is computed as follows: if the distance between *(i+2)* and *(j+2)* is larger than the distance between *(i+2)* and *(j-2),* then the strands are anti-parallel, otherwise they are parallel (code available on request). Good structures tend to have negative values of $k(\beta)$, corresponding to a right handed twist of the strand pair when viewed along the strand direction, while mirror inversions tend to have positive values [40]. The algorithm calculates the proportion of β twist dihedrals which lie within an acceptable range with a deceasing function around an idealized twist dihedral.

Finally these α and β twist dihedrals are combined in a score weighted by the proportion of predicted α-helical residues and potential β twist dihedrals in the protein, all values for α and β virtual torsion and combined scores are available in Web Appendix A5. Since this is a blind prediction and we do not know the number of β twist dihedrals in an observed crystal structure, we estimate this based on the maximum number of β twist dihedral, which can be measured in any of the predicted structures. the top scoring candidate is then nominated as our top ranked structure as in Table S1, and all scores are available in

Web Appendix A5. Plots for each protein show visually the accuracy of this measure when compared to Ca-atom error (Figures S5).

The weighted scores derived from the α and β twist values, was found to correlate with the C_α-rmsd prediction error for 13 of the 15 proteins in the present study. The all helical and smaller proteins did poorly, Figure S5. Since α-helical handedness is a local geometric measure, it's not surprising that the helical score alone is insufficient to robustly rank predicted structures. This is in contrast to the β twist score for putative paired β strands, which is a less local three dimensional criteria and correlates more robustly with structure accuracy. This approach can probably be further developed, also using the handedness of β-strand cross-over connections, which are predominantly right-handed [41] as well as chiral relationships in helical bundles. We anticipate improvements of ranking criteria will include energy criteria, and quantitative assessments of constraint violations.

Blind detection of β sheets in predicted structures

To test the potential identification of β sheets for further refinement of our predicted structures, we developed an algorithm which calculates the most probable strand pairing and registration for predicted β strands in our predicted structures. As proof of principle, we applied this protocol to three test case protein families which contain β strands in very different topologies, PF00071 (Ras), PF00028 (Cadherin) and PF00076 (Elav4). The blind prediction of β sheets was conducted on the top ranked predicted structures for those families, structure numbers PF00071_P01112_130_17.pdb, PF00028_P12830_70_4.pdb and PF00076_P26378_40_12.pdb, (Table S1). Step (1) uses a combination of geometric criteria to identify an initial set of candidate strand pairs and their orientation., similar to the identification of nearest strands in the β twist algorithm used for ranking) These candidate pairs of β strands are then pruned to remove false positive associations and conflicting pairings, using similar criteria as in the β twist algorithm used for discrimination. Step (2) uses β-strand interaction potentials [1] and the Pfam multiple sequence alignment to score alternative possible registrations and hydrogen bonding patterns for each strand pair. Global optimization over these scores yields a consistent registration and hydrogen bond patterning of all connected strand pairs in a sheet. In Ras and cadherin, the identified strand pairs, their registration and hydrogen bonding pattern are predicted correctly, with 28/31 and 26/34 hydrogen bonds correct, respectively (Table S3). Some residue pairs and hydrogen bonds at the ends of the strands are missing due to strand under-prediction. Elav4 was also successful for the pairing of strands 2 and 3, and cannot match strand 1 with 3, most likely because our predicted structure starts midway in strand one.

Folding without secondary structure

We were interested to determine how much of an accurate overall fold was possible without using predicted secondary structure constraints. To do this we followed the same protocol as described for the main experiments with the example of the Ras protein, except in this case, omitting all constraints on residues in predicted secondary structural elements.

Some candidate structures showed reasonable overall topological predictions to the known structure. For example the lowest C_α-rmsd error for the RAS domain family is $\sim$ 5Å C_α-rmsd error to 5p21.pdb, using just the EIC distance constraints without using predicted secondary structures. This is consistent with the notion that a large portion of the information about the correct 3D fold is in the EIC distance constraints, while secondary structure prediction may primarily aid in the process of structure refinement using simulated annealing, perhaps analogous to the physical folding process.

Algorithm performance comparison

Here we examine the performance of the DCA/EIC algorithm across the predicted structures considered and compare the contact accuracy with other with other contact prediction algorithms.

To support the assertion that it is important to use a global model when calculating residue pair correlation scores, we include a comparison of the DCA/EIC algorithm with the BNM algorithm developed by Burger et al., [24] and with two other commonly used local methods, MI and SCA [42,43].
More precisely, in local methods the correlation score of each pair of residues depends only on the observed amino acid distributions for that pair of residues and can be calculated independently of the rest of the alignment. These different measures of pair correlation may have useful applications for certain problems, but our assessment here focuses on their potential for the prediction of which pairs of residues are in spatial proximity in the folded protein. For clarity, we do not make comparison with other local methods for the analysis of correlated mutations, some of which have been expertly compared to each other and to mutual information by Fodor and Aldrich [20], nor with excellent hybrid methods that combine the analysis of correlated mutations with aspects of sequence fit to full or partial 3D structures (fragment search, threading), [44,45]. Contacts predicted by MI, BNM (code kindly provided by Burger and van Nimwegen) and SCA algorithm, were then treated identically to predictions made by the DCA algorithm for the calculations below.

Visualizing contacts in predicted folded structures

The native 3D structure of a protein can be visualized as a network of contacts between amino acids, e.g., as two dimensional 'contact maps' (Figure S2), based on the binary information of whether each pair of residues is spatially proximal ('in contact') or not. We define two residues to be in contact if the minimum all-atom distance (including side chain atoms) between them is less than 5Å. Contact maps provide a visually intuitive window into the three dimensional protein structure and are an excellent way of assessing the ability of an algorithm such as EIC to predict the contacts derived from an experimentally determined structure. Similarly Figures S1, S11 and S12 show the top scoring MI, BNM and SCA pairs respectively for all 15 proteins. For each domain, the black rectangle depicts the boundaries of the PFAM alignment used to predict EIC pairs, residues outside this rectangle cannot be in EIC pairs. Perhaps surprisingly, in some cases, we were able to fold proteins for which the PFAM domain is significantly smaller than the sequence in the PDB structure that we were comparing to.

Analysis of accuracy of inferred contacts in 2D contact space

The simplest assessment of the accuracy of inferred contacts is to count the number of residue pairs predicted to be in contact that are not in close proximity in the crystal structure (Figures S6 and S7). Note that the accuracy of inferred contacts depends on the number of contacts predicted, Nc. Typically the accuracy is excellent for the top-ranked EICs (Figure S7) and decreases as the number of EIC pairs included increases. The optimal cutoff in the number of EICs used is a tradeoff between accuracy of contact prediction and number of inferred distance constraints, and we discuss the optimal choice use in the main text. Beyond $N_C \sim 0.5\text{-}0.7L$, both contact prediction accuracy and accuracy of 3D structure coordinates are a slowly varying function of N_C, such that the predictions are fairly robust with respect to the number of constraints used.

An interesting technical consideration, also important for future improvements of the algorithm, relates to the damage a false positive contributes to prediction accuracy. All true positive residue pairs translate into distance constraints that describe the same subspace, that is the subspace containing the true protein fold. In contrast, each false positive will likely translate to a distance constraint that describes a different subspace than the true subspace. The crucial observation is that while different false positives could describe the same incorrect subspace, it is more likely that each false positive describes a different incorrect subspace. False positives are somehow less damaging if they describe contradictory subspaces, in particular if they are outnumbered by true positives that describe the same correct subspace.

Quantitative measure of false positives

In addition, from the contact maps we observe that false positives are not equal in terms of their detrimental effect on structure prediction (Figure S2). It is plausible that EIC pairs that lie close to

experimentally observed ('real') contacts in the contact map are less damaging than those that are far from the true contacts [2,4,44]. This effect can be quantified for each false positive pair by measuring the distance to the nearest true contact using a simple 2-dimensional Euclidean metric is sequence-position space. We plot the mean of all the distances of each contact for each Nc for all four prediction methods (Figure S9, Appendix A8). The extent of the false positives of predicted contacts plausibly correlates more strongly with the accuracy of 3D structure coordinates.

Quantitative measure of contact prediction spread

Similar to the measure for false positives, we developed a metric for how well spread the predicted contacts are in the protein. Theoretically one could have 100% true positives but they could be clustered in one part of the protein and hence give no information about necessary contacts to determine the 3D structure of the protein. Hence true positive rate alone will not be sufficient. We measure the distance from every true contact (from crystal structure) to every predicted constraint, for each N_C, using a simple 2-dimensional Euclidean metric is sequence-position space. We then plot the mean of this for each Nc, for all 4 contact prediction methods and all proteins,(Figure S10, Appendix A8). The accuracy of distribution of predicted contacts plausibly correlates more strongly with the accuracy of 3D structure coordinates.

Control calculations folding proteins using observed residue contacts

To investigate the performance of the second part of our method, which uses a set of predicted contacts to generated all-atom 3D structures, we reconstructed (not predicted) each protein structure from the set of observed Cα-Cα contacts ('real' contacts) deduced from the crystal structure. Using all residue-residue contacts and adding an upper and lower bound tolerance of 1 Å, we are able to fold the proteins to within 1 Å C_α-rmsd of the PDB structure, which supports the efficacy of our folding pipeline (data not shown). Subsequently we wished to calibrate the performance of our folding methodology using binary contact information, similar to that inferred from the DCA/EIC algorithm, but with perfect accuracy of contacts. From the crystal structure we extracted all residue pairs for which the C_α atoms are within 8 Å of each other, and then discarded pairs with residues within five sequence positions of each other. The corresponding distance constraints require each pair of C_α atoms to be within 7 Å of each other. This is analogous to the distance constraints used in the actual prediction pipeline, but without false positives or false negatives.

The distance constraints implied by these observed Cα-Cα pairs contain significantly more information that the predicted EIC pairs. In addition we included the secondary structure distance constraints constructed from the predicted secondary structure for each domain. Despite this surplus of information and the lack of false positive constraints, we are only able to reconstruct proteins to an accuracy of about 2 - 3 Å C_α-rmsd error (Table S5). This suggests that (1) our method for refining 3D structures can be further improved, as is true for molecular dynamics methods in general, and (2) our sets of EIC pairs perform better at predicting a fold than might be expected, as the error of 3D structure prediction achieved in some cases is near this practical lower bound.

Mapping between PDB and PFAM

We have used PFAM protein family alignments to extract reduced pairs correlations using the DCA algorithm, as this is a well-established and well-maintained database. However, one drawback is that the PFAM domain, and hence the alignment, rarely covers the entire protein domain structure (as in the Protein Data Bank, PDB). This effect is related to the radius of sequence differences coverage in a multiple sequence alignment. Coverage gaps of around 10% at each end of the sequence can provide a significant impediment to folding, especially of residues at the beginning and end of a domain are in 3D contact, but not covered by the sequence alignment.

To test this effect and check the impact on the ability to predict the protein fold, we devised the following control calculation. Previous work has shown that it is possible to fold a protein with around 20% of the actual close distances that are long range in sequence [2,3,4]. For example, for the thioredoxin family

alignment, using the sequence of 1o8w.pdb we were able to fold the structure by choosing 20% of observed residue-residue contacts at random. We then repeated this experiment, restricting ourselves to contacts within the middle 80% of the protein. The number of close contacts used was kept the same, yet the C_α-rmsd error increased from around 2 Å to more than 5 Å when contacts were drawn from just the middle 80% of the protein, data not shown. We conclude that future applications of our methodology to 3D structure prediction should included an assessment of domain boundaries and, possibly, adjustment of parameters in profile alignment methods such as HMMs (hidden Markov models) to increase sequence coverage.

For each PFAM domain there is a range of structures, some of which are present in the PDB. Our folding constraints are restricted to those amino acids in the PDB structure that align to the hidden Markov model (HMM) states of the PFAM domain. PFAM HMM states that did not occur in our chosen PDB structure were considered by the algorithm (to ensure the results are truly global to the alignment) but pair scores involving these states were discarded. Different structures might represent different states of a protein domain, as is the case for the structures 1e6k and 1mb0.

Future work will explore the relationship between protein family sequence and structure spaces across evolution.

References

1. Altschuh D, Lesk AM, Bloomer AC, Klug A (1987) Correlation of co-ordinated amino acid substitutions with function in viruses related to tobacco mosaic virus. J Mol Biol 193: 693-707.
2. Sathyapriya R, Duarte JM, Stehr H, Filippis I, Lappe M (2009) Defining an essence of structure determining residue contacts in proteins. PLoS Comput Biol 5: e1000584.
3. Duarte JM, Sathyapriya R, Stehr H, Filippis I, Lappe M (2010) Optimal contact definition for reconstruction of contact maps. BMC Bioinformatics 11: 283.
4. Vendruscolo M, Kussell E, Domany E (1997) Recovery of protein structure from contact maps. Fold Des 2: 295-306.
5. Locasale JW, Wolf-Yadlin A (2009) Maximum entropy reconstructions of dynamic signaling networks from quantitative proteomics data. PLoS One 4: e6522.
6. Schneidman E, Berry MJ, 2nd, Segev R, Bialek W (2006) Weak pairwise correlations imply strongly correlated network states in a neural population. Nature 440: 1007-1012.
7. Lezon TR, Banavar JR, Cieplak M, Maritan A, Fedoroff NV (2006) Using the principle of entropy maximization to infer genetic interaction networks from gene expression patterns. Proc Natl Acad Sci U S A 103: 19033-19038.
8. Neher E (1994) How frequent are correlated changes in families of protein sequences? Proc Natl Acad Sci U S A 91: 98-102.
9. Göbel U, Sander C, Schneider R, Valencia A (1994) Correlated mutations and residue contacts in proteins. Proteins 18: 309-317.
10. Giraud BG, Heumann JM, Lapedes AS (1999) Superadditive correlation. Physical Review E 59: 4983-4991.
11. Lapedes AS GB, LonChang L,Stromo GD (1999) Correlated Mutations in Models of Protein Sequences:Phylogenetic and Structural Effects. Proceedings of the IMS/AMS International Conference on Statistics in Molecular Biology and Genetics: Monograph Series of the Inst. for Mathematical Statistics, Hayward CA. pp. 236-256.
12. Morcos F. Pagnani A LB, Bertolinod A, Marks DS, Sander C, Zecchina R, Onuchic JN, Hwa T, Weigt M Direct-coupling analysis of residue co-evolution captures native contacts across many protein families.
13. Finn RD, Mistry J, Tate J, Coggill P, Heger A, et al. (2010) The Pfam protein families database. Nucleic Acids Res 38: D211-222.
14. Holm L, Sander C (1996) Mapping the protein universe. Science 273: 595-603.
15. Altschuh D, Vernet T, Berti P, Moras D, Nagai K (1988) Coordinated amino acid changes in homologous protein families. Protein Eng 2: 193-199.
16. Sander C, Schneider R (1991) Database of homology-derived protein structures and the structural meaning of sequence alignment. Proteins 9: 56-68.

17. Neher E (1994) How frequent are correlated changes in families of protein sequences? Proceedings of the National Academy of Sciences of the United States of America 91: 98-102.
18. Horovitz A, Bochkareva ES, Yifrach O, Girshovich AS (1994) Prediction of an inter-residue interaction in the chaperonin GroEL from multiple sequence alignment is confirmed by double-mutant cycle analysis. J Mol Biol 238: 133-138.
19. Lockless SW, Ranganathan R (1999) Evolutionarily conserved pathways of energetic connectivity in protein families. Science 286: 295-299.
20. Fodor AA, Aldrich RW (2004) Influence of conservation on calculations of amino acid covariance in multiple sequence alignments. Proteins 56: 211-221.
21. Skerker JM, Perchuk BS, Siryaporn A, Lubin EA, Ashenberg O, et al. (2008) Rewiring the specificity of two-component signal transduction systems. Cell 133: 1043-1054.
22. Burger L, van Nimwegen E (2008) Accurate prediction of protein-protein interactions from sequence alignments using a Bayesian method. Mol Syst Biol 4: 165.
23. Weigt M, White RA, Szurmant H, Hoch JA, Hwa T (2009) Identification of direct residue contacts in protein-protein interaction by message passing. Proc Natl Acad Sci U S A 106: 67-72.
24. Burger L, van Nimwegen E (2010) Disentangling direct from indirect co-evolution of residues in protein alignments. PLoS Comput Biol 6: e1000633.
25. MacKay DJC (2003) Information theory, inference, and learning algorithms. Cambridge, UK ; New York: Cambridge University Press. xii, 628 p. p.
26. Mezard M, Mora T (2009) Constraint satisfaction problems and neural networks: A statistical physics perspective. J Physiol Paris 103: 107-113.
27. Mora T (2007) Geometry and Inference in Optimization and in Information Theory. Paris: Universite Pairs Sud - Pairs XI.
28. Cocco S, Leibler S, Monasson R (2009) Neuronal couplings between retinal ganglion cells inferred by efficient inverse statistical physics methods. Proc Natl Acad Sci U S A 106: 14058-14062.
29. Monasson VSaR (2009) Small-correlation expansions for the inverse Ising problem. Journal of Physics A: Mathematical and Theoretical 42.
30. Plefka T (1971) Convergence condition of the TAP equation for the infinite-ranged Ising spin glass model. Journal of Physics A: Mathematical and General 15.
31. A Georges JSY (1991) How to expand around mean-field theory using high-temperature expansions. Journal of Physics A: Mathematical and General 24.
32. Janin J (1979) Surface and inside volumes in globular proteins. Nature 277: 491-492.
33. Godzik A, Sander C (1989) Conservation of residue interactions in a family of Ca-binding proteins. Protein Eng 2: 589-596.
34. Rost B, Sander C (1993) Prediction of protein secondary structure at better than 70% accuracy. J Mol Biol 232: 584-599.
35. Jones DT (1999) Protein secondary structure prediction based on position-specific scoring matrices. J Mol Biol 292: 195-202.
36. Havel TF, Kuntz ID, Crippen GM (1983) The combinatorial distance geometry method for the calculation of molecular conformation. I. A new approach to an old problem. J Theor Biol 104: 359-381.
37. Brunger AT, Adams PD, Clore GM, DeLano WL, Gros P, et al. (1998) Crystallography & NMR system: A new software suite for macromolecular structure determination. Acta Crystallogr D Biol Crystallogr 54: 905-921.
38. Pastore A, Atkinson RA, Saudek V, Williams RJ (1991) Topological mirror images in protein structure computation: an underestimated problem. Proteins 10: 22-32.
39. Chothia C (1973) Conformation of twisted beta-pleated sheets in proteins. J Mol Biol 75: 295-302.
40. Chothia C (1973) Conformation of twisted beta-pleated sheets in proteins. Journal of molecular biology 75: 295-302.
41. Richardson JS (1976) Handedness of crossover connections in beta sheets. Proc Natl Acad Sci U S A 73: 2619-2623.
42. Russ WP, Lowery DM, Mishra P, Yaffe MB, Ranganathan R (2005) Natural-like function in artificial WW domains. Nature 437: 579-583.
43. Halabi N, Rivoire O, Leibler S, Ranganathan R (2009) Protein sectors: evolutionary units of three-dimensional structure. Cell 138: 774-786.
44. Ortiz AR, Kolinski A, Rotkiewicz P, Ilkowski B, Skolnick J (1999) Ab initio folding of proteins using restraints derived from evolutionary information. Proteins Suppl 3: 177-185.
45. Wu S, Szilagyi A, Zhang Y (2011) Improving protein structure prediction using multiple sequence-based contact predictions. Structure 19: 1182-1191.
