# Supplementary material for: Protein 3D Structure Computed from Evolutionary Sequence Variation
Source: PLoS One. 2011 Dec 7;6(12):e28766. doi: 10.1371/journal.pone.0028766 (PMC3233603; doi:10.1371/journal.pone.0028766)
Supplement: Table S2 — Conflict resolution between DIs and predicted secondary structure constraints. (DOC) [file pone.0028766.s019.doc]

**Table S2. Conflict resolution between DIs and predicted secondary structure constraints**

| ***Residue 1*** | ***Residue 2*** | ***Description*** | ***Exclusions and justification*** |
| --- | --- | --- | --- |
| H | H | Both residues in same α-helix. | All excluded |
| H-1 | H | Residues just before the helix and in the helix | All excluded – cannot be closer to subsequent residues in helix than first residue in helix is. |
| H-2 | H | Residues two before the helix and in the helix | Cannot be closer to later residues in helix than second residue in the helix is. So can be close to the fifth residue in the helix but we exclude subsequent helix residue. |
| H-3 | H | Residues three before the helix and in the helix | Cannot be closer to later residues in helix than third residue in the helix is. So exclude pairs between this and the 10th subsequent helix residue. |
| H-1 | H+1 | Residue pair that straddles a given helix. | All excluded |
| E | E | Both residues in the same β-strand | All excluded |
| E-1 | E | Residues just before the strand and in the strand | All excluded |
| E-2 | E | Residues two before the strand and in the strand | All excluded |
| E-3 | E | Residues three before the strand and in the strand | All excluded |
| E-4 | E | Residues four before the strand and in the strand | Exclude pair if strand residue is more than four in the the strand. |
| E-1 | E-1 | Residue pair that straddles a strand. | All excluded |
| E-2 | E+2 | Residue pair that straddles a strand. | All excluded if strand 5 residues or longer |
