# Supplementary material for: Protein 3D Structure Computed from Evolutionary Sequence Variation
Source: PLoS One. 2011 Dec 7;6(12):e28766. doi: 10.1371/journal.pone.0028766 (PMC3233603; doi:10.1371/journal.pone.0028766)
Supplement: Table S3 — Distance ranges used for predicted secondary structural elements in folding calculations. (DOC) [file pone.0028766.s020.doc]

**Table S3. Distance ranges used for predicted secondary structural elements in folding calculations**

| ***Residue 1*** | ***Residue 2*** | ***O - O***  ***Distance***  ***Bound*** | ***N-N***  ***Distance***  ***Bound*** | ***O-N***  ***Distance***  ***Bound*** | ***CA-CA***  ***Distance***  ***Bound*** | ***CA-O***  ***Distance***  ***Bound*** | ***CB-CB***  ***Distance***  ***Bound*** |
| --- | --- | --- | --- | --- | --- | --- | --- |
| H | H+1 | 3.07 ± 0.2 | 2.82 ± 0.2 |  | 3.82 ± 0.2 | 4.45 ± 0.2 | 3.6 ± 0.4 |
| H | H+2 | 4.65 ± 0.4 | 4.4 ± 0.4 | 3.3 ± 0.3 | 5.5 ± 0.3 | 5.6 ± 0.4 | 5.15 ± 0.4 |
| H | H+3 | 5.05 ± 0.65 | 5.0 ± 0.6 | 3.95 ± 0.5 | 5.3 ± 0.65 | 5.9 ± 0.8 | 5.2 ± 0.8 |
| H | H+4 | 6.3 ± 0.65 | 6.25 ± 0.8 | 4.3 ± 0.7 | 6.35 ± 0.7 | 7.5 ± 0.7 | 6.35 ± 0.8 |
| H | H+5 | 8.3 ± 0.55 | 8.2 ± 0.5 | 6.1 ± 0.6 | 8.7 ± 0.6 | 9.55 ± 0.6 | 8.55 ± 0.65 |
| H | H+6 | 9.7 ± 0.6 | 9.6 ± 0.55 | 7.95 ± 0.6 | 10.05 ± 0.6 | 10.65 ± 0.65 | 9.9 ± 0.75 |
| H | H+7 | 10.75 ± 0.75 | 10.75 ± 0.65 | 9.05 ± 0.7 | 10.8 ± 0.75 | 11.7 ± 0.75 | 10.8 ± 1.0 |
| H | H+8 | 12.3 ± 0.8 | 12.3 ± 0.7 | 10.3 ± 0.75 | 12.45 ± 0.8 | 13.5 ± 0.75 | 12.45 ± 1.0 |
| E | E+1 | 3.4 ± 0.3 | 3.4 ± 0.3 |  |  | 4.6 ± 0.2 | 4.4 ± 0.5 |
| E | E+2 | 6.45 ± 0.6 | 6.45 ± 0.6 | 4.2 ± 0.5 | 6.6 ± 0.5 | 6.6 ± 0.5 | 7.6 ± 0.7 |
| E | E+3 | 9.5 ± 1.2 | 9.5 ± 1.2 | 7.3 ± 0.8 | 9.7 ± 1.2 | 10.6 ± 1.3 | 9.9 ± 1.3 |
| E | E+4 | 12.5 ± 1.6 | 12.5 ± 1.6 | 10.3 ± 1.3 | 12.6 ± 1.6 | 13.6 ± 1.7 | 12.7 ± 1.7 |
| E | E+5 | 15.4 ± 2.2 | 15.4 ± 2.2 | 13.3 ± 1.9 | 15.5 ± 2.3 | 16.4 ± 2.3 | 15.6 ± 2.3 |
| E | E+6 | 18.0 ± 3.0 | 18.0 ± 3.0 | 16.0 ± 2.6 | 18.1 ± 3.0 | 19.0 ± 3.2 | 18.2 ± 3.1 |
| E | E+7 | 20.5 ± 4.0 | 20.5 ± 4.0 | 18.6 ± 3.6 | 20.5 ± 4.1 | 21.3 ± 4.2 | 20.6 ± 4.1 |
