# Supplementary material for: Protein 3D Structure Computed from Evolutionary Sequence Variation
Source: PLoS One. 2011 Dec 7;6(12):e28766. doi: 10.1371/journal.pone.0028766 (PMC3233603; doi:10.1371/journal.pone.0028766)
Supplement: Table S4 — β sheet detection in predicted structures. (DOC) [file pone.0028766.s021.doc]

**Table S4. β sheet detection in predicted structures**

| Uniprot name | Predicted / correct number of strand pairs | Predicted / correct number of registered residue pairs * |
| --- | --- | --- |
| RASH_HUMAN | 5 / 5 | 26 / 30 |
| CADH1_HUMAN | 5 / 5 | 28 / 31 |
| ELAV4_HUMAN | 1 / 2 | 3 / 5 |

*For correct pairs
