# Supplementary material for: Protein 3D Structure Computed from Evolutionary Sequence Variation
Source: PLoS One. 2011 Dec 7;6(12):e28766. doi: 10.1371/journal.pone.0028766 (PMC3233603; doi:10.1371/journal.pone.0028766)
Supplement: Table S5 — Control calculations testing real distances. (DOC) [file pone.0028766.s022.doc]

**Table S5. Control calculations testing real distances**

| ***Protein name*** | ***Domain name*** | ***Number C_α_-C_α_ distances  <8 Å*** | ***Best  C***_α_ ***rmsd*** | ***Number C***_α_***- C***_α_ ***distances  < 10 Å*** | ***Best  C***_α_***-rmsd*** | ***Number of EIC pairs in structures***  ***in Supp Figs*** | ***Number***  ***aligned residues*** | ***Fold type*** | ***Ref. PDB*** |
| --- | --- | --- | --- | --- | --- | --- | --- | --- | --- |
| HRAS | RAS | 302 | 1.9 | 777 | 3.9 | 120 | 148 | αβ | 5p21 |
| Ribo-nuclease H1 | RNaseH | 313 | 2.5 | 756 | 4.0 | 80 | 103 | αβ | 1f21 |
| CheY | Response regulator receiver | 178 | 2.0 | 453 | 2.8 | 110 | 110 | αβ | 1mb0 |
| CheY | Response regulator receiver | 192 | 5.8 | 527 | 6.7 | 110 | 115 | αβ | 1e6k |
| Trypsin | trypsin | 575 | 4.2 | 1380 | 4.2 | 170 | 192 | β | 3tgi |
| Cadherin | cadherin | 220 | 2.3 | 506 | 4.4 | 70 | 91 | β | 2o72 |
| Yes | SH3 | 125 | 2.3 | 272 | 4.2 | 40 | 40 | β | 2hda |
| Elav4 | RRM | 138 | 2.1 | 340 | 2.6 | 70 | 67 | αβ | 1g2e |
| PCBP1 | KH | 101 | 3.3 | 271 | 3.3 | 30 | 63 | αβ | 1wvn |
| O45418 | FKBP | 282 | 3.6 | 572 | 3.6 | 50 | 87 | αβ | 1r9h |
| OmpR | Trans reg C | 155 | 2.5 | 394 | 3.1 | 30 | 67 | α | 1odd |
| Spbt2 | Calponin homology | 120 | 2.1 | 387 | 3.3 | 40 | 87 | α | 1bkr |
| BPTI | trypsin inhibitor | 112 | 2.2 | 262 | 2.7 | 30 | 52 | α β | 5pti |
